# Targeting amphiregulin (AREG) derived from senescent stromal cells diminishes cancer resistance and averts programmed cell death 1 ligand (PD‐L1)‐mediated immunosuppression

**DOI:** 10.1111/acel.13027

**Published:** 2019-09-07

**Authors:** Qixia Xu, Qilai Long, Dexiang Zhu, Da Fu, Boyi Zhang, Liu Han, Min Qian, Jianming Guo, Jianmin Xu, Liu Cao, Y. Eugene Chin, Jean‐Philippe Coppé, Eric W.‐F. Lam, Judith Campisi, Yu Sun

**Affiliations:** ^1^ Institute of Health Sciences, Shanghai Jiao Tong University School of Medicine and Shanghai Institutes for Biological Sciences Chinese Academy of Sciences Shanghai China; ^2^ Department of Urology, Zhongshan Hospital Fudan University Shanghai China; ^3^ Department of General Surgery, Zhongshan Hospital Fudan University Shanghai China; ^4^ Central Laboratory for Medical Research, Shanghai Tenth People’s Hospital Tongji University School of Medicine Shanghai China; ^5^ CAS Key Laboratory of Tissue Microenvironment and Tumor, Shanghai Institute of Nutrition and Health, Shanghai Institutes for Biological Sciences, University of Chinese Academy of Sciences Chinese Academy of Sciences Shanghai China; ^6^ Key Laboratory of Medical Cell Biology China Medical University Shenyang China; ^7^ Institute of Biology and Medical Sciences Soochow University Medical College Suzhou Jiangsu China; ^8^ Department of Laboratory Medicine, Helen Diller Family Comprehensive Cancer Center University of California San Francisco CA USA; ^9^ Department of Surgery and Cancer Imperial College London London UK; ^10^ Buck Institute for Research on Aging Novato CA USA; ^11^ Lawrence Berkeley National Laboratory Life Sciences Division Berkeley CA USA; ^12^ Department of Medicine and VAPSHCS University of Washington Seattle WA USA

**Keywords:** aging, amphiregulin, cancer resistance, clinical biomarker, combinational treatment, programmed cell death 1 ligand, senescence‐associated secretory phenotype, stroma

## Abstract

Aging is characterized by a progressive loss of physiological integrity, while cancer represents one of the primary pathological factors that severely threaten human lifespan and healthspan. In clinical oncology, drug resistance limits the efficacy of most anticancer treatments, and identification of major mechanisms remains a key to solve this challenging issue. Here, we highlight the multifaceted senescence‐associated secretory phenotype (SASP), which comprises numerous soluble factors including amphiregulin (AREG). Production of AREG is triggered by DNA damage to stromal cells, which passively enter senescence in the tumor microenvironment (TME), a process that remarkably enhances cancer malignancy including acquired resistance mediated by EGFR. Furthermore, paracrine AREG induces programmed cell death 1 ligand (PD‐L1) expression in recipient cancer cells and creates an immunosuppressive TME via immune checkpoint activation against cytotoxic lymphocytes. Targeting AREG not only minimized chemoresistance of cancer cells, but also restored immunocompetency when combined with classical chemotherapy in humanized animals. Our study underscores the potential of in vivo SASP in driving the TME‐mediated drug resistance and shaping an immunosuppressive niche, and provides the proof of principle of targeting major SASP factors to improve therapeutic outcome in cancer medicine, the success of which can substantially reduce aging‐related morbidity and mortality.

## 1 INTRODUCTION

Although the development of advanced human malignancies substantially restrains the spectrum of therapeutic options, numerous data suggested that clinical failure is indeed intertwined with drug resistance (Robey et al., 2018). To date, the lack of a sustained treatment response is largely attributed to either intrinsic or acquired resistance of cancer cells, the mechanisms of which frequently implicate a preexisting tumor microenvironment (TME), a pathological entity that functionally supports the outgrowth of resistant clones even in clinical settings (Chen et al., 2018; Gandhi & Das, 2019).

Systematic investigation of drug resistance across tumor types, and even therapeutic categories, can enable novel insights into cancer biology. Despite the initial response of tumors to most clinical regimens, the efficacy of subsequent interventions gradually vanishes. Off‐target effects of cytotoxic agents frequently trigger irreparable damage in benign stromal cells surrounding the tumor foci (Faget, Ren, & Stewart, 2019; Sun, Coppe, & Lam, 2018), and generate a large number of senescent cells that display a senescence‐associated secretory phenotype (SASP; Acosta et al., 2008; Coppe et al., 2008; Kuilman et al., 2008). Although the SASP can favor tissue homeostasis by supporting tissue repair, wound healing, and immunosurveillance (Salama, Sadaie, Hoare, & Narita, 2014), more studies pinpoint functional implications of the SASP in age‐related pathologies (Childs et al., 2016; Jeon et al., 2017). We and others have reported that secretion of a myriad of soluble factors including cytokines, chemokines, growth factors, and proteases generated by the SASP promotes chemoresistance of surviving cancer cells after early waves of administration, specifically in genotoxic settings (Gilbert & Hemann, 2010; Obenauf et al., 2015; Sun et al., 2012). While the SASP is entering the spotlight of intensive research in multiple human diseases, it remains unknown whether specific components of the full SASP spectrum can drive cancer resistance as major forces under treatment pressure. Exploration of the functional mechanisms supporting expression of such “major” SASP effectors, and establishment of therapeutic strategies to circumvent adverse effects of the SASP in a treatment‐damaged TME, represents attractive, promising but challenging issues.

Distinct from conventional anticancer treatments including chemotherapy that have relatively limited efficacy and durability, immunotherapy takes advantage of a patient's own immune system and exhibits salient efficacy for many cancer types. Despite the unprecedented tumor regression and long‐term survival benefit observed with agents against programmed cell death 1 (PD‐1) or programmed cell death 1 ligand (PD‐L1), a large portion of patients do not benefit and many responders eventually relapse (Kim, Herbst, & Chen, 2018). Continued efforts to minimize resistance against immune checkpoint blockade (ICB) require a clear understanding of cancer resistance and should well precede current avenues using random combinations with available therapeutic modalities. Retrospective studies of patient populations have discovered that there are different types of TME, whose classification can be improved by next‐generation technologies to inform success or failure of current ICB agents and encourage development of advanced immunotherapeutics (Binnewies et al., 2018).

The SASP can remodel the TME via paracrine actions and accelerate disease progression (Demaria et al., 2017; Obenauf et al., 2015). Although some SASP components are cytokines or chemokines per se, such as IL‐8 and CXCL3, diverse growth factors including amphiregulin (AREG) are produced by senescent human stromal cells (Coppe et al., 2008; Sun et al., 2012), suggesting that the impact of the SASP on tumor progression could be complicated and multidimensional. AREG, an epidermal growth factor (EGF) receptor ligand, is implicated in multiple cancer types and potently enhances malignant development in both primary and metastatic lesions (Xu, Chiao, & Sun, 2016). Using a colitis and tumor vaccination model, a group showed that mast cell‐derived AREG potentiates the immunosuppressive competency of regulatory T (Treg) cells, thus establishing a link between mast cells and Treg cells in the TME and suggesting a potential value of perturbing the associated mechanism to improve therapeutic efficacy of EGFR‐targeting agents in cancer clinics (Zaiss et al., 2013). However, a precise cell type‐specific pattern of AREG production in response to anticancer treatments and its pathological implications in drug response remain poorly defined. In this study, we discovered the unique contribution of stromal AREG to tumor malignancy, especially drug resistance acquired from the treatment‐damaged TME, and demonstrated the potential of targeting AREG in combination with classic chemotherapy to improve therapeutic index. AREG represents both a soluble factor that is targetable to circumvent advanced conditions including acquired resistance against both conventional and ICB treatments, and a distinct SASP biomarker to timely monitor the in vivo response of the TME in clinical settings. Since AREG is a hallmark indicator of the SASP development and holds both therapeutic and prognostic values, harnessing such a “major” TME factor in synergy with conventional agents may represent a novel therapeutic paradigm to enhance patient outcome in future clinics.

## 2 RESULTS

### 2.1 AREG expression increases in senescent human stromal cells produced by genotoxic treatments

**Figure 1:**
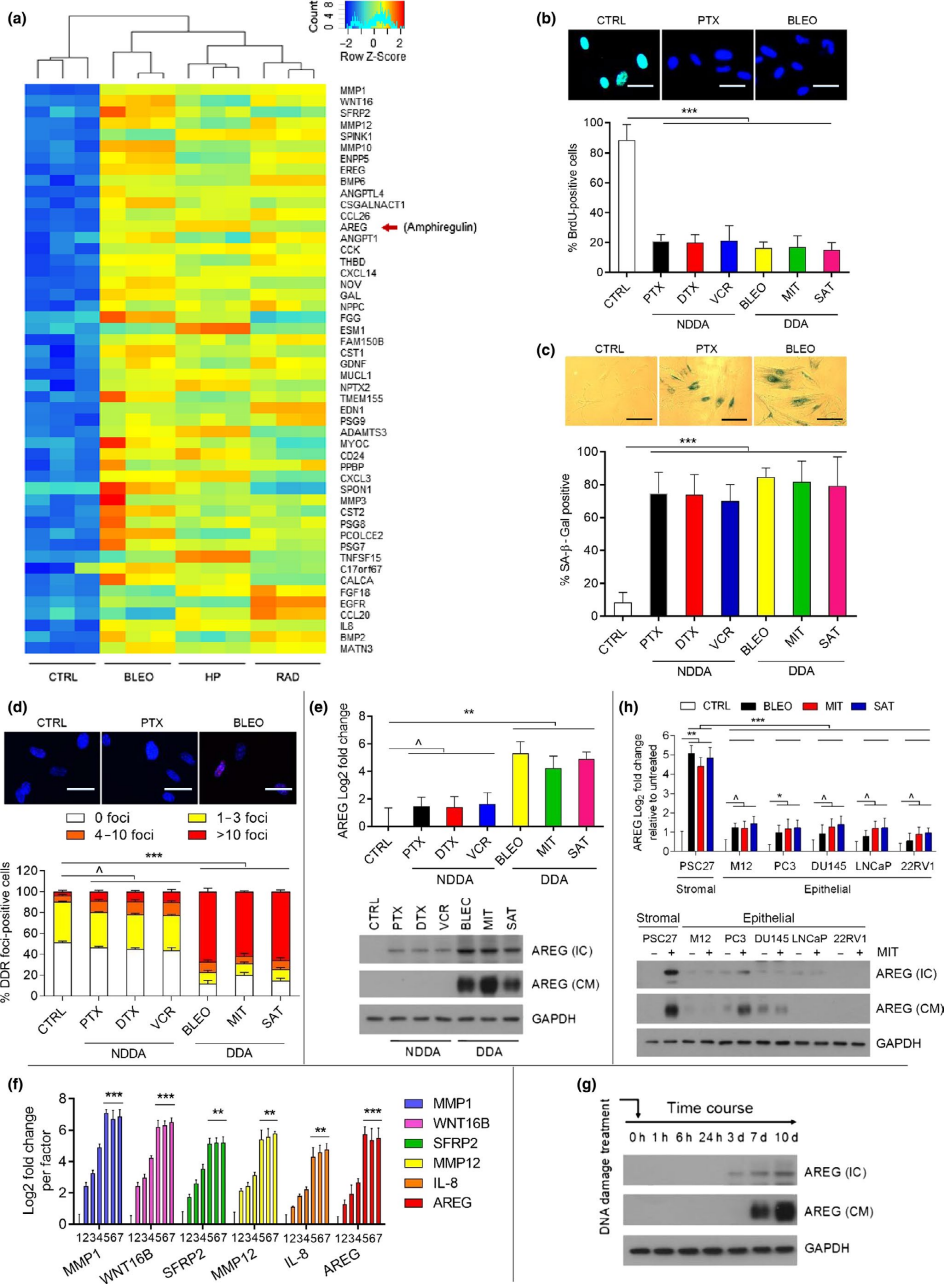
Genotoxic agents induce cellular senescence and the SASP including AREG. (a) Gene expression change in primary normal human prostate stromal cells (PSC27) profiled by microarray analysis. CTRL, control. BLEO, bleomycin. HP, hydrogen peroxide. RAD, ionizing radiation. Red arrow, AREG. Top 50 genes are displayed. (b) BrdU staining performed 7 days after treatment of PSC27 cells by different agents including paclitaxel (PTX), docetaxel (DTX), and vincristine (VCR) as non‐DNA‐damaging agents (NDDA), with bleomycin (BLEO), mitoxantrone (MIT), and satraplatin (SAT) applied as DNA‐damaging agents (DDA) in parallel assays. Top, pictures from PTX and BLEO treatments shown as representative images of each agent type. Scale bars = 15 μm. Bottom, statistics of staining positivity. (c) SA‐β‐Gal staining of PSC27 cells treated by various agents depicted in (b). Cells were stained 7 days after in vitro treatments. Top, representative images. Scale bars = 15 μm. Bottom, statistics of staining positivity. (d) Immunofluorescence staining of DNA damage foci (DDR) (γH2AX) of PSC27 cells treated by various agents. DDRs were quantitatively classified into four subcategories including 0 foci, 1 ~ 3 foci, 4 ~ 10 foci, and >10 foci per cell. Top, representative images. Scale bars = 15 μm. Bottom, comparative statistics. (e) AREG expression after treatment of PSC27 cells by various agents. Cell lysates were collected for measurement 7 days after treatment. Top, quantitative RT–PCR (qRT–PCR) assays, signals normalized to CTRL. Bottom, immunoblots. IC, intracellular; CM, conditioned media; GAPDH, loading control. (f) Time course expression of representative SASP factors (MMP1, WNT16B, SFRP2, MMP12, IL‐8, and AREG) after bleomycin treatment. Experimentally, qRT–PCR assays were performed with stromal cells treated with 50 μg/ml bleomycin and collected at a series of time points including 0, 1, 3, 5, 7, 10, and 15 day(s) post‐treatment, respectively, which are represented by numerical numbers 1, 2, 3, 4, 5, 6, and 7 labeled on the X‐axis. (g) Immunoblot analysis of PSC27 cells collected at various time points post‐treatment with bleomycin to reveal the expression pattern of AREG at protein level. IC, intracellular; CM, conditioned media; GAPDH, loading control. (h) Comparative analysis of AREG transcript expression in stromal cells (PSC27) versus neoplastic epithelial cells (M12, PC3, DU145, LNCaP, and 22RV1). Top, qRT–PCR assays, signals normalized to untreated sample per cell line. Bottom, immunoblots. IC, intracellular; CM, conditioned media; GAPDH, loading control. Data are representative of three independent experiments. *p* Values were calculated by one‐way (b, c, e, f, h) and two‐way (d) ANOVA (^*p* > .05; **p < .01; and ***p < .001). Agilent microarray data of (a) were adapted from Sun et al. with permission from Nature Medicine, copyright 2012

Former studies showed that AREG is upregulated in response to diverse stimuli, but is mainly associated with immune cell populations activated in type 2 inflammatory responses to restore tissue integrity and homeostasis (Zaiss, Gause, Osborne, & Artis, 2015). In cancer cells carrying EGFR mutants such as T790M, AREG expression is markedly elevated, but mostly limited to lung malignancies (Regales et al., 2009; Taverna et al., 2017). However, the vast majority of AREG biology has been focused on immune and neoplastic cells, leaving the tissue‐resident stroma that contains various benign cell types largely overlooked, particularly their dynamics in clinical conditions. We recently found that the prostate stromal cell line PSC27, comprising mainly fibroblasts but with a minor percentage of nonfibroblast cell lineages including endothelial cells and smooth muscle cells, produces a large number of SASP factors after exposure to cytotoxic insults, specifically genotoxic chemotherapy or ionizing radiation (Sun et al., 2012). Of note, AREG emerged as one of the major SASP factors as previously demonstrated by our microarray data (Figure 1a; Sun et al., 2012). To expand the study, we applied a subset of DNA‐damaging agents (DDA) including bleomycin (BLEO), mitoxantrone (MIT), and satraplatin (SAT), and a group of non‐DNA‐damaging agents (NDDAs) including paclitaxel (PTX), docetaxel (DTX), and vincristine (VCR) to treat stromal cells. All agents caused reduced DNA synthesis (BrdU incorporation) and increased lysosomal activity (SA‐β‐Gal positivity), indicating typical cell cycle arrest accompanied by cellular senescence (Figure 1b, c). However, only the DDA group induced intensive DDR activities (Figure 1d). Subsequent examination at both mRNA and protein levels confirmed the inducible nature of AREG in response to genotoxic stress, a process accompanied by extracellular release (*p* < .01 at transcript level; Figure 1e). Notably, AREG transcript expression largely phenocopied other hallmark SASP factors including MMP1, WNT16B, SFRP2, MMP12, and IL‐8, which exhibited a gradual increment until approaching a platform within 7–8 days after treatment (*p* < .01 for SFRP2, MMP12, and IL‐8, *p* < .001 for others; Figure 1f). Intracellular expression and extracellular secretion of AREG protein largely paralleled its transcriptional expression, each manifesting a time‐dependent increase after genotoxic stress (Figure 1g).

Expression assessment among several cell lines of prostate origin disclosed that stromal cells are indeed more AREG‐inducible than cancer epithelial cells, implying a special mechanism that supports AREG production in prostate stromal cells post‐DNA damage (*p* < .01 for stromal, *p* > .05 for most cancer epithelial lines; Figure 1h). The responsiveness to genotoxicity and differential expression pattern between stromal and cancer epithelial lines were confirmed in an alternative group of cell lines of human breast origin, including a stromal line HBF1203 and several cancer cell lines regardless of their malignancy properties (Figure S1a–e). To further expand, we examined human diploid fibroblast (HDF) and mouse embryonic fibroblast (MEF) lines, and found a similar DNA damage‐inducible pattern of AREG (Figure S1f–i). The data consistently suggest an organ‐ or tissue type‐independent nature of AREG induction upon cellular senescence in response to genotoxic stress. We also noticed that overexpression of AREG itself in these lines was insufficient to induce cellular senescence or result in the SASP development, suggesting limited influence of this soluble factor on stromal cell senescence (Figure S1f–i).

### 2.2 Stromal AREG expression predicts adverse clinical outcome after chemotherapy

**Figure 2:**
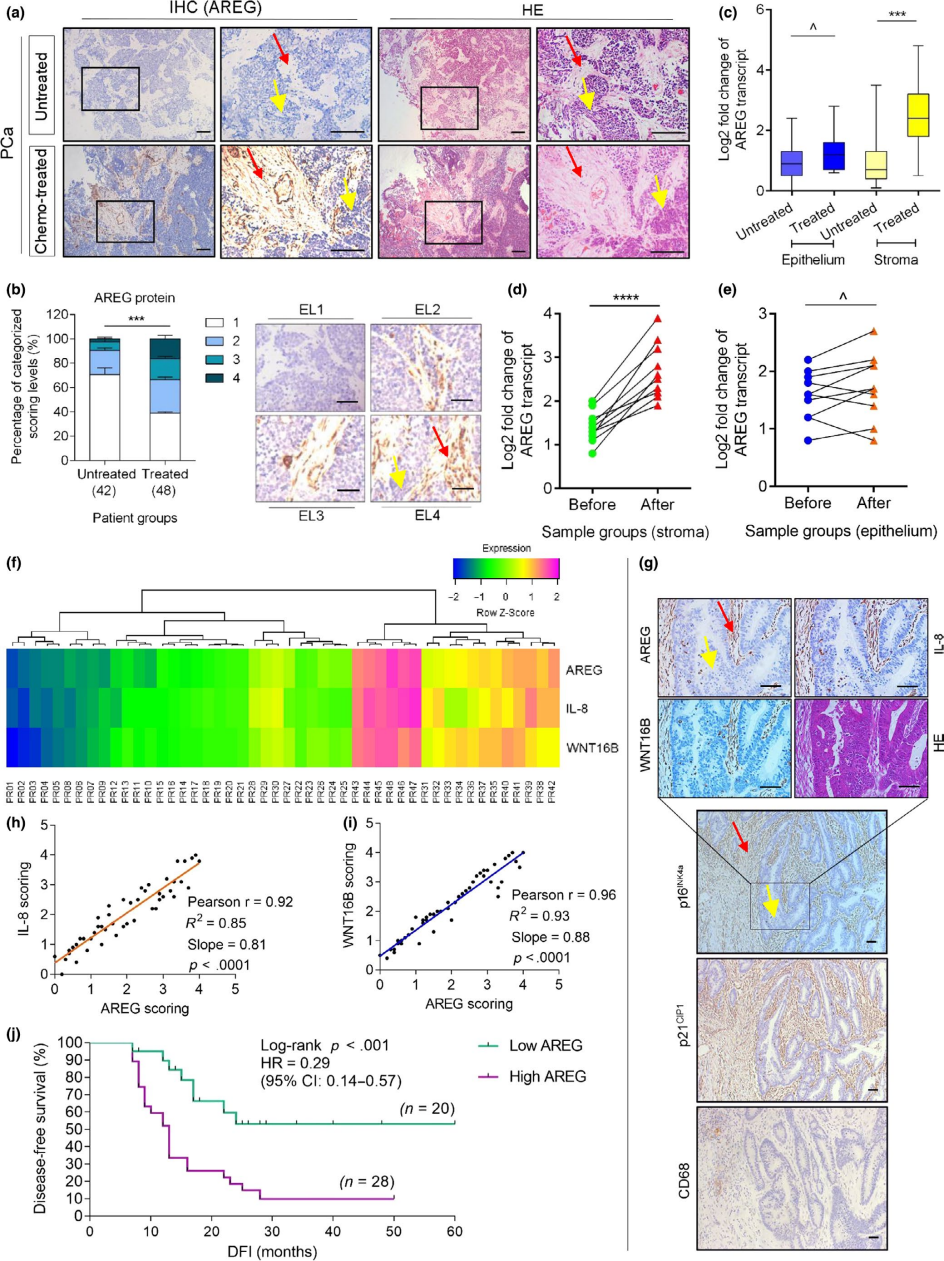
AREG is expressed in human prostate stroma after chemotherapy and correlated with adverse clinical outcome. (a) Representative images of AREG expression in biospecimens of human prostate cancer (PCa) patients after histological examination. Left, immunohistochemical (IHC) staining. Right, hematoxylin and eosin (HE) staining. In each staining set, top tissues, untreated; bottom tissues, treated. Rectangular regions selected in the left images per staining zoomed on the right, with all samples acquired from the same patient. Scale bars = 200 μm. (b) Pathological assessment of stromal AREG expression in PCa samples (untreated, 42 patients; treated, 48 patients). Patients were pathologically assigned into four categories per IHC staining intensity of AREG in the stroma. (1) negative; (2) weak; (3) moderate; and (4) strong expression. Left, statistical comparison of the percentage of each category. Right, representative images of each category regarding AREG signals. EL, expression level. Scale bars = 100 µm. (c) Boxplot summary of AREG transcript expression by qRT–PCR analysis upon laser capture microdissection (LCM) of cells from tumor and stroma, respectively. Signals normalized to the lowest value in the untreated cancer epithelium group, comparison performed between untreated (42 patients) and treated (48) samples per cell lineage. (d) Comparative analysis of AREG expression at transcription level between stromal cells collected before and after chemotherapy. Each dot represents an individual patient, with the data of “before” and “after” connected to allow direct assessment of AREG induction in the same individual patient. Samples from 10 patients were selected for assays. (e) Comparative analysis of AREG expression at transcription level in cancer epithelial cells collected from the same individual patients as described in (d). (f) Pathological correlation between AREG, IL‐8, and WNT16B in the stroma of PCa patients after chemotherapy. Scores were from the assessment of molecule‐specific IHC staining, with expression levels colored to reflect low (blue) via modest (green) and fair (yellow) to high (purple) signal intensity. Columns represent individual patients, and rows represent different SASP factors. Totally, 48 patients treated by chemotherapy were analyzed, with scores of each patient averaged from three independent pathological readings. (g) Representative images of AREG, IL‐8, and WNT16B expression in the TME upon IHC staining of biospecimens from 48 post‐treatment PCa patients. The p16^INK4a^ and p21^CIP1^ images are shown to allow an overview of senescent cells arising at the tumor foci post‐treatment. CD68 was stained to locate human macrophages in the TME. Scale bars = 100 μm. (h) Statistical correlation between AREG and IL‐8 scores (Pearson's analysis, r = 0.98, *p* < .0001) in the 48 tumors with matching protein expression data. (i) Statistical correlation between AREG and WNT16B scores (Pearson's analysis, *r* = .96, *p* < .0001) in the same tumors as described in (h). (j) Kaplan–Meier analysis of PCa patients. Disease‐free survival (DFS) stratified according to AREG expression (low, average score <2, turquoise line, *n* = 20; high, average score ≥2, purple line, *n* = 28). DFS represents the length (months) of period calculated from the date of PCa diagnosis to the point of first‐time disease relapse. Survival curves generated according to the Kaplan–Meier method, with *p* value calculated using a log‐rank (Mantel–Cox) test. Data in all bar plots are shown as mean ± *SD* and representative of three biological replicates. Red arrows indicate stroma, and yellow arrows indicate cancer epithelium (a, b, g). *p* values were calculated by Student's *t* test (c, d, e), one‐way ANOVA (b), and log‐rank test (j) (^*p* > .05; ***p < .001; and ****p < .0001). HR, hazard ratio

The in vitro findings prompted us to further determine whether AREG is produced by the TME, a pathological entity that comprises numerous benign stromal cells. We investigated the biospecimens of a cohort of prostate cancer (PCa) patients who developed primary tumors and underwent genotoxic chemotherapy. Surprisingly, AREG was found significantly expressed in the prostate tissues of patients after chemotherapy, but not before (Figure 2a). In line with our in vitro data, upregulated AREG was generally localized in the stroma, in sharp contrast to the adjacent cancer epithelium which had limited or no staining.

AREG synthesis in patient tissues post‐ versus prechemotherapy was quantitatively consolidated by a pre‐established pathological appraisal procedure that allowed precise assessment of a target protein expression according to its immunohistochemistry (IHC) staining intensity (*p* < .001; Figure 2b). Transcript analysis upon laser capture microdissection (LCM) of cell lineages from the primary tissues indicated that AREG was more readily induced in the stromal rather than cancer cell populations (*p* < .001 vs. *p* > .05; Figure 2c). To substantiate AREG inducibility in vivo, we analyzed a subset of patients whose pre‐ and postchemotherapy biospecimens were both accessible, and found remarkably upregulated AREG in the stroma, but not cancer epithelium, of each individual after chemotherapy (Figure 2d,e). Further, we noticed AREG expression dynamics in the damaged TME essentially in parallel to those of IL‐8 and WNT16B, two canonical SASP factors (Figure 2f). Expression sites of these factors were largely overlapping with those of senescent cells (p16^INK4a+^ and p21^CIP1^) in the TME, excluding cancer cells, which may have survived and progressively repopulated after therapy (Figure 2g; Sun et al., 2012). Cellular senescence was developed pronouncedly in the stromal compartment, which also includes immune cells such as a limited number of macrophages. The correlation between AREG and IL‐8/WNT16B expression in the damaged TME was further substantiated by pathological evaluation of their expression scores in post‐treatment patients (Figure 2h,i). More importantly, the Kaplan–Meier analysis of PCa patients stratified according to AREG amount in tumor stroma suggested a significant but negative correlation between AREG protein level and disease‐free survival (DFS) in the treated cohort (*p* < .001, log‐rank test; Figure 2j).

The distinct pathological properties of AREG were reproduced by an extended study that recruited individual cohorts of human breast cancer (BCa) patients (*p* < .001, survival comparison by log‐rank test; Figure S2a‐i). Of note, Cox proportional hazard regression analyses of these patients indicated significant correlation of stromal AREG with poor cancer survival (Tables S1–S4). Thus, our data consistently suggested that AREG expression in tumor stroma acts as an SASP‐associated independent predictor of prognosis, which is exploitable in stratifying the risk of disease relapse and clinical mortality of post‐treatment patients, and that AREG production by the stroma may have a causal role in tumor progression.

### 2.3 Paracrine AREG generates oncogenic effects by activating EGFR‐mediated pathways in recipient cancer cells

We next examined the effect of stromal AREG on PCa cell lines via co‐culture with conditioned media (CM) derived from stromal cells, an assay involving PSC27 sublines stably overexpressing or subsequently losing AREG (Figure S3a). Upon treatment with the CM from AREG‐positive cells (PSC27^AREG^), we observed significantly increased proliferation of a group of established PCa cell lines including PC3, DU145, LNCaP, and M12 (*p* < .01; Figure S3b). Indicative of advanced cell malignancy, the migration and invasion activities of PCa cells were considerably enhanced in the presence of stromal AREG (*p* < .01 for most migration and invasion assays; Figure S3c,d). However, the malignancy‐promoting effects of AREG on cancer behaviors were almost completely abrogated upon AREG depletion from PSC27 (Figure S3b–d). More importantly, AREG enhanced the resistance of PCa cells against MIT, a DNA‐targeting chemotherapeutic agent for human malignancies including PCa (Bergstrom et al., 2017; Eisenberger et al., 2017; Figure S3e). To further confirm the contribution of AREG to cancer cell phenotypic alterations by specifically eliminating AREG protein itself, we generated a monoclonal antibody (AREG mAb) with high competency in recognizing free AREG in culture conditions (Figure S3f). The data from AREG mAb‐relevant assays closely resembled those from AREG knockdown experiments (Figure S3b‐e), thus excluding the possibility that AREG itself induces expression of other SASP factors in stromal cells.

Further analysis indicated that MIT induced cleavage of caspase 3 in cancer cells, a process remarkably weakened by AREG but reversible upon elimination of AREG from stromal cells (Figure S3g), implying that AREG drives cancer resistance largely via a caspase‐counteracting mechanism, which dampens caspase 3 activation by its self‐cleavage. We further applied QVD‐OPH and ZVAD‐FMK, two potent pan‐caspase inhibitors, and PAC1 and gambogic acid (GA), two caspase activators, to individually treat PC3 cells before MIT exposure. Cell apoptosis was substantially attenuated when QVD‐OPH or ZVAD‐FMK was used, even in the presence of AREG (*p* < .001; Figure S3h). However, once the pro‐caspase‐activating compound PAC1 or GA was added to the media, apoptosis index was markedly increased, offsetting the anti‐apoptosis effect of AREG (*p* < .01 in the presence of AREG; Figure S3h). The data were reproduced when docetaxel (DOC), another chemotherapeutic drug typically inhibiting microtubule depolymerization, was applied to the system (Figure S3i). Thus, our results consistently demonstrate that stromal AREG perturbs caspase‐dependent apoptosis, underlying its resistance‐boosting capacity via paracrine influence on recipient cancer cells.

**Figure 3:**
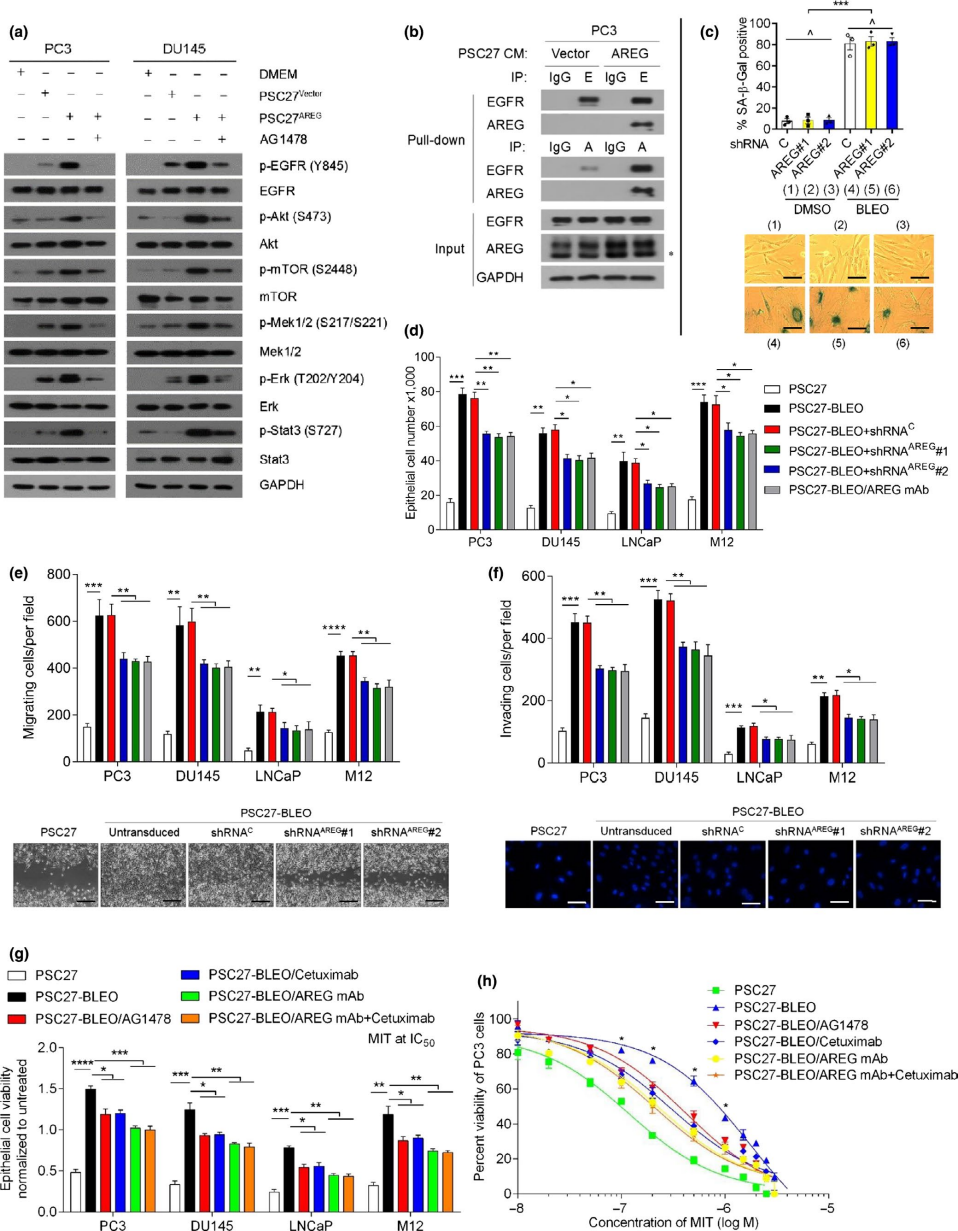
Stromal AREG significantly modifies phenotypes of prostate cancer cells. (a) Immunoblot analysis of EGFR‐associated pathways in PC3 and DU145 cells treated by the CM from PSC27 cells transduced with the empty vector or AREG construct, or alongside the EGFR inhibitor AG1478 (6 μM). Antibodies of p‐EGFR (Y845), p‐Akt (S473), p‐mTOR (S2448), p‐Erk (T202/Y204), and p‐Stat3 (S727) were applied to probe the individual molecules. Total protein per molecule and GAPDH were used as loading control. (b) Immunoprecipitation (IP) followed by immunoblot assay of EGFR and AREG in the whole‐cell lysates of PC3 treated by the CM of PSC27^Vector^ and PSC27^AREG^ for 3 days. Antibodies including IgG, anti‐EGFR, and anti‐AREG were used for IP, with EGFR and AREG in inputs analyzed simultaneously. E, anti‐EGFR. A, anti‐AREG. GAPDH, loading control. (c) PSC27 cells were transduced with constructs encoding scramble or AREG‐specific shRNAs to make stable lines. Cells were then treated with either DMSO or BLEO and subjected to SA‐β‐Gal assay. Upper, comparative statistics. Lower, representative images of SA‐β‐Gal‐stained cells. (d) PCa cells were treated with the CM from PSC27 sublines for 3 days and subject to cell proliferation assay. Native and shRNA‐transduced PSC27 cells as indicated were treated by BLEO, with the CM collected 7 days after treatment, and used for PC3 culture. Alternatively, an anti‐AREG monoclonal antibody was employed to neutralize AREG in the CM before cancer cell phenotypic assays (as a control, also for e and f). (e) Migration assay of PCa cells seeded within transwells in 6‐well plates, with cells cultured for 3 days in the CM from PSC27 sublines depicted in (d). Bottom, representative images of PC3 cell migration measured via wound healing assay at 72 hr after cultured with individual CM. Scale bars = 100 μm. (f) Invasiveness appraisal of PCa cells across the transwell membrane upon culture with the CM from PSC27 sublines. Bottom, representative images of PC3 cell invasion across the transwell measured at 72 hr after cultured with individual CM. Scale bars = 20 μm. (g) Chemoresistance assay of PCa cells cultured with the CM from PSC27 sublines described in (d). MIT was applied at the concentration of IC50 value predetermined per cell line. AG1478 (6 μM), cetuximab (50 μg/ml), or AREG mAb (1 μg/ml) was applied alongside with PSC27 CM. (h) Dose–response curves (nonlinear regression/curve fit) plotted from drug‐based survival assays of PC3 cells cultured with the CM of PSC27 native or damaged by bleomycin (PSC27‐BLEO), and concurrently treated by a wide range of concentrations of MIT. AG1478 (6 μM), cetuximab (50 μg/ml), and/or AREG mAb (1 μg/ml) were applied with PSC27 CM. Data are representative of three independent experiments, with three technical replicates run per cell‐based experiment. *P* values were calculated by Student's *t* test (c, d, e, f, g) (^*p* > .05; *p < .05; **p < .01; ***p < .001; and ****p < .0001)

We next explored the mechanism supporting AREG to confer the pro‐survival advantage on cancer cells. Since AREG protein encompasses an EGF‐like domain (aa 141–181) which has six spatially conserved cysteines forming disulfide bridges and the 3‐looped structure defining the EGF family (Berasain & Avila, 2014), we first determined the function of AREG as an EGF‐like growth factor via in vitro assays. Upon treatment of PCa cells with the CM from AREG‐expressing PSC27 (PSC27^AREG^), we observed rapid phosphorylation of EGFR (Y845), Akt (S473), and mTOR (S2448), suggesting activation of the PI3K/Akt/mTOR pathway by AREG (Figure 3a). Further, phosphorylation of Mek (S217/S221), Erk (T202/Y204), and Stat3 (S727) was identified, indicating simultaneous activation of a MAPK pathway in these cells. We then used tyrphostin AG1478, a selective receptor tyrosine kinase (RTK) inhibitor preferentially targeting EGFR (El‐Hashim et al., 2017). Upon addition of AG1478, AREG‐induced EGFR phosphorylation was abolished, with reduced activation of both Akt/mTOR and Mek/Erk/Stat3 axes (Figure 3a). Thus, AREG‐triggered activation of these two signaling pathways was essentially mediated by EGFR, although functional involvement of other RTKs cannot be excluded. We next performed immunoprecipitation (IP) with an AREG‐specific antibody after treatment of PCa cells with AREG^+^ stromal media. A strong interaction between AREG and EGFR was evidenced by the prominent signal in the precipitate pulled down by anti‐AREG rather than the control IgG, with IP signal much stronger in AREG^+^ CM‐treated cells than control (Figure 3b).

We interrogated whether AREG, a soluble factor expressed in the entire stromal SASP spectrum, generates remarkable effects on the phenotypes of stromal cells per se. Of note, AREG depletion from PSC27 cells neither delayed nor accelerated cellular senescence, as indicated by SA‐β‐Gal assay (Figure 3c). However, we noticed that AREG elimination from PSC27 markedly dampened pathway activation induced by the full‐blown SASP of damaged stromal cells, suggesting AREG as a critical paracrine SASP factor that phosphorylates EGFR and engages multiple key intercellular signaling molecules in recipient cancer cells (Figure S3j). The CM of DNA‐damaged PSC27 (PSC27‐BLEO) increased the proliferation by 2.4‐ to 3.2‐fold, migration by 2.3‐ to 3.5‐fold, and invasiveness by 2.5‐ to 3.4‐fold of PCa cell lines, respectively, while AREG clearance from PSC27 significantly reduced the capacity of stromal CM in enhancing the malignant phenotypes of PCa cell lines, with a reduction of 30%–36% (Figure 3d–f). Further, AREG depletion in stromal cells affected resistance of PCa cells to cytotoxic agents such as MIT, a capacity conferred by the full spectrum of SASP developed in PSC27 (Figure S3k).

Recently, we disclosed the remarkable potential of a damaged TME in conferring resistance to cancer cells that survive anticancer therapies, as exemplified by the SASP factors including WNT16B and SFRP2 (Sun et al., 2012, 2016). However, whether AREG plays a role pathologically comparable to these factors in treatment‐damaged TME remains unknown. To confirm AREG as a critical effector of the SASP, we applied cetuximab, a Food and Drug Administration (FDA)‐approved EGFR‐targeting monoclonal antibody, to treat PCa cells alongside the CM of PSC27‐BLEO. We found cetuximab substantially deprived the ability of damaged PSC27‐derived CM in conferring resistance on PCa cells, with an efficacy similar to that of the small molecule inhibitor AG1478 (Figure 3g). As the target of both cetuximab and AG1478 is EGFR, a receptor physically recognized and interacted by AREG, it remains unclear whether pharmaceutically targeting AREG with a target‐specific antibody is more effective in minimizing the acquired resistance of cancer cells. A significantly decreased cellular viability of PCa cells was observed when AREG mAb was supplemented with PSC27‐BLEO CM, with the effect comparable to or even higher than that of either AG1478 or cetuximab (*p* < .01 for most lines; Figure 3g). Interestingly, when AREG mAb and cetuximab were co‐applied to cell culture, the effect generally resembled that of AREG mAb alone (Figure 3g), suggesting addition of cetuximab to AREG mAb did not provide extra benefit. Although PSC27‐BLEO CM increased the viability of PC3 cells exposed to MIT at 0.1 ~ 1.0 μM, a range of dose that was designed to resemble the serum concentrations of this clinical agent in cancer patients, antibody‐mediated AREG depletion markedly compromised stroma‐conferred cancer resistance with a result close to the condition when AREG mAb was combined with cetuximab, as evidenced by the remarkable shift of both PCa and BCa cell survival curves (*p* < .01; Figure 3h; Figure S3l). Together, our data consistently suggested that either controlling EGFR as a plasma membrane receptor on recipient cells or targeting AREG as a soluble factor from damaged stromal cells can significantly deprive cancer cells of acquired resistance to chemotherapeutic agents.

### 2.4 AREG reprograms the transcriptomics of cancer cells and alters their phenotypes

**Figure 4:**
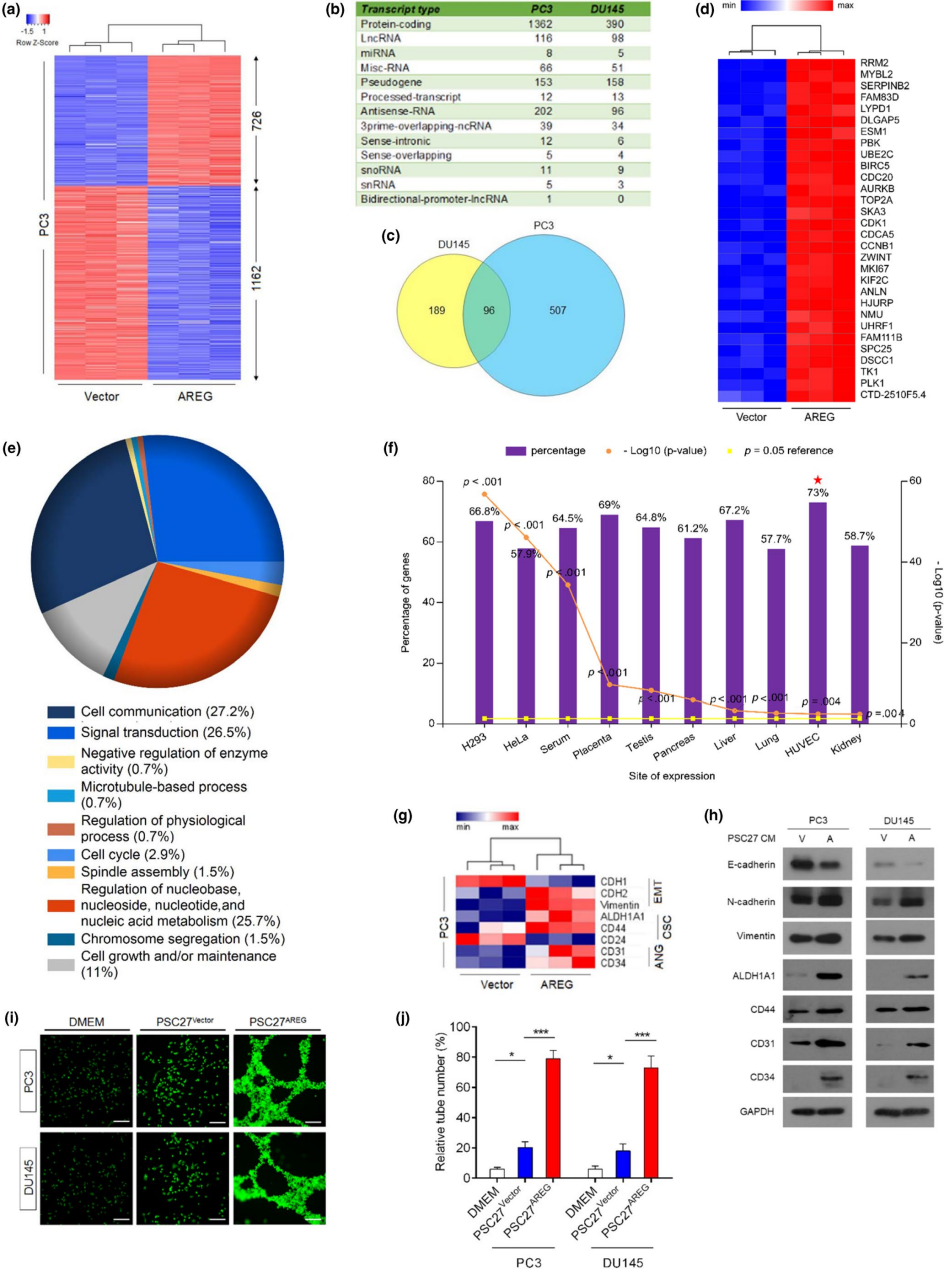
Paracrine AREG modifies cancer cell transcriptomics and induces phenotypic reprogramming. (a) Heatmap depicting differentially expressed human transcripts in PC3 cells after a 3‐day culture with AREG^+^ CM. In contrast to cancer cells cultured with control CM (vector), 726 and 1,162 genes were upregulated and downregulated, respectively, in those treated with the CM from AREG‐expressing PSC27 cells (AREG). (b) Statistics of transcripts differentially expressed (fold change either ≥2 or ≤0.5, with *p* < .05) in PC3 and DU145 cells upon AREG stimulation, and classified into typical categories according to functional annotations mapped by Gencode (V27). (c) Venn diagram indicating the overlap of 96 transcripts upregulated in PC3 and DU145 cells upon treatment with AREG‐containing CM from stromal cells (603/680 and 285/499 genes with unique annotations for PC3 and DU145, respectively). (d) Heatmap showing the top 30 upregulated transcripts by AREG in PC3 cells, sorted according to their expression fold changes in these cells. (e)Pie chart displaying the biological processes (BPs) that are most pronouncedly associated with transcripts upregulated by AREG, as revealed by GO analysis of the top transcripts in PC3 line. (f) Column chart depicting the expression sites of 726 transcripts upregulated in PC3 cells after AREG stimulation, with percentage and log10 (*P* value) per specific site indicated on the left and right Y‐axis, respectively. Data derived from by the FunRich program. Red star, human umbilical vein endothelial cell (HUVEC). (g) Heatmap of gene expression signatures associated with phenotypic changes including epithelial‐to‐mesenchymal transition (EMT)/cancer stem cell (CSC)/angiogenesis (ANG) after AREG stimulation of PC3 cells. Data were acquired from qRT–PCR assays. (h) Immunoblot assessment of protein‐level expression of phenotype‐associated markers displayed in (g). GAPDH, loading control. (i) Representative immunofluorescence images for morphological changes observed in PC3 and DU145 cells, upon in vitro culture for 3 days with AREG‐containing CM from PSC27 cells. PCa cells were then placed on the top of polymerized Matrigel in 12‐well plates for 10 hr, and tubular structures were photographed with fluorescence microscopy. Scale bars = 100 μm. (j) Statistics of tube formation observed for PCa cells upon treatment as described in (i). Data are shown as the percentage of high‐power fields (HPFs). Data of g–j are representative of three independent experiments, with three technical replicates performed per cell‐based assay (*p < .05 and ***p < .001)

Given the remarkable changes in cancer cell phenotypes caused by paracrine AREG, we next sought to dissect the influence of AREG on cancer cell expression pattern. We first chose to perform transcriptome‐wide sequencing (RNA‐Seq) to quantitate gene expression changes and profile the transcriptomics after treatment of PCa cells with stromal AREG. Bioinformatics analysis indicated that 1888 transcripts were upregulated or downregulated significantly (≥2‐fold, *p* < .05) in PC3 cells by paracrine AREG (Figure 4a), while the expression of 838 transcripts was changed in AREG‐affected DU145 cells (Figure S4a). Although the vast majority of these transcripts were protein‐coding (1,362 and 390 for PC3 and DU145, respectively), there were also molecules that fall into subcategories such as long noncoding RNAs (lncRNAs), microRNAs (miRNAs), miscellaneous RNAs (misc‐RNAs), pseudogenes, processed transcripts, antisense RNAs, and 3 prime overlapping ncRNAs (Figure 4b).

After mapping the transcripts to a gene ontology (GO) database comprising HPRD, Entrez Gene, and UniProt accession identifiers (Keshava Prasad et al., 2009; Maglott, Ostell, Pruitt, & Tatusova, 2011; UniProt, 2010), followed by the analysis of transcripts that filtered through a more stringent threshold of fourfold upregulation with available human genome annotations in PCa cells (603/680 and 285/499 for PC3 and DU145, respectively), we found an overlap class of 96 transcripts (Figure 4c; Table S5 for the top list), although their individual expression fold change and hierarchical order in these cell lines differ. Of note, multiple genes hitherto known to be associated with prostate cancer progression were observed in the top list of upregulated entities including but not limited to MYBL2, ESM1, PBK, and UBE2C in PC3, and MMP1, CCL5, and STC1 in DU145 (Figure 4d; Figure S4b). To gain further biological insights into the AREG‐induced expression tendency, we investigated the datasets with GO programs by focusing on the top 30 transcripts per line. Surprisingly, the biological processes led by these transcripts in both PC3 and DU145 cells were remarkably modified, showing a distinct pattern characterized by alterations in cell communication, signal transduction, nucleic acid metabolism, cell cycle, and immune response (Figure 4e; Figure S4c). Interestingly, when mapping the site of expression of PC3 and DU145 transcripts with a fold change ≥2 (*p* < .05), we observed prominent expression of genes linked to human endothelial cells (represented by the HUVEC), a phenomenon accompanied by the systemic expression change in genes correlated with epithelial‐to‐mesenchymal transition (EMT), cancer stem cell (CSC), and angiogenesis (ANG) development (Figure 4f,g; Figure S4d,e). Thus, in addition to the profound gene expression pattern change, AREG induced a striking epithelial‐to‐endothelial transition (hereby termed EET), suggesting a salient capacity of AREG in reprogramming the transcriptomics of cancer cells in the context of treatment‐remodeled TME.

As supporting evidence, immunoblots suggested modified expression of the markers intimately associated with EMT, CSC, and ANG, including E‐cadherin, N‐cadherin, vimentin, ALDH1A1, CD44, CD31, and CD34 (Figure 4h). Further, expression change of each molecule observed in cancer cells can be completely reset to their individual baseline upon elimination of AREG from stromal cells, as exemplified by the typical EMT markers (Figure S4f).

In line with the expression pattern shaped by paracrine AREG, we noticed emergence of robust tubule‐like structures when cancer cells were exposed to the AREG^+^ stromal cell CM on a specialized calcein‐incorporated basement membrane matrix, as revealed by either phase‐contrast or immunofluorescence microscopic imaging (Figure 4i and Figure S4g). Statistical appraisal suggested significantly enhanced capacity of both PC3 and DU 145 cells in forming capillary tube networks in the presence of stromal AREG (Figure 4j).

### 2.5 Therapeutically targeting AREG promotes tumor regression and prevents chemoresistance in vivo

Given the effects of paracrine AREG on cancer cell properties in vitro, we next interrogated whether stromal AREG causes any pathological consequences in vivo. First, we built tissue recombinants by admixing PSC27 sublines with PC3 cells at a preoptimized ratio of 1:4 before subcutaneously injecting them to the hind flank of experimental mice with nonobese diabetes and severe combined immunodeficiency (NOD/SCID). Animals were measured for tumor size at the end of an 8‐week period. Compared with tumors comprising PC3 and PSC27^Vector^, xenografts composed of PC3 and PSC27^AREG^ exhibited significantly enhanced volume (92.8%, *p* < .0001; Figure S5a). Conversely, knockdown of AREG from PSC27^AREG^ cells prior to tumor implantation resulted in considerably reduced tumor volumes (39.5% and 42.5% for shRNA^AREG^#1 and shRNA^AREG^#2, respectively, *p* < .0001 for both).

**Figure 5:**
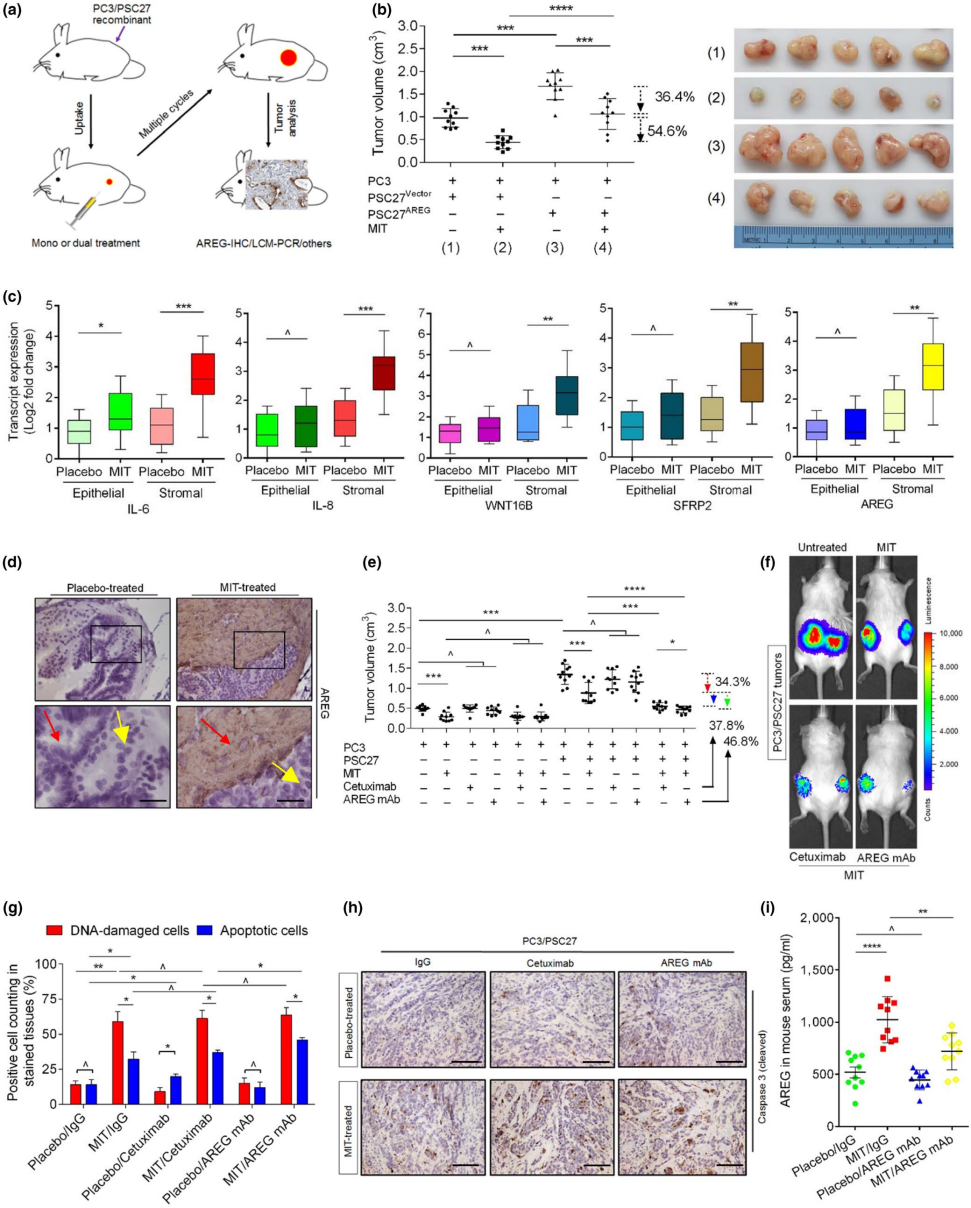
Therapeutically targeting AREG in the damaged TME promotes tumor responses. (a) Experimental diagram for nonobese diabetes and severe combined immunodeficient (NOD/SCID) mice. Two weeks after subcutaneous implantation and in vivo uptake of tissue recombinants, animals received either single (mono) or combinational (dual) agents administered as metronomic treatments composed of several cycles. (b) Statistical profiling of tumor end volumes. PC3 cells were xenografted alone or together with PSC27 cells to the hind flank of NOD/SCID mice. Prior to implantation, PSC27 cells were transduced with the control vector or AREG construct to make stable sublines. The chemotherapeutic drug MIT was administered to induce tumor regression. (c) Transcript assessment of several canonical SASP factors expressed in stromal cells isolated from the tumors of NOD/SCID mice. Tissues from animals implanted with both stromal and cancer cells in tumor grafts were subject to LCM isolation, total RNA preparation, and expression assays. (d) Representative IHC images of AREG expression in tissues isolated from placebo‐ or MIT‐treated animals. Square regions in the upper images were zoomed into lower images. Red arrows indicate stroma, and yellow arrows indicate cancer epithelium. Scale bars = 50 μm. (e) Statistical comparison of tumor growth in animals that underwent several different treatment modalities. Mice received PC3 cells implanted alone or combined with PSC27 cells before treatment by the chemotherapeutic drug (MIT) or combinational agents (MIT/cetuximab or MIT/AREG mAb). Tumor volumes were measured at the end of an 8‐week preclinical regimen. (f) Representative bioluminescence imaging (BLI) of PC3/PSC27 tumor‐bearing animals in the preclinical trial. Digital signals were proportional to in vivo luciferase activities measured by an IVIS device. (g) Statistical assessment of DNA‐damaged and apoptotic cells in the biospecimens analyzed in (e). Values are presented as percentage of cells positively stained by IHC with antibodies against γ‐H2AX or caspase 3 (cleaved). (h) Representative IHC images of caspase 3 (cleaved) in tumors at the end of therapeutic regimes. Biopsies of placebo‐treated animals served as negative controls for MIT‐treated mice. Scale bars = 100 μm. (i) Serum AREG concentration assessment of experimental mice treated by chemotherapy and/or AREG mAb. Data were derived from human AREG‐specific ELISAs. Data are representative of three independent experiments. *P* values were calculated by Student's *t* test (b, c, e, g, i) (^*p* > .05; *p < .05; **p < .01; ***p < .001; and ****p < .0001)

To closely mimic clinical conditions, we experimentally designed a preclinical regimen that incorporates genotoxic therapeutics and/or AREG/EGFR inhibitors (Figure 5a; Figure S5b). Two weeks after implantation when stable uptake of tumors in vivo was observed, a single dose of MIT or placebo was delivered to animals at the first day of 3rd, 5th, and 7th week until end of the 8‐week regimen. Contrasting to placebo‐treated group, MIT administration caused remarkably delayed tumor growth regardless of stromal production of AREG, validating the efficacy of MIT as a cytotoxic agent (54.6% for tumors comprising PSC27^Vector^ and 36.4% for those carrying PSC27^AREG^, respectively, *p* < .001 for both; Figure 5b). However, we noticed significantly enhanced expression of SASP factors including IL‐6, IL‐8, WNT16B, SFRP2, ANGPTL4, and MMPs, accompanied by the appearance of senescence markers such as p16^INK4a^ and SA‐β‐Gal in xenograft tissues comprising PC3/PSC27^Vector^ cells, suggesting the development of an in vivo cellular senescence and typical SASP triggered by genotoxicity (Figure 5c; Figure S5c,d). Interestingly, some of the SASP factors such as IL‐6 and MMP10, together with the typical senescence markers including p16^INK4a^, were co‐expressed in stromal and cancer epithelial cells, suggesting drug treatment induced comprehensive in vivo cellular senescence, although the SASP profile seemed to develop differently between stromal and cancer cells (Figure 5c; Figure S5c). Specifically, IHC staining revealed pronounced AREG induction in the MIT‐treated xenografts, with signals predominantly arising from the stroma (Figure 5d).

Next, we asked whether therapeutically eliminating AREG from the full SASP spectrum of damaged stoma would further enhance the therapeutic response of tumors. To this end, either cetuximab or AREG mAb was administered alongside MIT since the first dose of preclinical administration. Although MIT treatment caused prominent shrinkage of tumors composed of PC3 cells thoroughly (40.5%), administration of therapeutic antibodies did not show any effect (*p* > .05; Figure 5e). Interestingly, the antibodies did not provide further benefits even when used together with MIT, implying that PC3 tumors grow in a largely EGF/EGFR axis‐independent manner in the absence of surrounding stromal cells. Upon implantation of PC3 cells together with their stromal counterparts, tumor volumes significantly increased (170.1%, *p* < .001; Figure 5e), substantiating the tumor‐promoting effect of stromal cells in vivo. However, when animals carrying PC3/PSC27 tumors were treated with MIT, tumor volumes decreased significantly (34.3%, *p* < .001). Of note, when either cetuximab or AREG mAb was co‐administered with MIT as dual agents, tumor showed further reduction in end volume (37.8% and 46.8%, respectively; Figure 5e). Alternatively, bioluminescence imaging (BLI) of xenografts generated with cancer cells stably expressing luciferase (PC3‐luc) and stromal cells excluded the potential metastasis of cancer cells from the primary sites, with signals essentially supporting tumor growth patterns we observed in PC3/PSC27 animals (Figure 5f). The data suggest that classic chemotherapy combined with a TME‐targeting agent can induce tumor responses more dramatically than chemotherapy alone, and the efficacy of an AREG‐specific monoclonal antibody is even superior to cetuximab, an anti‐EGFR antibody widely applied to restrain EGFR^+^ neoplastic cell expansion by promoting their apoptosis in clinical patients (Mancini et al., 2017).

To investigate the mechanism directly responsible for AREG‐induced cancer resistance, we dissected tumors from animals treated by different agents 7 days after treatment, a time point prior to the development of resistant colonies. In contrast to the placebo, MIT administration caused dramatically increased DNA damage and apoptosis. Although cetuximab alone did not induce typical DDR, PC3 tumors displayed enhanced cell death, presumably due to the competent binding affinity of cetuximab to EGFR, a property that minimizes cancer survival (Figure 5g). However, when combined with MIT, cetuximab did not exhibit prominent efficacy in enhancing cell apoptosis, implying a reduced cytotoxicity when administered with MIT in these animals. In contrast to cetuximab, however, AREG mAb generated significantly, albeit slightly more apoptotic cells in tumor xenografts (Figure 5g). There was elevated caspase 3 cleavage, a typical hallmark of cell apoptosis, when AREG mAb was administered (Figure 5h). Of note, MIT‐mediated chemotherapy enhanced circulating AREG level in the plasma, which was substantially reduced when AREG mAb was used as a therapeutic antibody (Figure 5i). We further assessed the expression of angiogenesis‐associated markers including CD31 and CD34 in LCM‐isolated cancer cells from tumor xenografts, and found both factors significantly upregulated when animals were subject to chemotherapy (Figure S5e). However, upon combination of MIT treatment with AREG mAb administration, upregulation of CD31 and CD34 was substantial reversed, implying an angiogenesis‐promoting capacity of AREG under these in vivo conditions. The competency of AREG mAb‐caused reversion of chemotherapy‐elicited changes in cancer cells was further supported by similar changes in several EMT‐specific markers (Figure S5e), data consistent with our findings from cancer cell‐based in vitro assays (Figure 4g,h; Figure S4e,f).

### 2.6 Stromal AREG induces PD‐L1 expression in tumors and is an optimal TME target to enhance immunotherapeutic index

**Figure 6:**
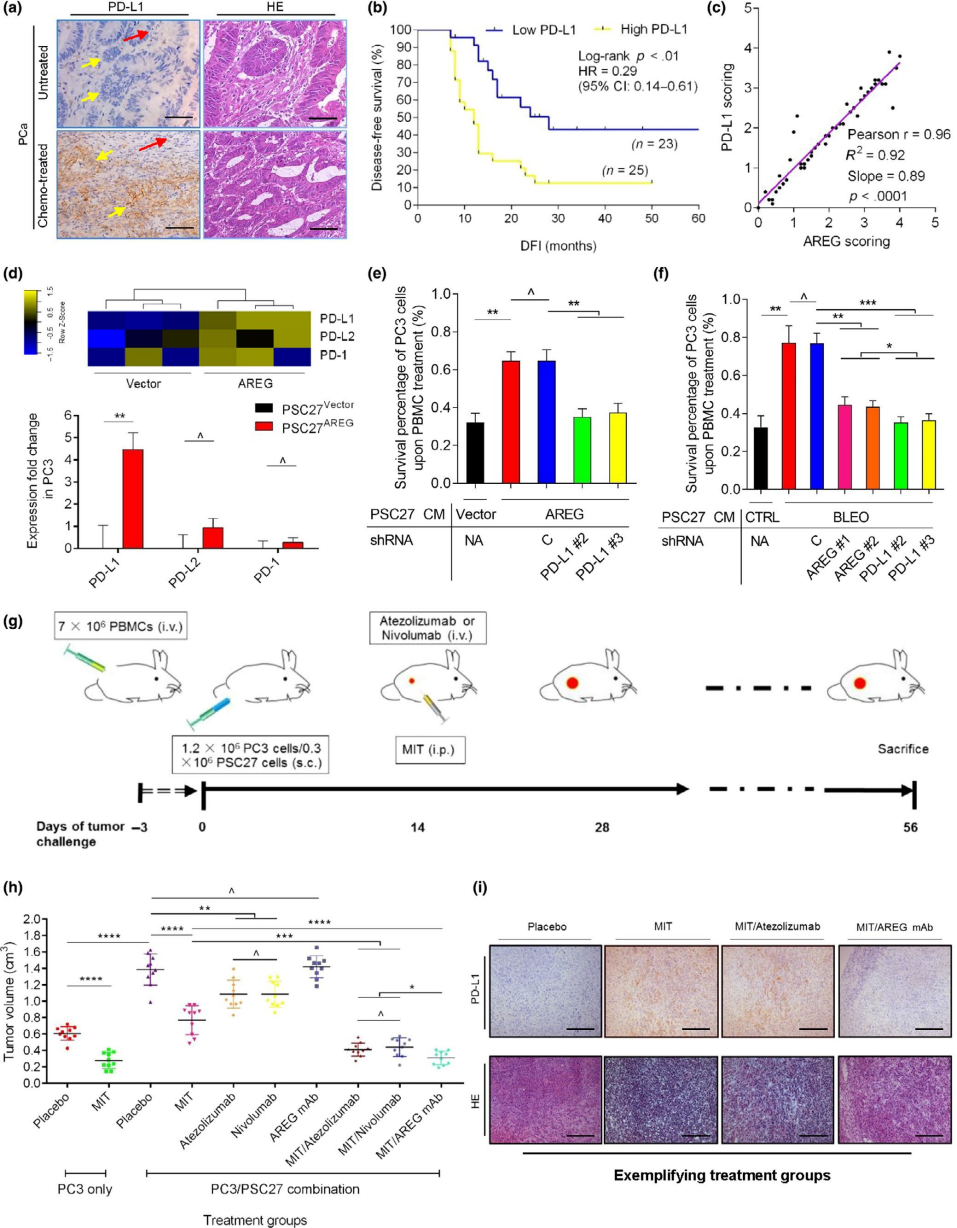
Stromal AREG induces PD‐L1 expression in tumors and is an exploitable target to enhance immunotherapeutic sensitivity. (a) Pathological assessment of primary tumors of PCa patients before and after chemotherapy. Left, IHC image of PD‐L1 staining. Right, HE staining. Top, untreated. Bottom, chemo‐treated. Red arrows indicate stroma, and yellow arrows indicate cancer epithelium. Scale bars = 100 μm. (b) Kaplan–Meier profiling of PCa patient survival. Disease‐free survival (DFS) stratified according to PD‐L1 expression (low, average score <2, blue line, *n* = 23; high, average score ≥2, yellow, *n* = 25). DFS represents the length (months) of period calculated from the date of PCa diagnosis to the point of first‐time disease relapse. Survival curves generated according to the Kaplan–Meier method, with *P* value calculated using a log‐rank (Mantel–Cox) test. (c) Statistical correlation between AREG and PD‐L1 pathological scores (Pearson's analysis, *r* = .96, *p* < .0001) in the 48 tumors with matching protein expression data. (d) Comparative analysis of PD‐L1/PD‐L2/PD‐1 expression in PC3 cells cultured with CM of control (vector) or AREG‐overexpressing (AREG) PSC27 cells. Top, expression profile generated from RNA‐Seq data. Bottom, qRT–PCR analysis of above gene expression in PC3 cells. (e) Survival evaluation of PC3 cells upon 3‐day culture with either control (vector) or AREG‐containing (AREG) CM of PSC27 cells, in the presence of human peripheral blood mononuclear cells (PBMCs). PC3 cells were lentivirally infected with scramble (C) or PD‐L1‐specific shRNAs (#2, #3) to make sublines prior to in vitro treatment. (f) PC3 sublines described above were subject to culture with CM of treatment‐naive (CTRL) or bleomycin‐damaged (BLEO) PSC27 cells, while human PBMCs were applied. Results were evaluated as the percentage of PC3 cells that survived 3 days of continuous culture. PC3 cells lentivirally infected with AREG‐specific shRNAs (#1, #2) were examined as parallel controls. (g) Illustrative diagram of the preclinical trial involving a humanized mouse model. To assess the anticancer properties of immune‐stimulatory monoclonal antibodies (atezolizumab, anti‐PD‐L1; nivolumab, anti‐PD‐1; and AREG mAg, anti‐AREG), Rag2^−/−^IL2Rγ^null^ mice with T, B, and NK lymphocyte deficiency were intravenously (i.v.) injected with 7 × 10^6^ human PBMCs 3 days before subcutaneous (s.c.) implantation of 1.2 × 10^6^ PC3 cells with or without 0.3 × 10^6^ PSC27 cells to the hind flank. Two weeks after tumor xenografting, therapeutic antibodies (i.v.) were provided alone or together with MIT (i.p), with the treatment performed once every other week for three cycles. At the end of the 8‐week regimen, animals were sacrificed, with tumors collected for pathological appraisal. (h) Statistical comparison of end volumes of tumors grown in Rag2^−/−^IL2Rγ^null^ animals that experienced different treatment modalities as described above. Mice received PC3 cells implanted alone were assessed as counterpart control to those xenografted with PC3/PSC27 recombinants. Tumor volumes were measured at the end of the 8‐week preclinical regimen. (i) Pathological analysis of humanized animals. Top, IHC images derived from IHC staining against PD‐L1. Bottom, HE images. Tumor tissues from placebo‐, MIT‐, MIT/atezolizumab‐, and MIT/AREG mAb‐treated mice are displayed as exemplifying samples. Data are representative of three independent experiments. *p* values were calculated by Student's *t* test (e, f, h) (^*p* > .05; *p < .05; **p < .01; ***p < .001; and ****p < .0001)

The efficacy of conventional anticancer drugs not only involves direct cytotoxic/cytostatic effects, but also relies on the (re)activation of tumor‐targeting immune activities (Galluzzi, Buque, Kepp, Zitvogel, & Kroemer, 2015). We thus asked whether the treatment‐damaged TME alters the response of cancer cells to immunotherapeutic agents. Our expression database suggested that PD‐L1 (also CD274, B7‐H1) was substantially upregulated in PCa cells after exposure to PSC27^AREG^ CM (Table S5), consistent with the pathological data which demonstrated remarkable expression of PD‐L1 in primary tissues of post‐treatment PCa patients (Figure 6a). Expression level of PD‐L1 in tumor foci is significantly correlated with poor disease‐free survival in post‐treatment period (*p* < .01, log‐rank test; Figure 6b). We further noticed a prominent linear correlation between AREG expression in the stroma and PD‐L1 expression in the primary tumor (*p* < .0001; Figure 6c).

Although PD‐L1 is subject to induction by AREG, PD‐L2 and PD‐1 remain largely unchanged, a fact supported by the data from both RNA‐Seq and qRT–PCR (Figure 6d; Figure S6a). The findings were largely reproduced by immunoblot assay of these molecules in cancer cells (Figure S6b). Further, we noticed that expression level of PD‐1, the receptor expressed on plasma membrane of immune cells including cytotoxic T lymphocytes (CTLs) and specifically interacted by PD‐L1/PD‐L2, remained unchanged in human peripheral blood mononuclear cells (PBMCs) upon exposure to AREG^+^ stromal CM (Figure S6b).

EGFR‐mediated Akt activation is associated with PD‐L1 expression, which can be reduced by EGFR inhibitors in cancer cell lines carrying activated EGFR (Akbay et al., 2013). We asked whether PD‐L1 upregulation by paracrine AREG is subject to intracellular signaling that involves EGFR, its downstream factors, or other molecules in recipient cancer cells. To address this, a group of small molecule inhibitors including those targeting EGFR (erlotinib, AG1478), PI3K (LY294002), Akt (MK2206), mTOR (rapamycin), Mek1/2 (PD0325901), NF‐κB (Bay 11–7082), Jak1/2 (ruxolitinib), and p38 (SB203580) was individually applied to treat PCa cells together with stromal AREG. Interestingly, almost all of these inhibitors markedly dampened PD‐L1 production even in the presence of paracrine AREG, although suppression of p38 failed to cause PD‐L1 reduction (Figure S6c). Together, our data evidently showed the regulation of AREG‐induced PD‐L1 synthesis in PCa cells, a process that functionally involves EGFR and its downstream elements including but not limited to PI3K, Akt, mTOR, Mek1/2, Jak1/2, and NF‐κB, while p38, one of the major cellular stress sensors, did not seem to be engaged.

Next, we investigated the influence of AREG‐induced PD‐L1 expression on the immune activity of human CTLs against cancer cells, by employing PBMCs freshly collected from human patients and monitoring their efficacy in targeting PCa cells. In the presence of stromal AREG, survival of cancer cells was remarkably enhanced even when PBMCs were added to culture. However, the advantage was minimized upon PD‐L1 elimination from PCa cells (Figure 6e; Figure S6d,e). We further observed elevated survival of cancer cells when they were exposed to the CM from PSC27 predamaged by BLEO, which was reversed upon PD‐L1 depletion in cancer cells (Figure 6f; Figure S6f). Of note, PCa cell survival decreased when AREG was eliminated from PSC27, although the reduction extent in the presence of activated PBMCs was slightly but significantly less than that caused by PD‐L1 depletion from PCa cells (Figure 6f; Figure S6f). The data strongly suggest that stromal AREG‐mediated PD‐L1 expression in cancer cells represents a major force of resistance to immunosurveillance, a response triggered by the damaged stroma but indeed exploitable to enhance the sensitivity of tumors to immunotherapeutic agents.

We next explored the feasibility and efficacy of tumor treatment by combining chemotherapy and immunotherapy. Previous studies demonstrated that human lymphocytes transferred into immunodeficient mice can undergo activation and redistribution to murine organs, while administration of therapeutic antibodies including nivolumab is able to restrain tumor progression (Sanmamed et al., 2015). We hereby chose to use Rag2^−/−^IL2Rγ^null^ mice, which are devoid of T, B, and NK lymphocytes and permit to establish humanized animal models (Herndler‐Brandstetter et al., 2017). Human PBMCs were intravenously transplanted before establishment of subcutaneous tumor xenografts. Atezolizumab or nivolumab, each an FDA‐approved monoclonal anti‐PD‐L1/PL1 agent, was administered to Rag2^−/−^IL2Rγ^null^ mice after PC3/PSC27 implantation (Figure 6g). As a hallmark of activated CTLs is the production of cytokines, we examined animal plasma and observed increased human interferon‐γ (h‐IFN‐γ) and TNF‐α (h‐TNF‐α) levels in mice that received atezolizumab or nivolumab, but not MIT, control IgGs, or AREG mAb (Figure S6g,h). The data suggest that the PD‐L1/PD‐1 agents effectively activated transplanted PBMCs and induced substantial production of typical cytokines in vivo.

Preclinical results indicated that MIT significantly reduced the volumes of tumors composed of PC3 cells only, with an extent more dramatic than atezolizumab or nivolumab (Figure S7a). Although AREG mAb failed to generate any remarkable changes to tumor growth, combination of this antibody with MIT achieved prominent effects in abrogating tumor progression, with the efficiency approaching that manifested by combined use of MIT with a PD‐L1/PD‐1‐targeting antibody.

We next focused on the consequence of chemotherapy and/or immunotherapy on the development of tumors comprising cancer cells and their stromal counterparts. Contrasting to PC3 alone, co‐implantation of PC3 and PSC27 cells led to significantly higher tumor volume, consistent with the results we observed in NOD/SCID animals (Figure 6h). Although administration of either atezolizumab or nivolumab alone remarkably decreased tumor sizes, they were less effective than MIT treatment, suggesting the limited efficacy of targeting PD‐L1/PD‐1 in these animals. Of note, the reduction extent of tumor volumes achieved by MIT/AREG mAb was slightly but significantly higher than that caused by either MIT/atezolizumab or MIT/nivolumab, not only underscoring the superior potential of classic chemotherapy combined with anti‐PD‐L1/PD‐1 agents in functionally competent TME, but also proving that the modality can be alternatively reconstituted by replacing PD‐L1/PD‐1 antibodies with an AREG mAb (Figure 6h). After tumor histological dissection, we further found that delivery of MIT induced substantial expression of PD‐L1 in the tumor foci, a process markedly counteracted when MIT was co‐administered with AREG mAb but not a PD‐L1/PD‐1‐targeting agent such as atezolizumab (Figure 6i).

To validate the findings in a hormone‐native setting, we generated tumor xenografts with VCaP, a prostate cancer cell line that expresses androgen receptor (AR) and grows in an androgen‐sensitive manner (Kim, Watson, et al., 2018). Combination of VCaP and PSC27 presented results similar to those observed in PC3/PSC27 tumors (Figure S7b), suggesting that the efficacy of therapeutic agents is essentially hormone‐independent. To further expand, we performed studies with human breast cancer (BCa) xenografts consisting of MDA‐MB‐231, a malignant BCa cell line, and HBF1203, a breast stromal line. Preclinical assays showed that BCa data largely reproduced those obtained from PCa trials (Figure S7c).

To validate the safety and feasibility of the therapeutic regimens, we performed pathophysiological assays. Our data suggested that either single or combinational treatment was well tolerated, with mice maintaining normal body weight throughout the therapeutic regimen (Figure S7d). Together, these results suggest that combining conventional chemotherapy with an AREG‐ or PD‐L1/PD‐1‐targeting agent has the competency to enhance tumor response without causing severe in vivo cytotoxicity.

### 2.7 AREG is a novel biomarker imaging in vivo SASP and implies potential immunosuppression in a treatment‐damaged TME

**Figure 7:**
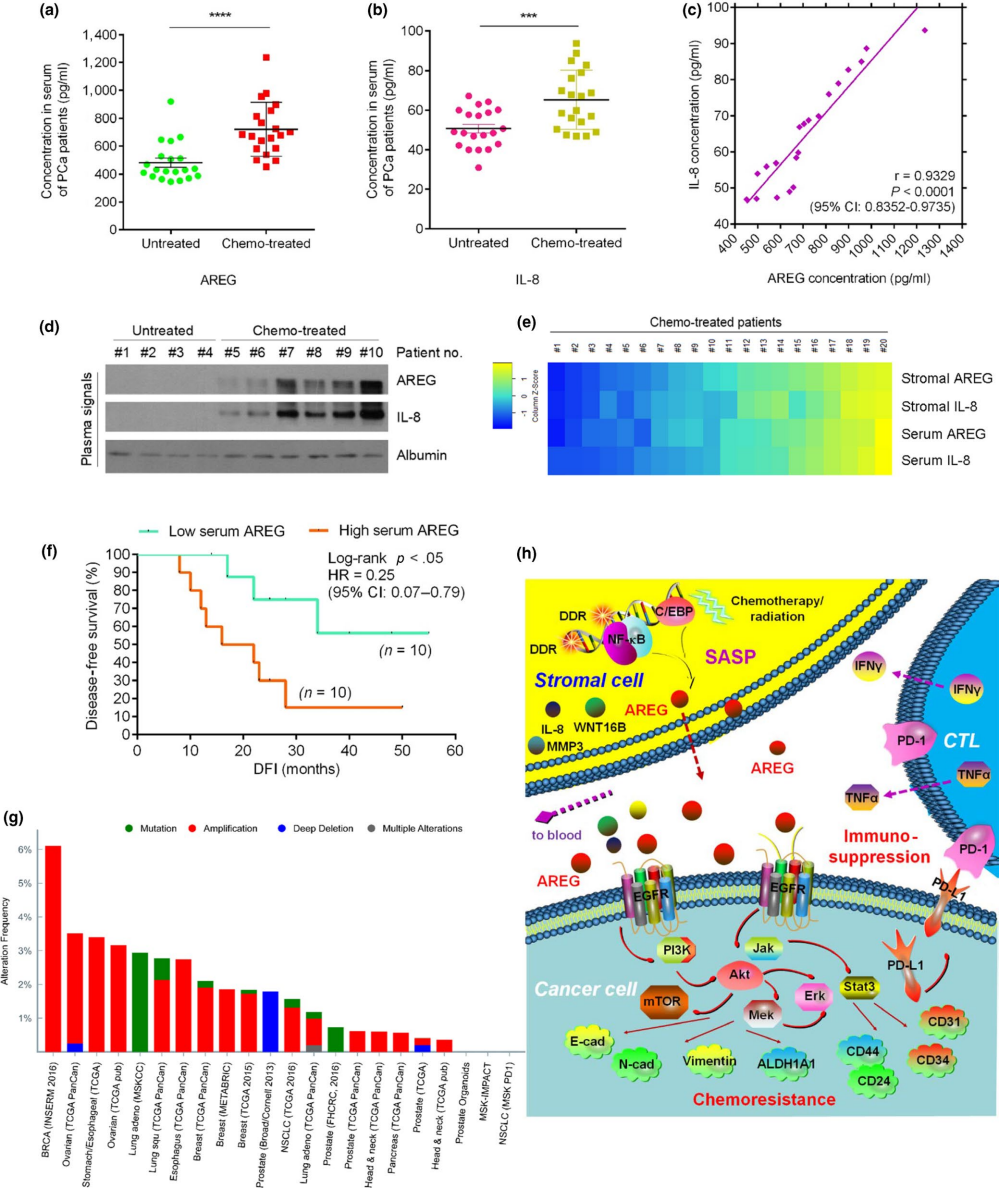
AREG is a novel circulating biomarker of in vivo SASP, implies immunosuppression, and predicts adverse therapeutic outcome. (a) Abundance of AREG protein in the serum of untreated and chemo‐treated PCa patients. Data were derived from ELISA measurement and shown as mean ± *SD*, *n* = 20. (b) Abundance of IL‐8 protein in patient serum analyzed in (a). Data were derived from ELISA measurement and presented as mean ± *SD*, *n* = 20. (c) Scatterplot showing correlation between AREG and IL‐8 in the serum of individual patients studied in (a) and (b). Pearson's correlation coefficient, *P* value, and confidence interval indicated. (d) Immunoblot of AREG and IL‐8 in the serum of randomly selected PCa patients from untreated and chemo‐treated groups, respectively (*n* = 4 for untreated; *n* = 6 for treated). Albumin, sample loading control for patient serum protein. (e) Heatmap depicting the overall correlation between stromal AREG, stromal IL‐8, serum AREG, and serum IL‐8 in chemo‐treated patients (*n* = 20). The raw scores of stromal AREG and IL‐8 were derived from independent pathological reading of primary PCa tissues, while scores of serum AREG and IL‐8 came from ELISAs. Color key, relative expression of these two factors in stromal tissue or patient serum. (f) Kaplan–Meier survival analysis of chemo‐treated PCa patients. Disease‐free survival (DFS) stratified according to AREG expression in tumor stroma (low, average score <2, turquoise line; high, average score ≥2, orange line). DFS represents the length (months) of period calculated from the date of chemotherapy completion to the point of first‐time disease relapse. Survival curves generated according to the Kaplan–Meier method, with *p* value calculated using a log‐rank (Mantel–Cox) test. *n* = 10 per group. (g) Multidimensional TCGA data (bar graph) show alterations of AREG across an array of human cancer types at genomic level, including mutation, amplification, deep deletion, and multiple alterations. Modification frequency is displayed in percentage. Data were extracted from TCGA database followed by deep analysis with cBioPortal online analyzing tools. (h) Work model of AREG induction in the treatment‐damaged TME, pathological impact on intercellular signaling network of cancer cells, immunosuppressive consequence by activation of immune checkpoint against CTL/NK cells, and the potential as a novel circulating biomarker for clinical surveillance/prognosis. Data of a–d are representative of three independent experiments. *P* values were calculated by Student's *t* test (a, b), Pearson's analysis (c), and log‐rank test (f) (***p < .001 and ****p < .0001)

We next sought to assess whether AREG is experimentally detectable in the circulating system of cancer patients post‐treatment. To this end, we collected peripheral blood from PCa patients, including one cohort that experienced chemotherapy and the other that did not. Upon serum analysis of chemo‐treated patients by antigen‐specific ELISA, we found AREG plasma level in the treated cohort significantly higher than that of the treatment‐naïve group (Figure 7a). Notably, the tendency was phenocopied by IL‐8, a canonical hallmark factor of the SASP (Figure 7b). The data suggested development of an in vivo SASP, the index of which can be technically measured by quantifying the concomitantly expressed SASP factors including but not limited to AREG and IL‐8 in the peripheral blood of post‐treatment cancer patients.

We interrogated whether the amount of circulating AREG is indeed correlated with that of other typical SASP factors such as IL‐8 in a same individual patient after treatment. Upon evaluation by ELISA, we noticed a significant and positive correlation between AREG and IL‐8 (Figure 7c). Further, immunoblots not only confirmed enhanced AREG and IL‐8 levels in the serum of chemo‐treated PCa patients rather than those untreated, but also displayed simultaneously occurring changes in these two factors, thus validating their correlation at serum level (Figure 7d). Additional datasets were obtained from the cohorts of BCa, an alternative solid tumor type, thus providing an extra set of evidence supporting increased AREG/IL‐8 expression and their significant correlation in the peripheral system of post‐treatment populations (Figure S8a–c).

To gain further insights, we performed longitudinal investigation in the primary tumor tissue and circulating blood of the post‐treatment cohort (20 patients). Strikingly, cross‐organ analysis indicated a remarkable association between the tissue expression and serum level of each factor, with the amounts of AREG and IL‐8 seemingly in parallel for each individual patient (Figure 7e). To establish the appropriateness and reliability of employing AREG/IL‐8 for in vivo SASP measurement, we selectively acquired stromal cells from the primary tissues of PCa patients via LCM, and analyzed the expression of a subset of signature SASP factors, including but not limited to MMP1, CXCL3, IL‐1β, WNT16B, IL‐6, and GM‐CSF (Figure S8d). Interestingly, expression of the vast majority of these factors in stroma consistently paralleled the alteration of both AREG and IL‐8 in the same tissue, with patient samples organized from low to high per factor fold change. However, not surprisingly, several non‐SASP factors such as IL‐2/3/5/12/17 failed to show such a distinct correlation (Figure S8d). Thus, our data suggest that AREG represents one of the major TME‐derived soluble factors and precisely reflects the development of an in vivo SASP, and thus can be exploited to assess the magnitude of the SASP in cancer patients after clinical therapy. Further, we observed a significant and negative correlation between AREG serum index and patient survival in the post‐treatment PCa cohort, which resembled the data from the post‐treatment BCa patients (Figure 7f; Figure S8e). Similar to the PCa clinical data (Figure 6c), expression level of AREG in BCa stroma appears to be intimately correlated with that of PD‐L1 in their adjacent tumor foci (Figure S8f), further substantiating the potential of AREG to be exploited as a molecular indicator for immune checkpoint activation in a functionally active TME. Although AREG is subject to frequent mutation, amplification, deep deletion, and even multiple alternations in cancer patients as revealed by the TCGA genomics data (Figure 7g), routine surveillance of AREG through a noninvasive strategy such as liquid biopsy provides a novel, precise, and straightforward avenue for both treatment efficacy assessment and prognosis of advanced malignancies in future clinical oncology.

## 3 DISCUSSION

Resistance to anticancer treatments is a major problem in both cancer research and clinical practice. The mechanisms of resistance to “classic” cytotoxic chemotherapy, radiation, and even targeted therapy share several features, including modifications of the drug target, activation of pro‐survival pathways, and disability of apoptosis machineries (Holohan, Schaeybroeck, Longley, & Johnston, 2013). With the mounting arsenal of anticancer agents and the advancement of high‐throughput screening techniques, there are now unprecedented opportunities to tackle cancer resistance via clinical assessment of rational therapeutic drug combinations and exploration of precise biomarkers to enable patient stratification. In this study, we uncovered that AREG, a soluble factor produced by the treatment‐damaged TME, confers pronounced resistance on surviving cancer cells and simultaneously creates an immunosuppressive microenvironment by engaging PD‐L1, a type I transmembrane protein that functionally activates the immune checkpoint (Figure 7h). Released as one of the hallmark SASP molecules, AREG is readily detectable in the circulating system of post‐treatment cancer patients and can be exploited as a novel and noninvasive biomarker for evaluation of therapeutic effects and clinical prognosis of patient outcome in cancer medicine.

Chemotherapy is one of the principal anticancer modalities. However, much like other type of treatments, the effectiveness of chemotherapeutics is limited by drug resistance, which can be divided into two broad categories: intrinsic and acquired. In contrast to intrinsic resistance, which re‐exists in cancer cells before initiation of treatments, acquired resistance arises in the course of therapies and is frequently supported by the tumor‐adjacent stroma (Junttila & de Sauvage, 2013). For instance, cancer resistance can be driven by a treatment‐damaged TME, which is pathologically fueled by the SASP (Sun et al., 2012, 2016; Zhang et al., 2018). Originally discovered as an essential biological feature of senescent cells, the SASP is now established as an important contributor in the development of multiple aging‐related complications, including atherosclerosis, osteoarthritis, physical frailty, and systemic inflammation (Childs et al., 2016; Demaria et al., 2017; Jeon et al., 2017; Zhu et al., 2015). However, increasing lines of evidence support that the SASP has broad implications in human cancers. Although highly context‐dependent, the SASP serves many consistent functions in the TME, including those involved in cancer initiation, growth, metastasis, and even relapse (Demaria et al., 2017). In contrast to studies that support the beneficial effects of TIS induction in cancer cells, including tumor growth stalling and the SASP‐mediated immune response that promotes elimination of senescent cancer cells, therapy‐triggered off‐target effects on the surrounding benign TME components can have undesirable outcome during and after cancer treatment, particularly when cancer cells develop acquired resistance against subsequent cycles of therapeutic intervention.

Synthesized as a membrane‐anchored precursor protein that can engage juxtacrine signaling on adjacent cells, AREG is a member of the EGF family (Berasain & Avila, 2014). The biological effect of AREG is mainly mediated through its binding and activation of EGFR (also ErbB1), a widely expressed transmembrane RTK (Avraham & Yarden, 2011). By comparison of multiple cell lines and anticancer treatments, we showed that stromal cells are more ready to express AREG than cancer cells upon treatment by DDA agents that cause typical DNA lesions, instead of NDDA treatments that do not target DNA, a feature that is indeed shared by most of the SASP factors (Zhang et al., 2018). Intriguingly, AREG can be alternatively delivered via extracellular vesicles such as exosomes in the treatment‐naïve settings of multiple myeloma, non‐small‐cell lung cancer, and chronic myelogenous leukemia, each case activating the EGFR pathway in recipient cells without chemotherapy or SASP engagement in the TME (Corrado et al., 2016; Raimondo et al., 2019; Taverna et al., 2017). Through in vitro assays, we revealed that AREG is one of the major effectors of the full SASP spectrum, as elimination of AREG from stromal cells caused substantially weakened cancer cell malignancies, including proliferation, migration, invasion, and more importantly chemoresistance. Of note, chemotherapy combined with an AREG‐targeting monoclonal antibody achieved an efficacy that was even higher than that generated by chemotherapy plus cetuximab, a FDA‐approved anti‐EGFR agent, suggesting that EGFR may not be the sole cell surface receptor interacted by paracrine AREG in the TME niche. Thus, future studies are warranted to address this interesting issue.

Although largely neglected in clinical practice, accumulating evidence suggests that the efficacy of conventional anticancer agents not only involves direct cytotoxic and/or cytostatic effects, but also relies on the (re)activation of tumor‐targeting immune responses. Many chemotherapeutics can presumably promote such responses by enhancing the immunogenicity of cancer cells or through inhibiting immunosuppressive circuitries established by developing tumors, as evidenced by the case of immunogenic chemotherapeutics that cause T‐cell infiltration and sensitize tumors to ICB (Pfirschke et al., 2016). Such immunological “side” effects of chemotherapy are indeed desirable, and a thorough comprehension will facilitate the design of novel combinatorial regimens with improved clinical outcome. The clinical activity of most, if not all, conventional anticancer agents currently licensed for use in human clinics can be attributed to the re‐establishment of immunosurveillance (Galluzzi et al., 2015). Although former studies indicated the production of AREG by special subsets of immune cells (Zaiss et al., 2015, 2013), we interrogated the immune regulation potential of AREG as a SASP factor released by the damaged TME, and explored the possibility of developing combinatorial regimens with improved therapeutic profile. Intriguingly, the data showed that beyond the phenotypic changes in cancer cells, such as appearance of EMT, and development of CSC and ANG, which together constitute a unique EET pattern, significant upregulation of PD‐L1 was observed upon co‐culture with stromal cells either showing a full‐blown SASP or producing exogenous AREG. More importantly, AREG seems to be the major factor of the full SASP spectrum in eliciting such immune checkpoint activation. Thus, in contrary to the well‐established concept of immunosurveillance enhanced by the SASP (Kang et al., 2011; Xue et al., 2007), our findings disclosed an immunosuppressive function of the SASP manifested by the treatment‐damaged TME. Such an effect can be mediated by certain factors such as AREG, which potently induces PD‐L1 expression in recipient cancer cells and causes exhaustion of CTL and NK cells, presumably overriding the net immunostimulatory capacity of senescent cells alone. We thus explored the feasibility of targeting this response by using therapeutic antibodies that specifically recognize either PD‐L1 or its receptor, PD‐1, agents that block the immune checkpoint. Our preclinical studies showed that such an ICB treatment was more effective when synergizing with conventional chemotherapy, which causes irreparable TME damage and induces substantial AREG expression in stromal compartments, resulting in the formation of an immunosuppressive niche via PD‐L1 upregulation in cancer cells. Surprisingly, in contrast to the ICB agents approved for clinical purposes, AREG mAb exhibited even higher therapeutic efficiency when combined with “classic” chemotherapeutics, a case observed in both PCa and BCa animal models humanized for ICB studies. It is reasonable to speculate that these effects are mediated simultaneously through minimizing chemoresistance and dampening immune checkpoint activation, both processes orchestrated by the paracrine AREG in an immunocompetent TME.

In clinical oncology, routine chemotherapy is compatible with an immune response, as experimental dendritic cell (DC)‐, DNA‐, or peptide‐based anticancer vaccines can elicit tumor‐targeting immune responses in patients treated with conventional chemotherapeutics (Bloy et al., 2014). Chemotherapy often interacts positively with ICB‐based immunotherapy. For instance, ipilimumab improves the efficacy of carboplatin/paclitaxel‐based chemotherapy in lung cancer, while oxaliplatin boosts anti‐PD‐L1 mAb therapy against colorectal cancer (Reck et al., 2013; Song et al., 2018). Specifically, PD‐L1 inhibitors, including a locally expressed PD‐L1 trap delivered by lipid–protamine–DNA nanoparticles, hold the potential to improve cancer treatment index following oxaliplatin‐based chemotherapy (Song et al., 2018). However, a recent study addressing the impact of chemotherapy on the PD‐1/PD‐L1 pathway revealed that 5‐fluorouracil/oxaliplatin (Folfox) induced complete and long‐term responses in colorectal cancer mice upon combination with anti‐PD‐1 treatment, while each monotherapy failed (Dosset et al., 2018). The study presented a link between Folfox‐triggered immunogenic cancer cell death, cancer cell PD‐L1 expression, and CD8^+^ T‐cell infiltration, raising the emerging concept that adaptive immune escape is a dominant mechanism of cancer resistance in the context of a pathologically activate TME (Ribas, 2015). Thus, recognizing the specific adaptive resistance mechanisms is likely to favor the personalized development of immunotherapies tailored to special conditions in cancer clinics. In our work, the SASP potently drives therapeutic resistance by not only enhancing the survival of cancer cells against chemotherapeutic agents but also consolidating the immune checkpoint potential against CTL and NK cell activities, a process that is mainly mediated by a soluble factor AREG.

ICB‐based immunotherapies can be extraordinarily effective, but so far have benefited only a minority of patients whose tumors are pre‐infiltrated by T cells, particularly when appropriately selected immunogenic drugs (e.g., oxaliplatin plus cyclophosphamide for treatment of tumors expressing oncogenic KRAS but lacking p53) are used (Pfirschke et al., 2016). Our study indicates that the proportion of cancer cases responding to anticancer treatments can be feasibly and substantially expanded by combining conventional chemotherapies with ICB treatment, an effective and promising strategy that sensitizes tumors to checkpoint inhibitory agents and controls cancer durably by antagonizing the survival advantage conferred by a treatment‐remodeled TME. However, there are also caveats in this study. First, for patients with EGFR mutations such as T790M in lung cancer, the efficacy of anti‐AREG may be restrained, as EGFR downstream signaling network remains active. Second, cancer cell‐autonomous AREG expression may occur in some tumor types, although not manifested by clinical samples (PCa and BCa) investigated by our study. Beyond cancer cell‐expressed AREG in special cases, the TME‐derived AREG exerts an extra layer of chronic and long‐term pathological impacts on patients, including but not limited to development of acquired resistance to anti‐EGFR therapies such as cetuximab. However, targeting stroma‐derived AREG represents an optimal therapeutic effort that is able to eliminate AREG produced by both stromal cells and cancer cells in the TME niche. Third, we used PBMCs from donors unrelated to the patient from which PC3 cells were originally derived. In such a case, it is reasonable to speculate ample alloreactivity based on the recognition of MHC alloantigens in transplanted tumors. Though this experimental setting is clearly not maximally mimicking the response against tumor‐associated neoantigens, our model permits a pharmacodynamic appraisal of in vivo effects of an immunostimulatory or a single SASP factor‐specific mAb, thus offering valuable mechanistic clues to be explored by future research pipelines.

Understanding the immunomodulatory effects of conventional anticancer drugs may have a profound effect on the design of novel and optimal treatment options that allow these agents to be combined with immunotherapies (Galluzzi et al., 2015). There are two cases of combinatorial regimens in which immunotherapy can markedly improve the clinical profile of anticancer treatments, particularly chemotherapy. In the first scenario, immunotherapy may be harnessed to maximize the immunostimulatory effects of therapeutic agents such as those reported previously (Dosset et al., 2018; Pfirschke et al., 2016). Second, immunotherapy may be used to neutralize the unwarranted immunosuppressive effects of anticancer drugs, particularly those mediated by the TME components as illustrated by our study. Each case, insightful understanding and successful harnessing of the currently approved anticancer drugs will permit to design more efficient and safer combinatorial therapies, and such a pragmatic strategy is believed to be able to present the best imaginable service to cancer patients in future practice.

## 4 EXPERIMENTAL PROCEDURES

### 4.1 Cell culture

Primary normal human prostate stromal cell line PSC27 and breast stromal cell line HBF1203 were maintained in stromal complete medium as described (Sun et al., 2012). Prostate cancer epithelial cell lines PC3, DU145, and LNCaP and breast cancer epithelial cell lines MCF7, MDA‐MB‐231, MDA‐MB‐468, T47D, and BT‐474 (ATCC) were routine cultured with RPMI 1640 (10% FBS). BPH1 was isolated from prostatic tissue with benign hyperplasia and immortalized by SV40‐LT (Hayward et al., 1995), while M12 was derived from BPH1 but phenotypically neoplastic and metastatic (Bae et al., 1998). All cell lines were routinely tested for mycoplasma contamination and authenticated with STR assays.

### 4.2 Stromal cell treatments

Stromal cells were grown until 80% confluent (CTRL) and treated with genotoxic agents 50 μg/ml bleomycin (BLEO), 500 nM mitoxantrone (MIT), 10 μM satraplatin (SAT), 10 μM doxorubicin (DOX), 100 μM cisplatin (CIS), 200 μM carboplatin (CARB), 0.6 mM hydrogen peroxide (HP), or γ‐radiation by a ^137^Cs source at 743 rad/min for 10 Gy (RAD). Alternatively, cells were treated by nongenotoxic chemicals paclitaxel (10 nM, PTX), docetaxel (10 nM, DTX), vincristine (20 nM, VCR), vinorelbine (20 nM, VNB), or vinblastine (20 nM, VBL). After treatment, the cells were rinsed briefly with PBS and allowed to stay for 7–10 days prior to performance of various examinations.

### 4.3 Cancer patient recruitment and biospecimen analysis

Chemotherapeutic administration involving genotoxic agents was performed for primary prostate cancer patients (clinical trial no. NCT03258320) and infiltrating ductal breast cancer patients (clinical trial no. NCT02897700), by following the CONSORT 2010 Statement (updated guidelines for reporting parallel group randomized trials). Patients with a clinical stage ≥I subtype A (IA; T1a, N0, M0) of primary cancer but without manifest distant metastasis were enrolled into the multicentered, randomized, double‐blinded, and controlled pilot studies. Age between 40 and 75 years with histologically proven prostate cancer, or age ≥18 years with histologically proven infiltrating ductal breast cancer, was required for recruitment into the individual clinical cohorts. Data regarding tumor size, histological type, tumor penetration, lymph node metastasis, and TNM stage were obtained from the pathologic records. Before chemotherapy, tumors were acquired from these patients as “Pre” samples (an “Untreated” cohort). After chemotherapy, remaining tumors in patients were acquired as “Post” samples (a “Chemo‐treated” cohort, with most tumors collected within 1–6 months after treatment). For some cases, the “Pre” and “Post” tumor biopsies from the same individual patient were both accessible, and these samples were subject to further evaluation. Tumors were processed as FFPE biospecimens and sectioned for histological assessment, with alternatively prepared OCT‐frozen chunks processed via laser capture microdissection (LCM) for gene expression analysis. Specifically, stromal compartments associated with glands and adjacent to cancer epithelium were separately isolated from tumor biopsies before and after chemotherapy using an Arcturus (Veritas Microdissection) laser capture microscope following previously defined criteria (Sun et al., 2012). The immunoreactive scoring (IRS) gives a range of 1–4 qualitative scores according to staining intensity per tissue sample. Categories for the IRS include 0–1 (negative), 1–2 (weak), 2–3 (moderate), and 3–4 (strong; Fedchenko & Reifenrath, 2014). The diagnosis of prostate cancer and breast cancer tissues was confirmed based on histological evaluation by independent pathologists. Randomized controlled trial (RCT) protocols and all experimental procedures were approved by the Ethics Committee and the Institutional Review Board of Shanghai Jiao Tong University School of Medicine and Zhongshan Hospital of Fudan University, with methods carried out in accordance with the official guidelines. Informed consent was obtained from all subjects, and the experiments conformed to the principles set out in the WMA Declaration of Helsinki and the Department of Health and Human Services Belmont Report.

### 4.4 Histology and immunohistochemistry

Formalin‐fixed paraffin‐embedded tissue sections of 7 μm were deparaffinized in xylenes and rehydrated through a graded series of alcohols. Routine histology appraisal was performed with hematoxylin and eosin staining. For immunohistochemical (IHC) evaluation, FFPE sections underwent antigen retrieval with sodium citrate, incubation with 3% H_2_O_2_, and treatment with avidin/biotin blocking buffer (Vector Laboratories) and then 3% BSA for 30 min. Staining with primary and secondary antibodies was conducted at 4°C for overnight and at room temperature for 60 min, respectively. Sections were incubated with a H_2_O_2_‐diaminobenzidine (DAB) substrate kit (Vector, SK‐4100). Samples were counterstained with hematoxylin, dehydrated, and mounted. IHC images were obtained using an upright microscope (Olympus BX51). Brown staining indicated the immunoreactivity of samples.

### 4.5 In vivo SASP assessment of patients and ELISAs

Sections of clinical biospecimens or animal tissues were processed via LCM for gene expression analysis. Specifically, stromal compartments associated with glands in patient tumor samples were separately isolated using an Arcturus (Veritas Microdissection) laser capture microscope following the criteria defined formerly (Sun et al., 2012). For tumors grown from xenografts composed of human cells, OCT sections were first H&E‐stained to determine the location of stromal cells and the stroma–epithelium border; then, cell lineages were separately acquired by LCM. Transcript levels of human SASP canonical factors including IL‐6, IL‐8, WNT16B, SFRP2, MMP1, MMP3, and MMP10 were measured by qRT–PCR (primers listed in Table S6).

Peripheral blood samples from cancer individuals with matched FFPE or frozen tumor samples were collected in EDTA‐ or heparin‐coated tubes and centrifuged at 2,000 *g* for 10 min at room temperature within 1 hr of clinical acquisition to prepare high‐quality serum. AREG and IL‐8 proteins in serum of cancer patients were subject to quantification by antigen‐specific ELISA kits (R&D Systems, DAR00/DY208) according to the manufacturer's instructions. Detection limits for these factors were 20 and 40 pg/ml, respectively.

### 4.6 Immunodeficient animals and preclinical studies

All animals were maintained in a specific pathogen‐free (SPF) facility, with NOD/SCID (Charles River and Nanjing Biomedical Research Institute of Nanjing University) mice at an age of ~6 weeks (~20 g body weight) used. Ten mice were incorporated in each group, and xenografts were subcutaneously generated at the hind flank upon anesthesia mediated by isoflurane inhalation. Stromal cells (PSC27 or HBF1203) were mixed with cancer cells (PC3, LNCaP, or MDA‐MB‐231) at a ratio of 1:4 (i.e., 250,000 stromal cells admixed with 1,000,000 cancer cells to make tissue recombinants before implantation in vivo). Animals were sacrificed at 2–8 weeks after tumor xenografting, according to tumor burden or experimental requirements. Tumor growth was monitored weekly, with tumor volume (*v*) measured and calculated according to the tumor length (*l*), width (*w*), and height (*h*) by the formula: *v* = (π/6) × ((l + *w* + *h*)/3)^3^. Freshly dissected tumors were either snap‐frozen or fixed to prepare FFPE samples. Resulting sections were used for IHC staining against specific antigens or subject to hematoxylin/eosin staining.

For chemoresistance studies, animals received subcutaneous implantation of tissue recombinants as described above and were given standard laboratory diets for 2 weeks to allow tumor uptake and growth initiation. Starting from the 3rd week (tumors reaching 4–8 mm in diameter), MIT (0.2 mg/kg doses), DOX (doxorubicin, 1.0 mg/kg doses), therapeutic antibodies (cetuximab or AREG mAb, 10.0 mg/kg doses, 200 μl/dose), or vehicle controls were administered by body injection (chemicals via intraperitoneal route, antibodies through tail vein), on the 1st day of 3rd, 5th, and 7th weeks, respectively. Upon completion of the 8‐week therapeutic regimen, animals were sacrificed, with tumor volumes recorded and tissues processed for histological evaluation.

At the end of chemotherapy and/or targeting treatment, animals were anaesthetized and peripheral blood was gathered via cardiac puncture. Blood was transferred into a 1.5‐ml Eppendorf tube and kept on ice for 45 min, followed by centrifugation at 5,800 *g* for 10 min at 4°C. Clear supernatants containing serum were collected and transferred into a sterile 1.5‐ml Eppendorf tube. All serum markers were measured using dry‐slide technology on IDEXX VetTest 8008 chemistry analyzer (IDEXX). About 50 μl of the serum sample was loaded on the VetTest pipette tip followed by securely fitting it on the pipettor, and the manufacturer's instructions were followed for further examination.

All animal experiments were performed in compliance with NIH Guide for the Care and Use of Laboratory Animals (National Academies Press, 2011) and the ARRIVE guidelines, and were approved by the Institutional Animal Care and Use Committee (IACUC) of the University of Washington or Shanghai Institutes for Biological Sciences, Chinese Academy of Sciences.

### 4.7 Immune checkpoint blockade assays, human peripheral blood mononuclear cells, and humanized mice

Atezolizumab (a selective humanized monoclonal IgG1 antibody against PD‐L1), nivolumab (a humanized IgG4 anti‐human PD‐1), and corresponding human IgG1 or IgG4 as an isotype‐matched control (MCE) were used as therapeutic antibodies. Peripheral blood mononuclear cells (PBMCs) were isolated from 10 ml freshly collected clinical blood samples (approved by Institutional Review Board of Zhongshan Hospital of Fudan University, with informed consent obtained from all subjects).

Stromal CM was harvested from PSC27, PSC27‐BLEO, PSC27‐shRNA^C^‐BLEO, PSC27‐shRNA^AREG^#1‐BLEO, and PSC27‐shRNA^AREG^#2‐BLEO. Approximately 200,000 prostate cancer cells were seeded in 6‐well plates and subsequently cultured with stromal CM for 2 days. PD‐L1/PD‐L2 protein levels in prostate cancer cells were determined by immunoblots. Prostate cancer cells were alternatively treated with SB203580 (20 μM), erlotinib (100 nM), AG1478 (6 μM), LY294002 (4 μM), MK2206 (5 μM), rapamycin (200 nM), PD0325901 (500 nM), Bay 11‐7082 (2 μM), or ruxolitinib (10 μM) for 3 hr. Subsequently, prostate cancer cells were cultured with stromal CM supplemented with each inhibitor for 2 days, before immunoblot assessment of PD‐L1 expression. Freshly isolated and Ficoll‐purified (STEMCELL, #07851) human peripheral blood mononuclear cells (PBMCs) were cultured with stromal cell CM for 2 days, with PD‐1 protein expression analyzed with immunoblot.

Peripheral blood mononuclear cells were also cultured in RPMI 1640 media and activated by 500 U/ml IL‐2 (Novus, 27095‐1‐AP) for 3 days. Prostate cancer cells were cultured with stromal cell CM for 2 days before 100,000 cells were seeded in 24‐well plates. After the prostate cancer cells adhered to the wall, 1,000,000 activated PBMCs were added and co‐culture was maintained for 2 days. Levitated PBMCs were subject to PBS wash for twice, and the number of adherent prostate cancer cells was determined by hemocytometer.

Three‐ to 4‐week‐old Rag2^−/−^IL2Rγ^null^ mice (Jackson Laboratory) were injected with 7 × 10^6^ fresh human PBMCs via tail (i.v.) vein 3 days before tumor implantation, when a total of 1.2 × 10^6^ PC3 cells were injected alone or together with 0.3 × 10^6^ PSC27 cells subcutaneously (s.c.) into the flank of Rag2^−/−^IL2Rγ^null^ mice. At the beginning of the 3rd week, a time point when implanted cells were stably absorbed, animals were treated with atezolizumab (or nivolumab, each controlled by an isotype‐matched immunoglobulin, IgG1 for atezolizumab, IgG4 for nivolumab) alone or with AREG mAb (200 μl/dose) via i.v. injection. Mitoxantrone (MIT)‐based chemotherapy (0.2 mg/kg/dose) was performed on the same day of antibody injection, with the administration conducted on the 1st day of 3rd, 5th, and 7th weeks, respectively. These PBMC‐injected mice were bi‐weekly examined via the retro‐orbital route for human CD45+ cells in the peripheral blood, with a minimal of 25% human CD45+ cells considered qualified human PBMC‐circulating animal models. Blood samples were also evaluated for the concentration of human IFN‐γ and TNF‐α in plasma by antigen‐specific ELISA (BD Biosciences and PeproTech, detection cutoff per assay 5.0 pg/ml for IFN‐γ and 15.0 pg/ml for TNF‐α, respectively). Upon completion of the 8‐week therapeutic regimen, animals were sacrificed, with tumor volumes recorded and tissues subject to further assessment. Specifically, tumor‐infiltrating lymphocytes from each treatment group were assayed by flow cytometry, with number of human CD8+ T cells and regulator T cells per mg of tumor determined.

### 4.8 Statistical analysis

All in vitro experiments were performed in triplicates, and animal studies were conducted with *n* ≥ 8 mice per group. Sample sizes were estimated based on an 80% power to detect a 50% reduction in tumor volumes observed in mice subject to chemotherapy compared with control mice, accepted a type I error rate of 0.05. Animals were distributed into groups of equal body weight, and no animals were excluded from analysis. Unless otherwise indicated, data in the figures were presented as mean ± *SD*. Univariate and multivariate Cox proportional hazard model analyses were performed with statistical software SPSS. Statistical significance was determined by unpaired two‐tailed Student's *t* test, one‐ or two‐way ANOVA, nonlinear dose–response fitting curve for IC50 calculation with GraphPad Prism 5.0, Pearson's correlation coefficient test, Kruskal–Wallis test, Kaplan–Meier (log‐rank) test, Wilcoxon–Mann–Whitney test, or Fisher's exact test. For all statistical tests, a *p* value <.05 was considered significant.

## CONFLICT OF INTEREST

The authors declare that there is no conflict of interest.

## AUTHORS CONTRIBUTION

Y.S. conceived this study, designed the experiments, and supervised the project. Q.X. carried out most of the biological experiments. Q.L., D.Z., and D.F. acquired and analyzed clinical samples from prostate and breast cancer patients, and managed subject information. L.H. and Y.S. performed data mining and bioinformatics of gene expression and signaling pathways. F.C., B.Z., and M.Q. helped in vitro culture and phenotypic characterization of prostate cancer cells. J.G, J.X., L.C., Y.E.C., J‐P.C., E.W‐F.L., and J.C. provided conceptual inputs or supervised a specific subset of experiments. Q.X. and Y.S. performed preclinical studies. Y.S. prepared the manuscript. All authors critically read, commented on, and approved the final manuscript.

## Data Availability

The raw RNA‐Seq datasets generated during the current study have been deposited in the Gene Expression Omnibus database (accession code GSE108545). All sequencing experiments were performed as independent triplicates, and the RNA‐Seq data referenced during the study are available in a public repository (https://www.ncbi.nlm.nih.gov/geo/). The authors declare that all the other data supporting the findings of this study are available within the article or its Supporting Information, or from the corresponding author upon reasonable request.
