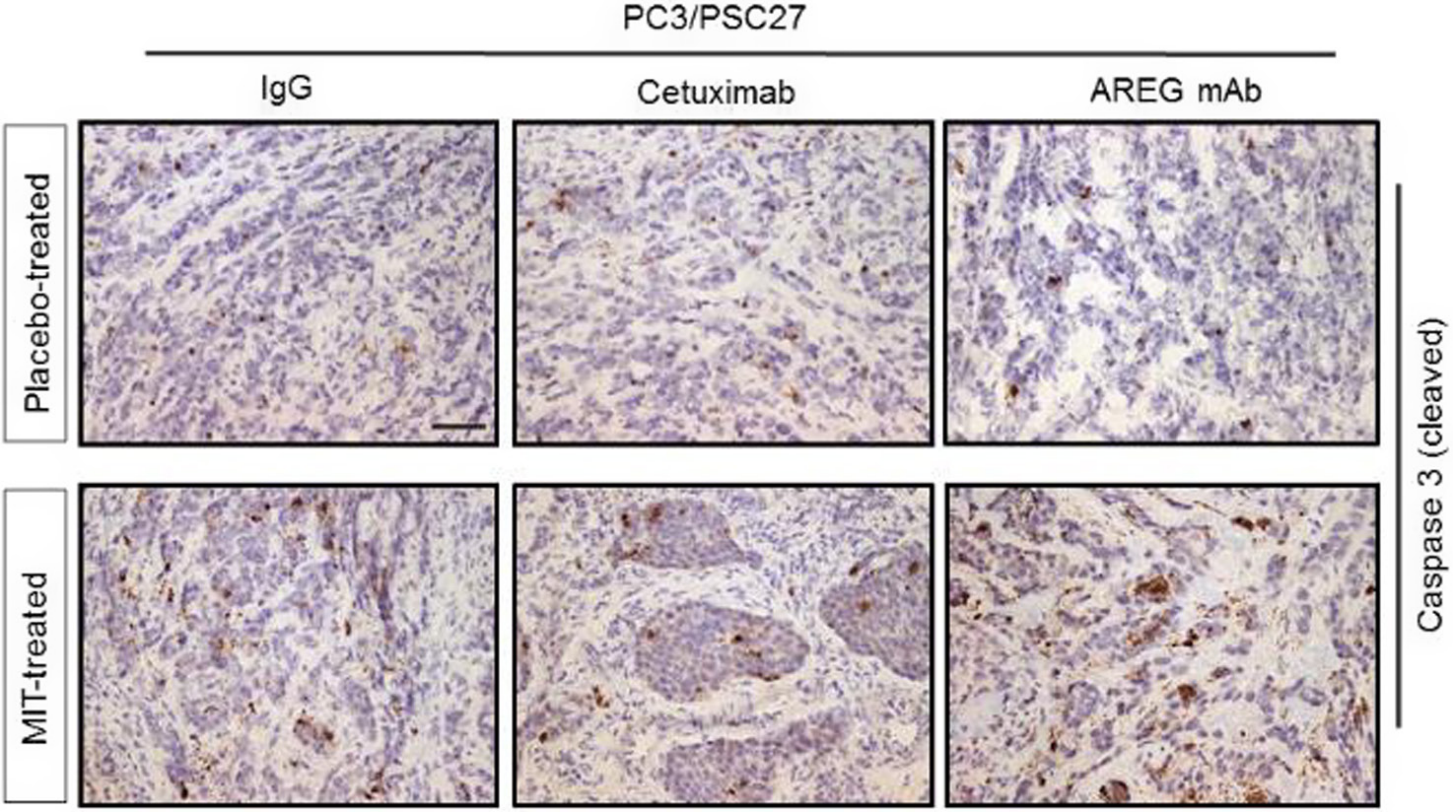# Correction to “Targeting amphiregulin (AREG) derived from senescent stromal cells diminishes cancer resistance and averts programmed cell death 1 ligand (PD‐L1)‐mediated immunosuppression”

**DOI:** 10.1111/acel.14248

**Published:** 2024-06-14

Xu Q, Long Q, Zhu D, et al. Targeting amphiregulin (AREG) derived from senescent stromal cells diminishes cancer resistance and averts programmed cell death 1 ligand (PD‐L1)‐mediated immunosuppression. *Aging Cell*. 2019;18:e13027. https://doi.org/10.1111/acel.13027

During the data organization and author preparation of this manuscript, there were a couple of errors inadvertently incorporated into the manuscript and not recognized effectively during the proofing stage. We noticed that the following item needs to be appropriately corrected.

Figure 5h. Representative IHC images of caspase 3 (cleaved) in tumors at the end of therapeutic regimes. The “Placebo‐treated AREG mAb” and “MIT‐treated Cetuximab” images were mistakenly picked up by authors to organize the original panel. As a necessary effort, the authors have now located the appropriate representative images and corrected this figure. Please refer to the updated Figure 5h.

All other parts of this article remain intact, valid, and unchanged. The authors sincerely regret the error and would like to apologize for any inconvenience this may have caused. The corrected figure is provided below. We apologize for this error.

Before correction

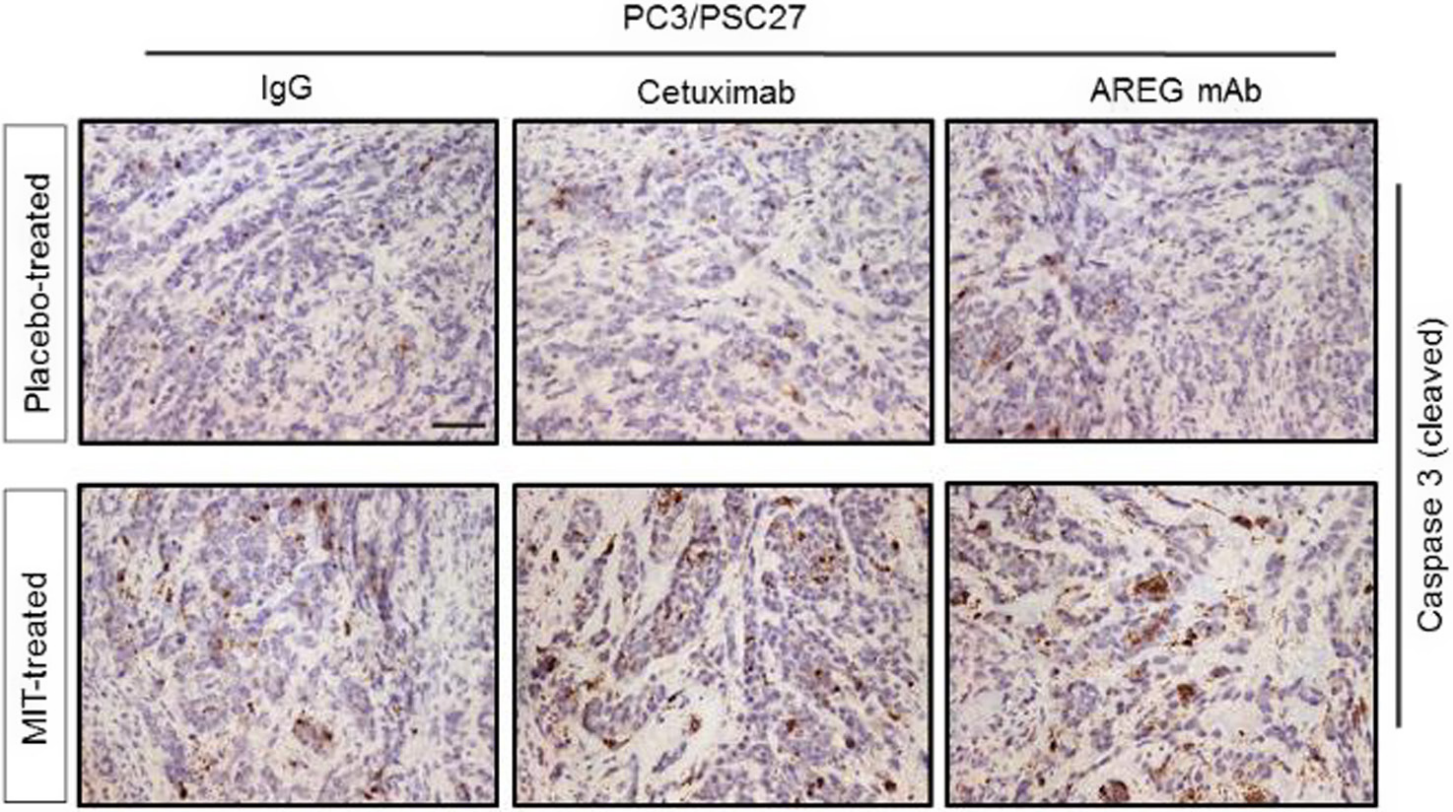

After correction