# Supplementary material for: Targeting amphiregulin (AREG) derived from senescent stromal cells diminishes cancer resistance and averts programmed cell death 1 ligand (PD‐L1)‐mediated immunosuppression
Source: Aging Cell. 2019 Sep 7;18(6):e13027. doi: 10.1111/acel.13027 (PMC6826133; doi:10.1111/acel.13027)
Supplement: Supplementary file 1 [file ACEL-18-e13027-s001.pdf]

SUPPORTING INFORMATION

Targeting AREG derived from senescent stromal cells diminishes cancer resistance and averts PD-L1-mediated immunosuppression

Qixia Xu, Qilai Long, Dexiang Zhu, Da Fu, Boyi Zhang, Liu Han, Min Qian, Jianming Guo, Jianmin Xu, Liu Cao, Y. Eugene Chin, Jean-Philippe Coppé, Eric W-F Lam, Judith Campisi, and Yu Sun

Figure S1

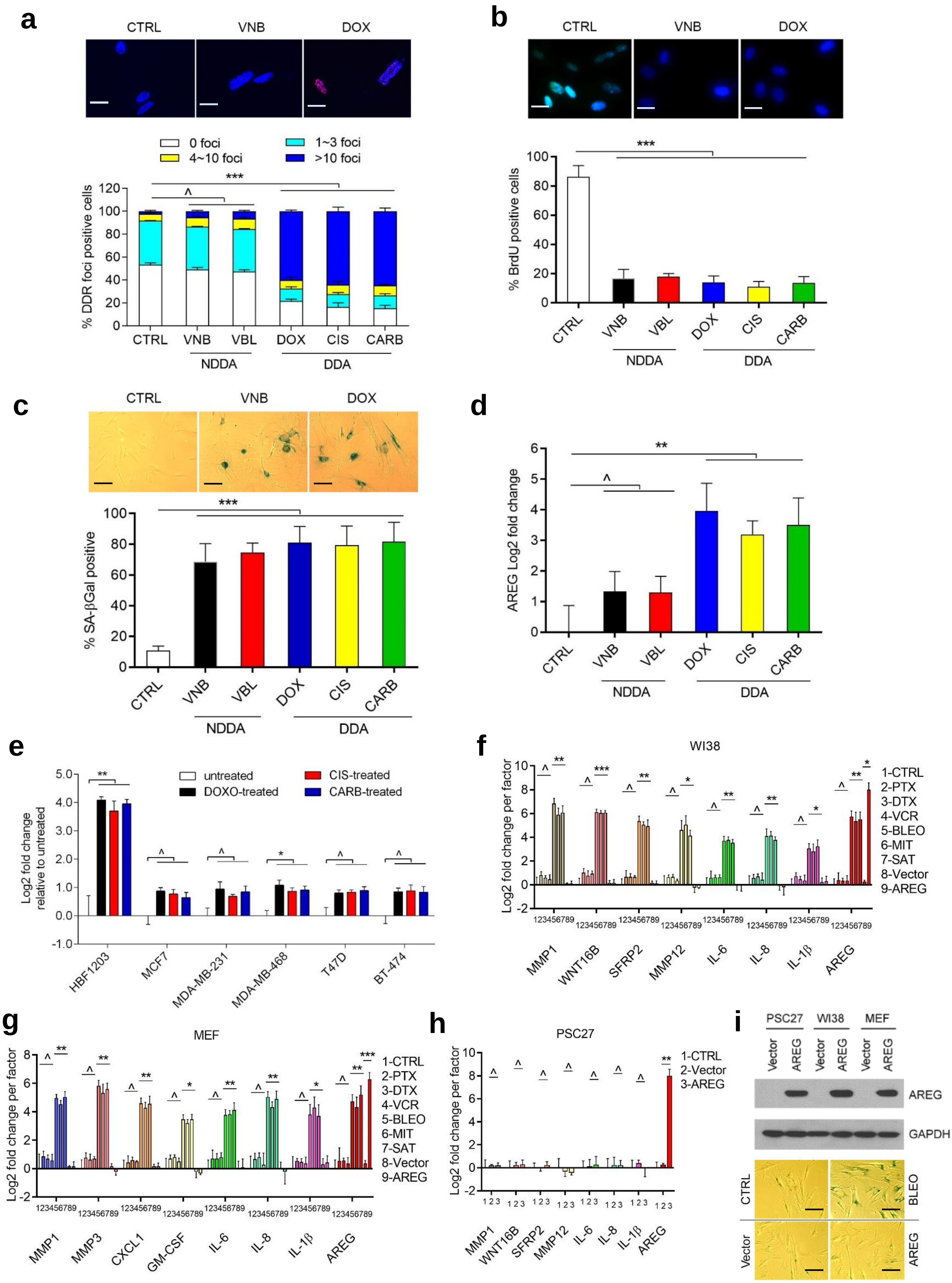
a
CTRL
VNB
DOX
0 foci
4~10 foci
1~3 foci
>10 foci
% DDR foci positive cells
CTRL
VNB
VBL
DOX
CIS
CARB
NDDA
DDA
b
% BrdU positive cells
c
% SA-βGal positive
d
AREG Log2 fold change
e
untreated
DOXO-treated
CIS-treated
CARB-treated
Log2 fold change relative to untreated
HBF1203
MCF7
MDA-MB-231
MDA-MB-468
T47D
BT-474
f
WI38
Log2 fold change per factor
1-CTRL
2-PTX
3-DTX
4-VCR
5-BLEO
6-MIT
7-SAT
8-Vector
9-AREG
MMP1
WNT16B
SFRP2
MMP12
IL-6
IL-8
IL-1β
AREG
g
MEF
MMP3
CXCL1
GM-CSF
h
PSC27
1-CTRL
2-Vector
3-AREG
i
PSC27
WI38
MEF
Vector
AREG
GAPDH
CTRL
BLEO

Figure S2

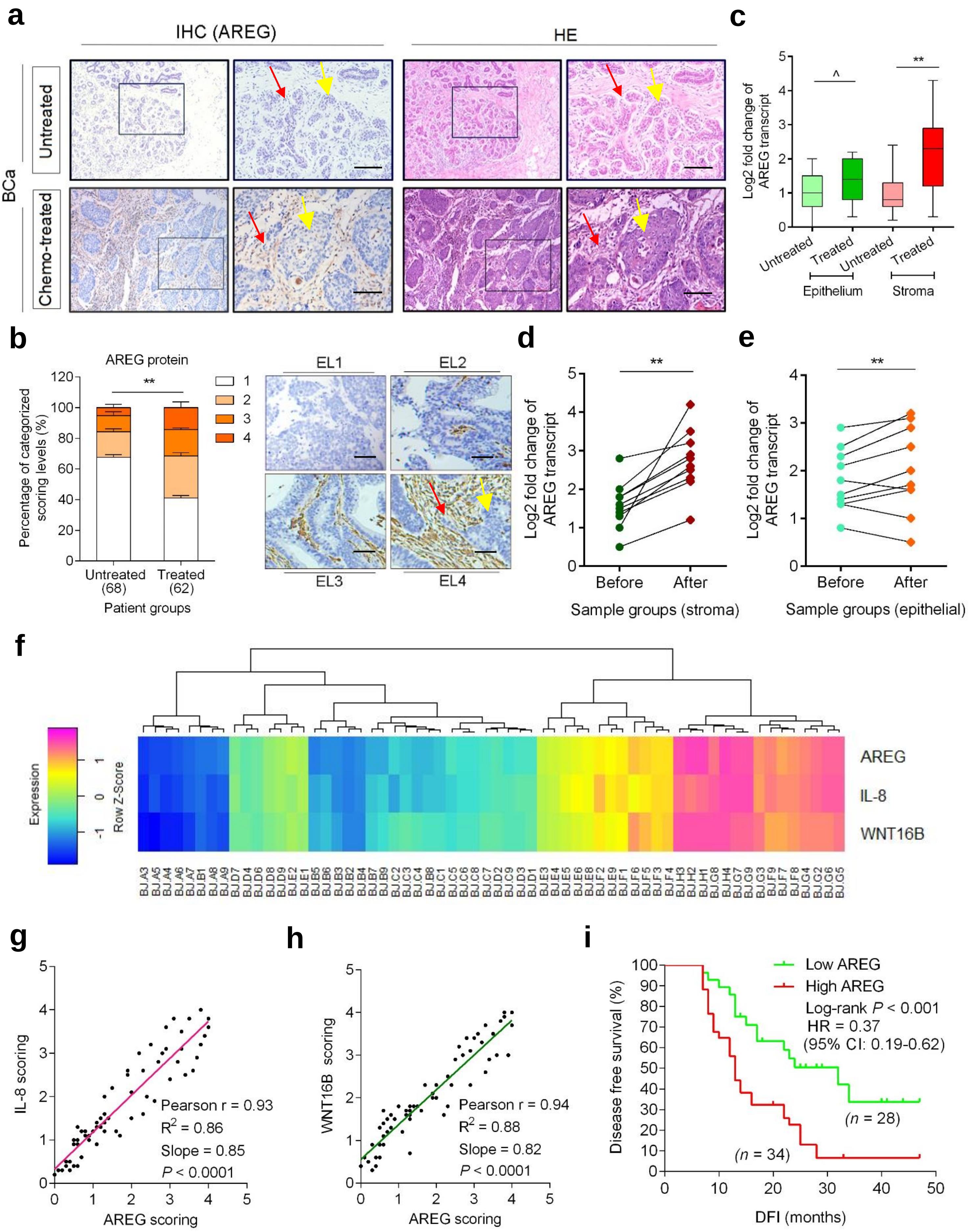

a
IHC (AREG)
HE
Untreated
Chemo-treated
BCa
b
AREG protein
Percentage of categorized scoring levels (%)
Untreated (68)
Treated (62)
Patient groups
EL1
EL2
EL3
EL4
c
Log2 fold change of AREG transcript
Untreated
Treated
Epithelium
Stroma
d
Log2 fold change of AREG transcript
Before
After
Sample groups (stroma)
e
Sample groups (epithelial)
f
Expression
Row Z-Score
AREG
IL-8
WNT16B
g
IL-8 scoring
AREG scoring
Pearson r = 0.93
R2 = 0.86
Slope = 0.85
P < 0.0001
h
WNT16B scoring
Pearson r = 0.94
R2 = 0.88
Slope = 0.82
P < 0.0001
i
Low AREG
High AREG
Log-rank P < 0.001
HR = 0.37
(95% CI: 0.19-0.62)
(n = 28)
(n = 34)
Disease free survival (%)
DFI (months)

Figure S3

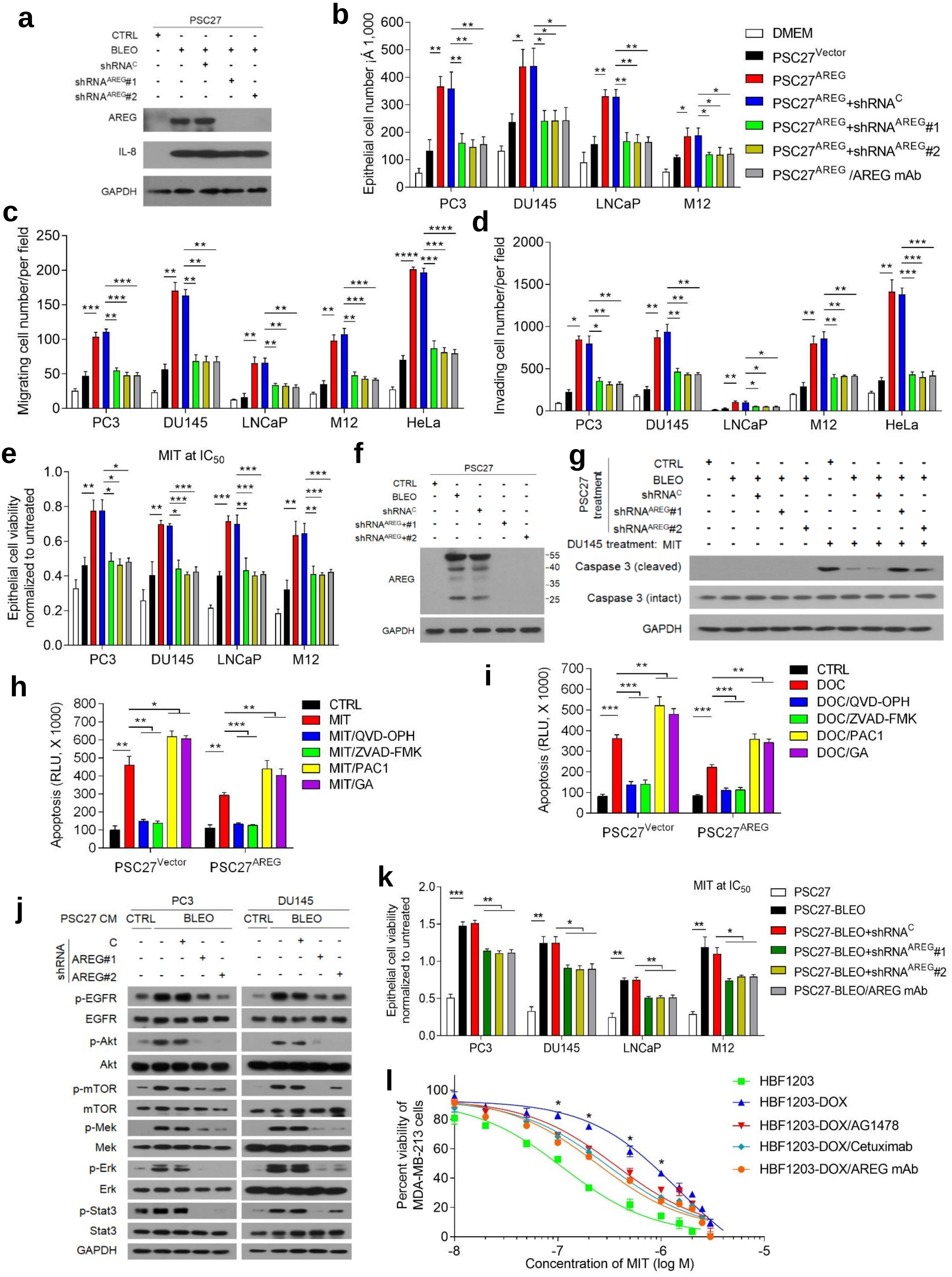

a
PSC27
CTRL
BLEO
shRNA^C
shRNA^AREG#1
shRNA^AREG#2
AREG
IL-8
GAPDH
b
Epithelial cell number ¡Á 1,000
PC3
DU145
LNCaP
M12
DMEM
PSC27^Vector
PSC27^AREG
PSC27^AREG+shRNA^C
PSC27^AREG+shRNA^AREG#1
PSC27^AREG+shRNA^AREG#2
PSC27^AREG /AREG mAb
c
Migrating cell number/per field
HeLa
d
Invading cell number/per field
e
MIT at IC50
Epithelial cell viability normalized to untreated
f
PSC27
AREG
GAPDH
g
PSC27 treatment
DU145 treatment: MIT
Caspase 3 (cleaved)
Caspase 3 (intact)
h
Apoptosis (RLU, X 1000)
CTRL
MIT
MIT/QVD-OPH
MIT/ZVAD-FMK
MIT/PAC1
MIT/GA
i
DOC
DOC/QVD-OPH
DOC/ZVAD-FMK
DOC/PAC1
DOC/GA
j
PC3
DU145
PSC27 CM
CTRL
BLEO
shRNA
C
AREG#1
AREG#2
p-EGFR
EGFR
p-Akt
Akt
p-mTOR
mTOR
p-Mek
Mek
p-Erk
Erk
p-Stat3
Stat3
GAPDH
k
MIT at IC50
PSC27
PSC27-BLEO
PSC27-BLEO+shRNA^C
PSC27-BLEO+shRNA^AREG#1
PSC27-BLEO+shRNA^AREG#2
PSC27-BLEO/AREG mAb
l
Percent viability of MDA-MB-213 cells
Concentration of MIT (log M)
HBF1203
HBF1203-DOX
HBF1203-DOX/AG1478
HBF1203-DOX/Cetuximab
HBF1203-DOX/AREG mAb

Figure S4

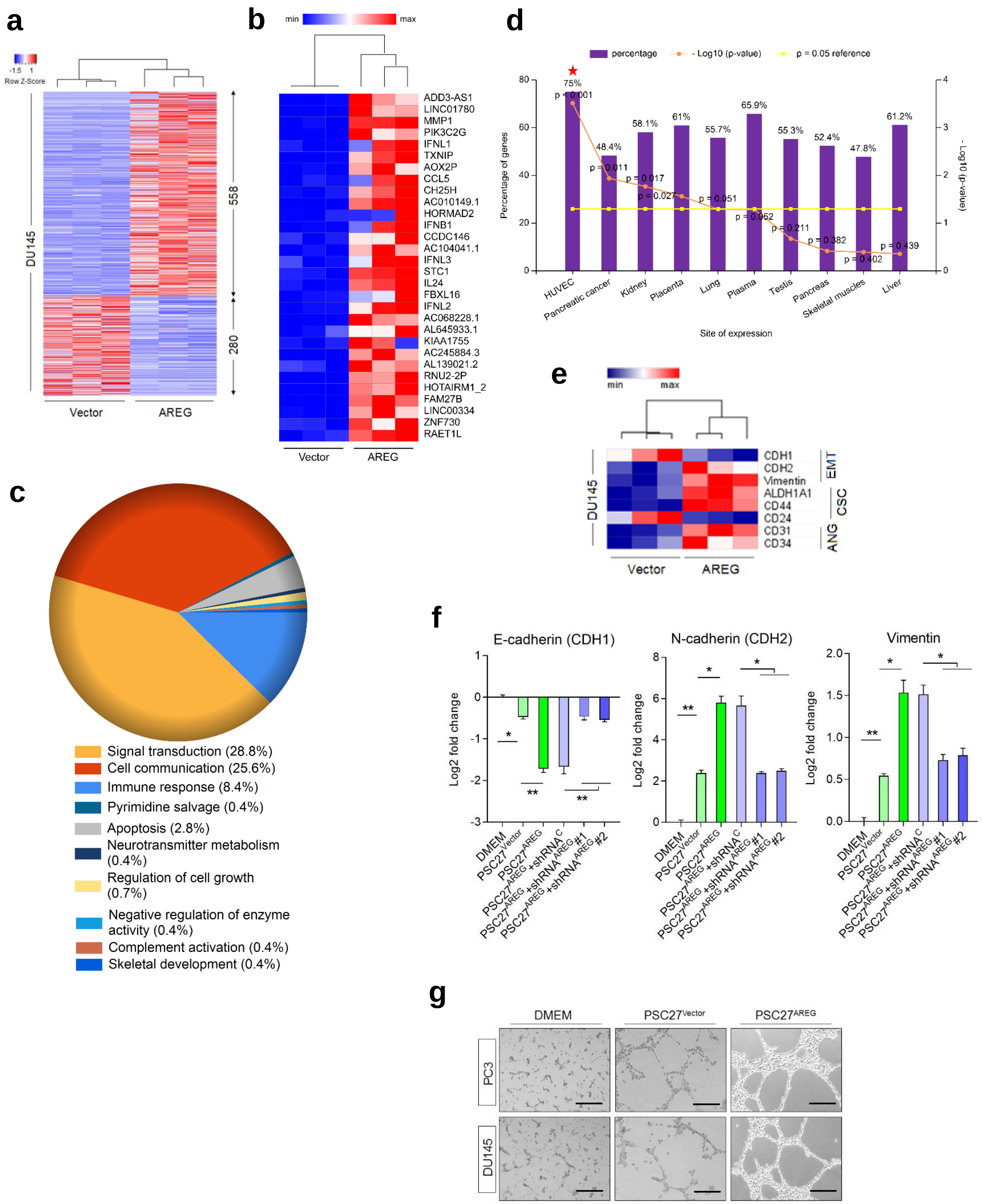

a
Row Z-Score
DU145
558
280
Vector
AREG
b
min
max
ADD3-AS1
LINC01780
MMP1
PIK3C2G
IFNL1
TXNIP
AOX2P
CCL5
CH25H
AC010149.1
HORMAD2
IFNB1
CCDC146
AC104041.1
IFNL3
STC1
IL24
FBXL16
IFNL2
AC068228.1
AL645933.1
KIAA1755
AC245884.3
AL139021.2
RNU2-2P
HOTAIRM1_2
FAM27B
LINC00334
ZNF730
RAET1L
Vector
AREG
c
Signal transduction (28.8%)
Cell communication (25.6%)
Immune response (8.4%)
Pyrimidine salvage (0.4%)
Apoptosis (2.8%)
Neurotransmitter metabolism (0.4%)
Regulation of cell growth (0.7%)
Negative regulation of enzyme activity (0.4%)
Complement activation (0.4%)
Skeletal development (0.4%)
d
percentage
- Log10 (p-value)
p = 0.05 reference
Percentage of genes
- Log10 (p-value)
Site of expression
HUVEC
Pancreatic cancer
Kidney
Placenta
Lung
Plasma
Testis
Pancreas
Skeletal muscles
Liver
75%
p < 0.001
48.4%
p = 0.011
58.1%
p = 0.017
61%
p = 0.027
55.7%
p = 0.051
65.9%
p = 0.052
55.3%
p = 0.211
52.4%
p = 0.382
47.8%
p = 0.402
61.2%
p = 0.439
e
min
max
DU145
CDH1
CDH2
Vimentin
ALDH1A1
CD44
CD24
CD31
CD34
EMT
CSC
ANG
Vector
AREG
f
E-cadherin (CDH1)
N-cadherin (CDH2)
Vimentin
Log2 fold change
DMEM
PSC27^Vector
PSC27^AREG
PSC27^AREG +shRNA^C
PSC27^AREG +shRNA^AREG #1
PSC27^AREG +shRNA^AREG #2
g
DMEM
PSC27^Vector
PSC27^AREG
PC3
DU145

Figure S5

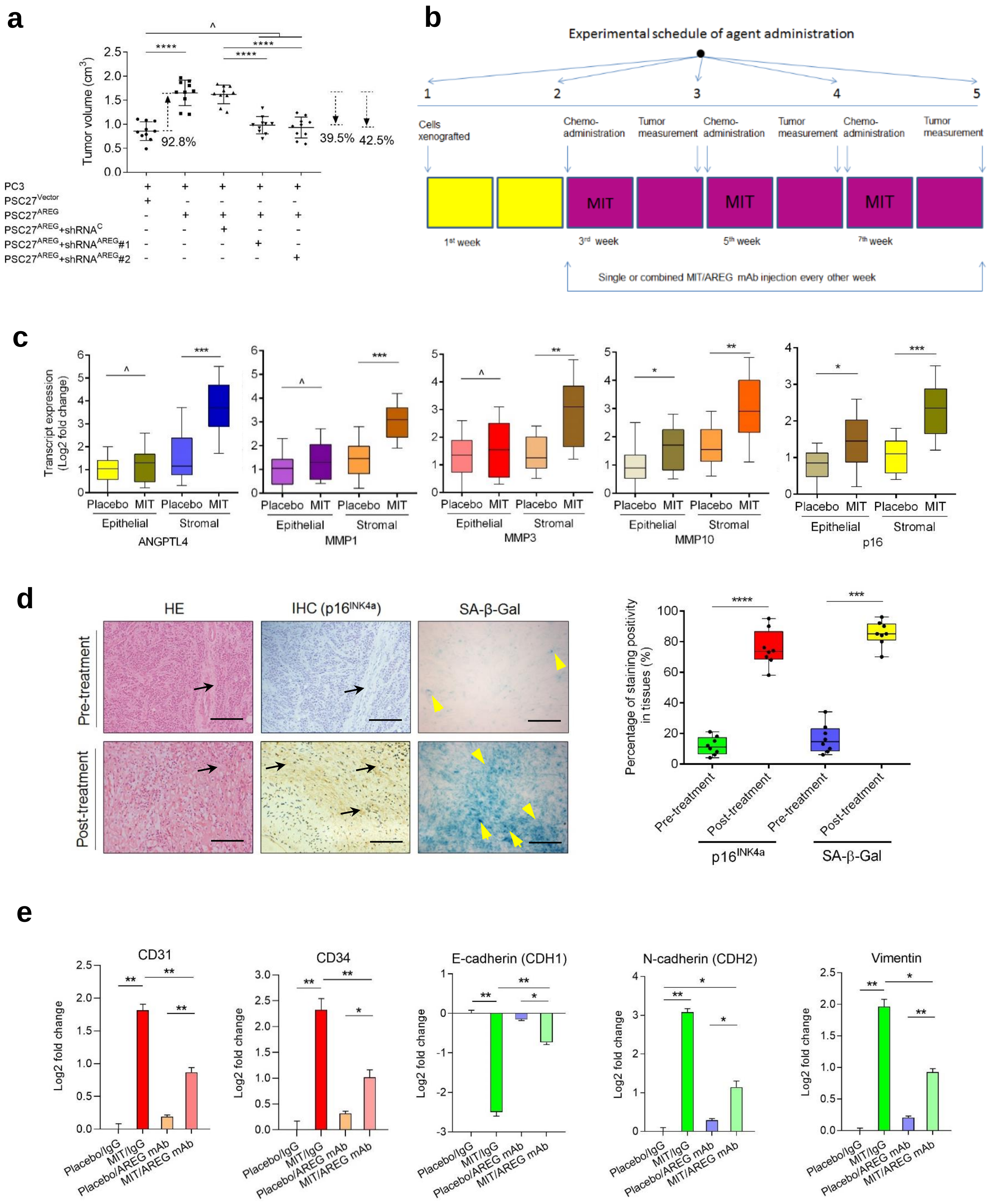
a
Tumor volume (cm3)
92.8%
39.5%
42.5%
PC3
PSC27Vector
PSC27AREG
PSC27AREG+shRNAC
PSC27AREG+shRNAAREG#1
PSC27AREG+shRNAAREG#2
b
Experimental schedule of agent administration
Cells xenografted
Chemo-administration
Tumor measurement
MIT
1st week
3rd week
5th week
7th week
Single or combined MIT/AREG mAb injection every other week
c
Transcript expression (Log2 fold change)
Placebo
MIT
Epithelial
Stromal
ANGPTL4
MMP1
MMP3
MMP10
p16
d
HE
IHC (p16INK4a)
SA-β-Gal
Pre-treatment
Post-treatment
Percentage of staining positivity in tissues (%)
p16INK4a
SA-β-Gal
e
CD31
CD34
E-cadherin (CDH1)
N-cadherin (CDH2)
Vimentin
Log2 fold change
Placebo/IgG
MIT/IgG
Placebo/AREG mAb
MIT/AREG mAb

Figure S6

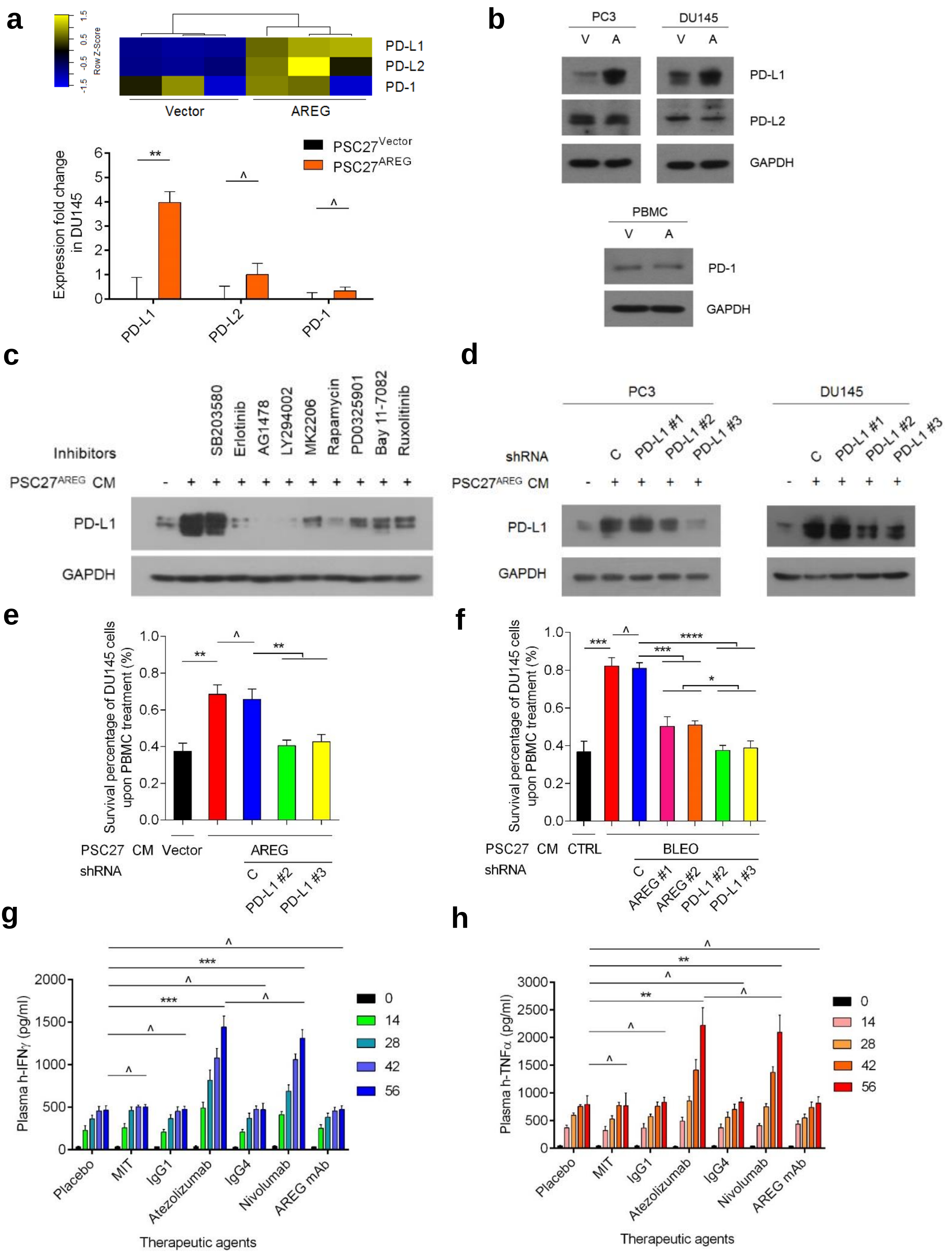

a
Row Z-Score
PD-L1
PD-L2
PD-1
Vector
AREG
Expression fold change in DU145
PSC27Vector
PSC27AREG
b
PC3
DU145
V A
PD-L1
PD-L2
GAPDH
PBMC
PD-1
c
Inhibitors
SB203580
Erlotinib
AG1478
LY294002
MK2206
Rapamycin
PD0325901
Bay 11-7082
Ruxolitinib
PSC27AREG CM
d
shRNA
C
PD-L1 #1
PD-L1 #2
PD-L1 #3
e
Survival percentage of DU145 cells upon PBMC treatment (%)
PSC27 CM
Vector
AREG
shRNA
f
CTRL
BLEO
AREG #1
AREG #2
g
Plasma h-IFNγ (pg/ml)
Placebo
MIT
IgG1
Atezolizumab
IgG4
Nivolumab
AREG mAb
Therapeutic agents
h
Plasma h-TNFα (pg/ml)

Figure S7

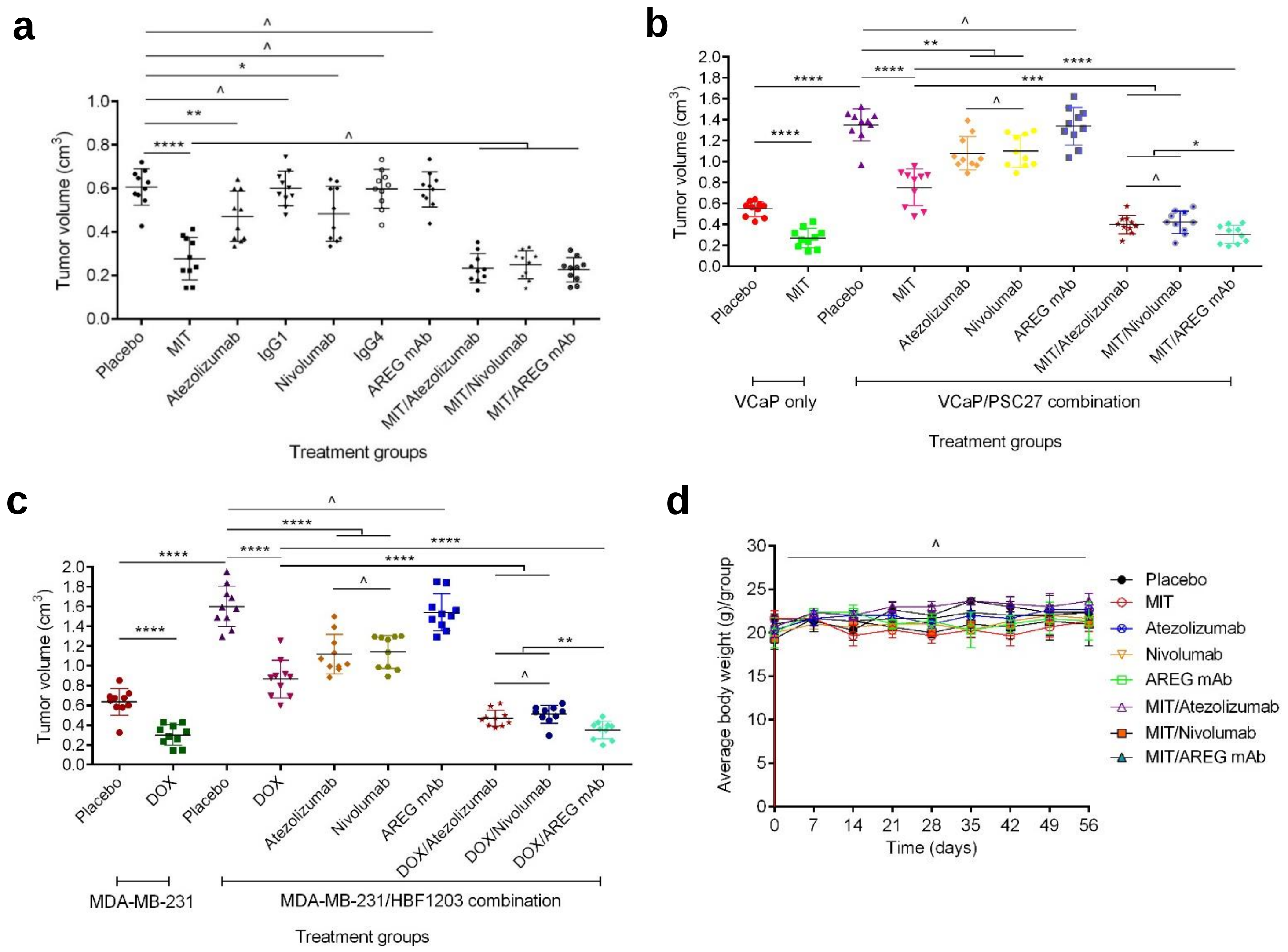
a
b
c
d
Tumor volume (cm3)
Placebo
MIT
Atezolizumab
IgG1
Nivolumab
IgG4
AREG mAb
MIT/Atezolizumab
MIT/Nivolumab
MIT/AREG mAb
Treatment groups
VCaP only
VCaP/PSC27 combination
DOX
DOX/Atezolizumab
DOX/Nivolumab
DOX/AREG mAb
MDA-MB-231
MDA-MB-231/HBF1203 combination
Average body weight (g)/group
Time (days)

Figure S8

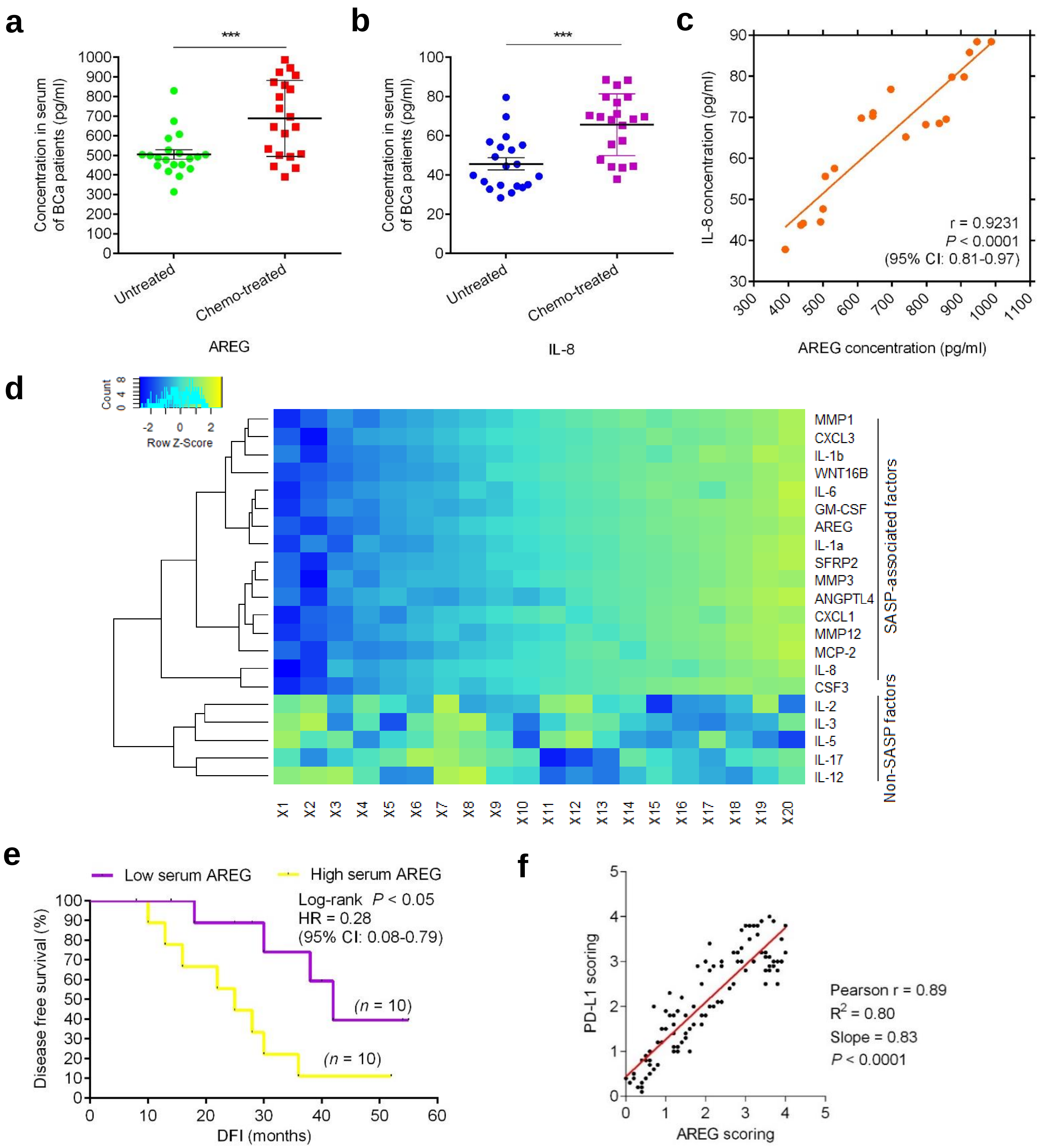

a
***
Concentration in serum of BCa patients (pg/ml)
Untreated
Chemo-treated
AREG
b
***
Concentration in serum of BCa patients (pg/ml)
Untreated
Chemo-treated
IL-8
c
IL-8 concentration (pg/ml)
r = 0.9231
P < 0.0001
(95% CI: 0.81-0.97)
AREG concentration (pg/ml)
d
Count
Row Z-Score
MMP1
CXCL3
IL-1b
WNT16B
IL-6
GM-CSF
AREG
IL-1a
SFRP2
MMP3
ANGPTL4
CXCL1
MMP12
MCP-2
IL-8
CSF3
IL-2
IL-3
IL-5
IL-17
IL-12
SASP-associated factors
Non-SASP factors
X1 X2 X3 X4 X5 X6 X7 X8 X9 X10 X11 X12 X13 X14 X15 X16 X17 X18 X19 X20
e
Low serum AREG
High serum AREG
Log-rank P < 0.05
HR = 0.28
(95% CI: 0.08-0.79)
(n = 10)
(n = 10)
Disease free survival (%)
DFI (months)
f
PD-L1 scoring
Pearson r = 0.89
R2 = 0.80
Slope = 0.83
P < 0.0001
AREG scoring

Figure S1. AREG expression is upregulated in senescent human breast stromal cells produced by genotoxic agents.

a) Representative immunofluorescence staining (IF) images (γH2AX, left) and comparative statistics (right) of DNA damage foci (DDR) in human breast stromal cell line HBF1203. Cells were treated by VNB (vinorelbine), VBL (vinblastine), DOX (doxorubicin), CIS (cisplatin) or CARB (carboplatin). DDR was profiled into 4 categories including 0 foci, 1~3 foci, 4~10 foci and > 10 foci per cell. CTRL, control. NDDA, non-DNA-damaging agents. DDA, DNA-damaging agents. Scale bars, 10 μm.
b) Representative images and comparative statistics of DNA incorporation assay of HBF1203 by BrdU staining. Cells were treated by different agents as applied in (**a**). Scale bars, 10 μm.
c) Representative images and comparative statistics of cellular senescence in HBF1203 treated by various agents used in (**a, b**). Cells were stained for SA-β-Gal activity 7 d after treatments. Scale bars, 15 μm.
d) Transcript expression of AREG in HBF1203 cells 7 d after treatment by several agents. Signal of each treatment condition was normalized to the CTRL group.
e) Comparative analysis of AREG transcript expression in breast stromal (HBF1203) and epithelial cells (MCF7, MDA-MB-231, MDA-MB-468, T47D and BT-474) of human breast origin. Signals normalized to untreated sample (CTRL) per cell line.
f) Quantitative transcript analysis of SASP factor expression in the human diploid fibroblast (HDF) line WI38 after treatment with a series of chemotherapeutic agents. Alternatively, AREG was overexpressed in a stable WI38 subline via lentiviral infection, with SASP expression assessed in parallel with other samples.
g) A similar set of transcript assays performed with the mouse embryonic fibroblast (MEF) line which was subject to various chemotherapeutic treatments. The AREG-overexpressing subline was analyzed in parallel.
h) Transcript examination of the PSC27 subline stably overexpressing AREG, with the individual SASP factors assessed. The vector-transduced subline was run as an experimental control.
i) Influence of AREG overexpression on stromal cell senescence. Upper, immunoblots of AREG expression in PSC27, WI38 and MEF sublines that stably express exogenous AREG. Bottom, SA-β-Gal assay for PSC27 cell subline transduced with a vector or the AREG cDNA, with CTRL/BLEO cells stained as experimental controls (genitive/positive). Scale bars, 20 μm.

Data are representative of 3 independent experiments, with 3 technical replicates run per cell-based assay. *P* values were calculated by Student's *t*-test (**a**, **b**, **c**, **d**, **e**, **f**, **g** and **h**) (^, $P > 0.05$; *, $P < 0.05$; **, $P < 0.01$; ***, $P < 0.001$).

Figure S2. AREG is expressed in the tumor microenvironment (TME) of breast cancer (BCa) patients after chemotherapy.

a) Representative images of AREG expression in the primary foci of human BCa patients. Left, immunohistochemical (IHC) staining. Right, hematoxylin and eosin (HE) staining. In each staining set, top tissues are from untreated patients; bottom tissues from treated. Rectangular region in the left image per staining is zoomed into the right image. Red arrows indicate stroma, yellow arrows epithelium. Scale bars, 100 μm.

b) Pathological assessment of stromal AREG expression in BCa patients (untreated, 68; treated, 62). Patients were pathologically assigned into 4 categories per AREG staining intensity in the stroma. 1, negative; 2, weak; 3, moderate; 4, strong. Left, statistical comparison of untreated and treated groups. Right, representative images of each category. ES, expression level. Red arrows indicate stroma, yellow arrows epithelium. Scale bars, 50 μm.

c) Comparative analysis of AREG expression at transcript level between different cell lineages after chemotherapy. Briefly, epithelial and stromal cells were separately acquired by laser capture microdissection (LCM) from the primary tumors of BCa patients, with RNA subsequently extracted for quantitative assessment.

d) Statistical appraisal of AREG induction in 10 randomly selected patients. Stromal cells from both before and after treatment per patient were isolated by LCM and subject to transcript examination.

e) Expression analysis of AREG in epithelial cells isolated from the 10 patients by following a procedure similar to that described in (**d**).

f) Pathological correlation between AREG, IL-8 and WNT16B in the stroma of BCa patients after chemotherapy. Scores were from the assessment of molecule-specific IHC staining, with expression levels colored to reflect low (blue) via modest (turquoise) and fair (yellow) to high (magenta) signal intensity. Columns represent individual patients, rows different SASP factors. Totally 62 patients treated by chemotherapy were analyzed, with scores of each patient averaged from 3 independent pathological readings.

g) Statistical correlation between AREG and IL-8 scores (Pearson analysis, $r = 0.93$; $P < 0.0001$) in the 62 tumors with matching protein expression data.

h) Statistical correlation between AREG and WNT16B scores (Pearson analysis, $r = 0.94$; $P < 0.0001$) in the same tumors as described in (**g**).

i) Kaplan-Meier analysis of BCa patients. Disease free survival (DFS) stratified according to AREG expression (low, average score < 2, green line, n = 28; high, average score ≥ 2, red line, n = 34). DFS represents the length (months) of period calculated from the date of treatment completion to the point of first time disease relapse. Survival curves were plotted according to the Kaplan–Meier method, while *P* value was calculated using a log-rank (Mantel-Cox) test.

Data are representative of 3 independent experiments. *P* values were calculated by Student’s *t*-test (**c**, **d**, **e**), one-way ANOVA (**b**) and log-rank test (**i**) (^, $P > 0.05$; **, $P < 0.01$). HR, hazard ratio.

Figure S3. Stromal cell-derived AREG alters the phenotypes of cancer cells.

- **a)** Immunoblot examination of AREG expressed in PSC27. Cells were infected with lentiviral particles encoding either the control shRNA ($shRNA^{C}$) or AREG-targeting shRNA ($shRNA^{AREG}$#1, #2), before treatment by bleomycin (BLEO) in culture. Expression of IL-8 was assessed as a parallel control to evaluate the influence of AREG or elimination on the SASP expression. GAPDH, loading control for protein lysates.
- **b)** PCa cell lines including PC3, DU145, LNCaP and M12 were treated with the conditioned media (CM) from PSC27 cells for 3 days, and subject to cell proliferation assay. $PSC27^{Vector}$ was used as control for cells exogenously expressing AREG ($PSC27^{AREG}$). Scramble and AREG-specific shRNAs were applied to generate stromal sublines as described in (**a**). Alternatively, an anti-AREG monoclonal antibody was employed to neutralize AREG in the CM before cancer cell phenotypic assays (as a control, also for **c**, **d** and **e**).
- **c)** Migration assessment of PCa cells after cultured for 3 days in the CM from sublines of PSC27. HeLa cell line was used as a positive control.
- **d)** Invasion assay of PCa cells across the basement membrane of transwells upon culture with the CM from sublines of PSC27 for 3 days. HeLa line served as a positive control.
- **e)** Chemoresistance assay of PCa cells upon culture with the CM from PSC27 sublines. MIT (mitoxantrone) was applied at the concentration of IC50 value pre-determined per cell line.
- **f)** Immunoblot appraisal of a monoclonal antibody generated specifically against human AREG. Lysates of PSC27 sublines established in (**a**) were loaded for analysis.
- **g)** Immunoblot analysis of protein lysates prepared from DU145, cultured with the CM of PSC27 sublines for consecutive 3 days in the presence or absence of MIT. Intact and cleaved caspase 3 was probed. GAPDH, loading control.
- **h)** Apoptotic assay for combined activities of caspase 3/7 determined 24 h after exposure of PC3 cells to CM of stromal cells while being treated by MIT in the presence or absence of caspase inhibitors including QVD-OPH and ZVAD-FMK, or caspase activators including PAC1 and gambogic acid (GA). RLU, relative luciferase unit.
- **i)** Apoptotic assay performed in the way similar as the one in (**h**), except that PAC (paclitaxel) was applied instead of MIT. RLU, relative luciferase unit.
- **j)** Immunoblot analysis of EGFR-associated pathways in PC3 and DU145 cells treated by the CM from PSC27 sublines as established in (**a**). Antibodies of p-EGFR, p-Akt, p-mTOR, p-Mek, p-Erk and p-Stat3were used, with the total protein per molecule and GAPDH probed as loading control.
- **k)** Chemoresistance assay of PCa cells cultured with the CM from PSC27 sublines after treatment by BLEO. MIT was applied at the concentration of IC50 value pre-determined per cell line. Alternatively, an anti-AREG monoclonal antibody

was employed to neutralize AREG in the CM before cancer cell phenotypic assays (as control).

l) Dose-response curves (non-linear regression/curve fit) plotted from drug-based survival assays of MDA-MB-231 cells cultured with the CM of HBF1203 native or damaged by doxorubicin (HBF1203-DOX), and concurrently treated by a wide range of concentrations MIT. AG1478 (6 μM), cetuximab (50 μg/ml) or AREG mAb (1 μg/ml) were applied with PSC27 CM.

Data are representative of 3 independent experiments. *P* values were calculated by Student's *t*-test (**b**, **c**, **d**, **e**, **h**, **i**, **k** and **l**) (^, $P > 0.05$. *, $P < 0.05$. **, $P < 0.01$. ***, $P < 0.001$).

Figure S4. Transcriptomic-wide expression of prostate cancer (PCa) cells is substantially modified by stromal AREG.

a) Heatmap showing differentially expressed human transcripts in DU145 cells after consecutive 3-day culture with AREG-containing CM collected from PSC27 cells. In contrast to cancer cells cultured with control media (Vector), there were 558 and 280 genes upregulated and downregulated, respectively, in those treated with $AREG^+$ stromal CM (AREG).

b) Heatmap depicting the top 30 transcripts in DU145 cells upregulated by paracrine AREG. Genes sorted according to their expression fold changes.

c) Pie chart displaying the biological processes (BPs) that are most pronouncedly associated with transcripts upregulated by AREG, as revealed by GO analysis of the top transcripts in DU145 line.

d) Column chart depicting the sites of expression of 558 transcripts upregulated in DU145 cells after AREG stimulation, with percentage and log10 (*P* value) per specific site indicated on the left and right Y axis, respectively. Red star, human umbilical vein endothelial cell (HUVEC).

e) Heatmap of gene expression signatures associated with phenotypic changes, namely EMT/CSC/ANG, after AREG exposure of DU145 cells *in vitro*. EMT, epithelial-to-mesenchymal transition; CSC, cancer stem cell; ANG, angiogenesis. Data derived from qRT-PCR assays.

f) Quantitative RT-PCR analysis of the transcript expression of EMT-specific markers E-cadherin, N-cadherin and vimentin in DU145 cells treated with the CM of PSC27 sublines. Data per factor were normalized to DMEM-cultured cells.

g) *In vitro* tube formation assay to measure the angiogenesis-inducing capacity of stromal AREG. PCa cells (PC3 and DU145) were seeded on top of a basement membrane matrigel layer and incubated in the CM of PSC27 sublines for 10 h. Cells were stained with calcein-AM and photographed by phase contrast microscopy. Scale bars, 100 μm.

Data are representative of 3 independent experiments. *P* values were calculated by Student's *t*-test (**f**) (^, $P > 0.05$; *, $P < 0.05$; **, $P < 0.01$).

Figure S5. Paracrine AREG confers therapeutic resistance on solid tumors developed in experimental animals.

a) Statistics of tumor volume measured at the end of an 8-week growth period. PC3 cells were xenografted alone or together with PSC27 cells to the hind flank of NOD/SCID mice. Prior to implantation, PSC27 cells were transduced with the control vector or AREG construct to make stable sublines ($PSC27^{Vector}$, and $PSC27^{AREG}$, respectively). $PSC27^{AREG}$ cells were subsequently subject to lentiviral infection to make AREG-null sublines ($PSC27^{AREG}$+$shRNA^{AREG}$ #1 and $PSC27^{AREG}$ +$shRNA^{AREG}$ #2), with scramble shRNA used as non-specific control ($PSC27^{AREG}$ +$shRNA^{C}$).

b) Strategic design of drug administration and tumor surveillance for preclinical trial. PC3 cells alone or combined with PSC27 cells were inoculated subcutaneously to NOC/SCID mice 2 weeks prior to the initiation of chemotherapy. The chemotherapeutic agent MIT was provided on the first day of each week starting from the 3rd week, then given every other week with a total number of 3 doses. Therapeutic antibodies (cetuximab or AREG mAb) were given 6 h before each time of MIT delivery (totally 3 doses in the regimen). At the end of 8 weeks mice were sacrificed with tumor volume measured, and histologically analyzed.

c) Transcript analysis of several canonical SASP factors including ANGPTL4, MMP1, MMP3 and MMP10 expressed in the epithelial and stromal cells, respectively, isolated from the tumors of mice. Expression of p16 was measured in parallel to determine occurrence of cellular senescence. Tumors were subject to laser capture microdissection (LCM)-based isolation, RNA preparation and qRT-PCR assays.

d) Representative images and statistics of *in vivo* cellular senescence after MIT-mediated chemotherapy. PC3/PSC27 tumors were collected at the end of therapeutic regimen and subject to histological assessment. Images from staining of HE, IHC (primary antibody anti-p16) and SA-β-gal staining were acquired for comparative analysis. Left, representative images. Black arrows, stromal cells. Yellow arrowheads, SA-β-gal-positive cells. Scale bars, 150 μm. Right, statistical presentation.

e) Quantitative RT-PCR analysis of CD31, CD34, E-cadherin, N-cadherin and Vimentin in LCM-isolated cancer cells from tumor tissues of experimental mice, which were subject to several different treatment conditions. Signals per gene were normalized to the average value of animal groups treated by placebo/IgG.

Data are representative of 3 independent experiments. N = 10 per treatment arm (n = 8 for data in **d**). MIT, mitoxantrone. P values were calculated by Student's t-test (**a**, **c**, **d, e**) (^, $P > 0.05$; *, $P < 0.05$; **, $P < 0.01$; ***, $P < 0.001$; ****, $P < 0.0001$).

Figure S6. Paracrine AREG activates immune checkpoint though upregulation of PD-L1 in recipient cancer cells.

a) Comparative analysis of PD-L1/PD-L2/PD-1 expression in DU145 cells cultured with CM of PSC27 cells transduced with a control (Vector) or AREG-expressing construct (AREG). Top, expression profile generated from RNA-Seq data. Bottom, qRT-PCR analysis of above gene expression in DU145 cells.

b) Immunoblot examination of immune check point-associated factors in PCa and immune cells. Top, PL-L1/PD-L2 expression in PCa cells. Bottom, PD-1 expression in PBMC cells upon culture with CM of PSC27 sublines. V, vector. A, AREG. GAPDH, loading control.

c) Immunoblot analysis of PD-L1 expression in PC3 cells upon culture with the CM of PSC27 sublines. Inhibitors against p38MAPK (SB203580), EGFR (erlotinib, AG1478), PI3K (LY294002), Akt (MK2206), mTOR (rapamycin), Mek1/2 (PD0325901), NF-kB (Bay 11-7082) or Jak1/2 (Ruxolitinib) was applied to culture individually. Cells were collected 3 days after initiation of treatment for lysis. GAPDH, loading control.

d) Immunoblot assessment of PD-L1 expression in PC3 and DU145 cells upon exposure to the CM of PSC27 sublines. Construct encoding scramble (C) and 3 PD-L1-specific shRNAs (#1, #2 and #3) were used to generate stable sublines prior to cancer cell culture with stromal CM. GAPDH, loading control. Note, #2 and #3 exhibited higher capacity in eliminating PD-L1 expression, and were selected for subsequent assays.

e) Survival evaluation of DU145 cells upon 3-day culture with either control (Vector) or AREG-containing (AREG) CM of PSC27 cells, in the presence of human peripheral blood mononuclear cells (PBMCs). D145 cells were lentivirally infected with scramble (C) or PD-L1-specific shRNAs (#2, #3) to make sublines prior to *in vitro* treatment.

f) DU145 sublines generated as above were subject to culture with CM of treatment-naive (CTRL) or bleomycin-damaged (BLEO) PSC27 cells, while human PBMCs were applied. Results were evaluated as the percentage of PC3 cells that survived 3 days of continuous culture. DU145 cells lentivirally infected with AREG-specific shRNAs (#1, #2) were examined as parallel controls.

g) Human interferon γ (IFNγ) in the sequential plasma samples of mice was subject to ELISA assessment. Rag2$^{-/-}$IL2Rγ^{null} mice were treated with placebo, MIT, atezolizumab, nivolumab or AREG mAb individually. IgG1 and IgG4 were administered separately as the IgG isotope for atezolizumab and nivolumab, respectively. Peripheral blood per treatment type was acquired sequentially from animals at 0, 14, 28, 42 and 56 days after human PBMC transplantation.

h) Plasma TNFα (h-TNFα) analysis in the sequential plasma samples of mice treated as described above.

Data are representative of 3 independent experiments. N = 10 per treatment arm for (**g**, **h**). *P* values were calculated by Student's *t*-test (**a**, **e**, **f**, **g**, **h**) (^, $P > 0.05$; *, $P < 0.05$; **, $P < 0.01$; ***, $P < 0.001$; ****, $P < 0.0001$).

Figure S7. Preclinical appraisal of tumor volume, body weight and metabolic biochemistry of experimental mice treated by chemotherapeutic and/or immunotherapeutic agents.

a) Statistical growth comparison of prostate tumors developed in Rag2$^{-/-}$IL2Rγ^{null} animals that were subject to different treatment modalities. Mice received xenografts composed of PC3 cells only, and underwent treatment by placebo, MIT, atezolizumab, nivolumab, AREG mAb as single agent, or MIT/ nivolumab, MIT/nivolumab, MIT/AREG mAb as combinatorial agents. IgG1 and IgG4 were administered separately as the IgG isotope for atezolizumab and nivolumab, respectively. Tumor volumes were measured at the end of the 8-week preclinical regimen.

b) Statistical comparison of the volumes of prostate tumors grown in Rag2$^{-/-}$IL2Rγ^{null} animals that experienced different treatment modalities as described above. Mice received VCaP cells implanted alone or combined with PSC27 cells. Tumor volumes were measured at the end of the 8-week preclinical regimen.

c) Statistical comparison of the volumes of breast tumors grown in Rag2$^{-/-}$IL2Rγ^{null} animals that underwent various modes of treatments. The chemotherapeutic agent was DOX (doxorubicin, 1.0 mg/kg doses), while mice received MDA-MB-231 cells implanted alone or combined with HBF1203 stromal cells. Tumor volumes were measured at the end of the 8-week preclinical regimen.

d) Mouse body weights were measured once a week until the end of the therapeutic regimen. Chemotherapeutic agent MIT (0.2 mg/kg) was administered alone or with the targeting antibody (atezolizumab, nivolumab or AREG mAb, each 10.0 mg/kg) on 1st day of week 3, 5 and 7 after tumor implantation (PC3/PSC27) to Rag2$^{-/-}$IL2Rγ^{null} mice.

Data are representative of 3 independent experiments. *P* values were calculated by Student's *t*-test (**a-d**) (^, $P > 0.05$; *, $P < 0.05$; **, $P < 0.01$; ***, $P < 0.001$; ****, $P < 0.0001$).

Figure S8. AREG is a novel and noninvasive biomarker that indicates the SASP development and immune checkpoint engagement *in vivo* of cancer patients.

a) Abundance of AREG protein in the serum of untreated and chemo-treated BCa patients. Data were derived from ELISA measurement and shown as mean ± SD, n = 20.

b) Abundance of IL-8 protein in patient serum analyzed in (**a**), data from ELISA assays and presented as mean ± SD, n = 20.

c) Scatterplot showing correlation between AREG and IL-8 in the serum of individual BCa patients studied in (**a**) and (**b**). Pearson's correlation coefficient, *P* value and confidence interval are indicated in the plot, n = 20.

d) Heatmap showing the relative expression of a panel of SASP signature factors in the tumor stroma of PCa patients, and the correlation of AREG/IL-8 with these factors (n = 20). Stromal cells in the tumor tissues were isolated via LCM and expression of each target factor was measured by qRT-PCR, with signals per factor group normalized to that of the sample showing the lowest expression value. A subset of inflammatory factors typically not considered as SASP components was examined as random control, including IL-2/3/5/12/17. Correlations of these factors are presented as dendrogram organized by hierarchical clustering.

e) Kaplan-Meier survival analysis of chemo-treated BCa patients. Disease free survival (DFS) stratified according to AREG expression in tumor stroma (low, average score < 2, purple line; high, average score ≥ 2, yellow line). DFS represents the length (months) of period calculated from the date of treatment completion to the point of first time disease relapse. Survival curves generated according to the Kaplan–Meier method, with *P* value calculated using a log-rank (Mantel-Cox) test. n = 10 per group.

f) Scatterplot showing correlation between AREG in the stroma and PD-L1 in the tumor of individual BCa patients. Pearson's correlation coefficient, *P* value and confidence interval are indicated in the plot, n = 62.

Data are representative of 3 independent experiments. *P* values were calculated by Student's *t*-test (**a-b**) and pearson analysis (**c, f**) (****P* < 0.001).

Table S1. Univariate and multivariate Cox proportional hazards model analysis of prognostic factors for PFS of PCa patients.

Variable	Univariate			Multivariate		
	HR	95% CI	*P*	HR	95% CI	*P*
Age: < 60 vs. ≥ 60	0.47	0.19–1.21	0.120	0.44	0.21–0.92	**0.036**
Tumor stage: IV vs. IIc-III	1.27	0.38–4.29	0.696	1.34	0.48–3.83	0.582
Tumor size: ≥ 3 vs. < 3 cm	1.52	0.67–3.44	**0.042**	2.07	1.08–4.27	**0.038**
AREG: low vs. high	0.72	0.29–1.52	**0.011**	0.48	0.15-1.28	**0.002**
Caspase 3 (cleaved): low vs. high	0.84	0.26–1.65	0.093	0.96	0.38–2.02	0.265
AR low vs. high	0.78	0.39-1.72	0.054	1.28	0.67-3.59	0.065

PFS, progression-free survival. PCa, prostate cancer. HR, hazard ratio. CI, confidence interval. AR, androgen receptor. $P < 0.05$ is statistically significant, with significant *P*-values highlighted in bold.

Table S2. Clinical characteristics of of PCa patients.

Pathological ID	Tumor size (cm)	Age	Race and gender	Genotoxic agent involved	Tumor stage	DFS (months)	AREG score (ave)	PD-L1 score (ave)	Survival (0/1)	AR
17S23471	2.5	40	Asian, M	Mitoxantrone	IIc	40	0.0	0.0	0	+
17S23593	1.8	48	Asian, M	Mitoxantrone	IIIa	48	1.0	0.9	0	-
17S25650	2.2	41	Asian, M	Satraplatin	IIc	34	1.6	1.8	0	+
17S26224	2.8	46	Asian, M	Mitoxantrone	IIc	28	1.0	1.2	0	+
17S26429	2.4	54	Asian, M	Satraplatin	IIc	14	1.0	1.4	0	+
17S26430	3.2	63	Asian, M	Mitoxantrone	IIIb	22	0.2	0.4	1	++
17S27532	4.0	72	Asian, M	Satraplatin	IV	17	0.5	0.8	1	+++
17S27648	2.6	55	Asian, M	Satraplatin	IIc	25	0.3	0.0	0	++
17S32049	2.9	41	Asian, M	Satraplatin	IIIa	14	0.8	1.0	0	+
17S32187	3.6	75	Asian, M	Mitoxantrone	IV	24	1.0	1.2	1	+
17S36746	2.1	73	Asian, M	Satraplatin	IIIa	18	1.4	1.6	0	-
17S37342	2.8	44	Asian, M	Satraplatin	IIc	29	1.5	1.8	0	+
17S39343	3.4	70	Asian, M	Mitoxantrone	IIIa	17	1.7	1.5	1	+
17S39349	3.5	71	Asian, M	Satraplatin	IV	7	1.7	1.9	1	+++
17S40343	2.8	44	Asian, M	Satraplatin	IIc	8	1.8	1.6	0	+
17S42349	3.5	62	Asian, M	Mitoxantrone	IV	12	1.8	1.5	1	+
17S45149	6.0	65	Asian, M	Mitoxantrone	IV	15	1.9	1.6	1	++
17S46318	4.5	58	Asian, M	Mitoxantrone	IIIa	13	1.9	1.5	1	+
17S46390	2.5	46	Asian, M	Mitoxantrone	IIc	26	1.1	1.4	0	-
17S47109	3.5	58	Asian, M	Mitoxantrone	IIIa	60	0.6	1.0	0	+
17S23481	4.0	61	Asian, M	Satraplatin	IIIb	22	2.0	2.4	1	++
17S23572	8.0	65	Asian, M	Mitoxantrone	IV	23	2.1	2.2	1	+++
17S25640	3.5	63	Asian, M	Mitoxantrone	IIc	25	2.3	1.0	0	+
17S26234	3.8	66	Asian, M	Satraplatin	IIIa	25	2.0	2.5	1	++
17S26459	2.5	54	Asian, M	Mitoxantrone	IIc	28	2.4	2.8	0	+
17S26466	5.2	68	Asian, M	Mitoxantrone	IIIb	9	2.5	2.2	1	+
17S27556	6.9	70	Asian, M	Satraplatin	IV	9	2.6	2.0	1	++
17S27688	4.4	72	Asian, M	Mitoxantrone	IIIa	9	2.7	3.0	1	+++
17S32055	6.4	56	Asian, M	Mitoxantrone	IV	8	2.9	2.5	1	+++
17S32381	3.9	71	Asian, M	Mitoxantrone	IIIa	13	3.0	3.0	1	+++
17S33756	5.0	75	Asian, M	Mitoxantrone	IV	7	3.0	2.2	1	+++
17S37442	3.5	73	Asian, M	Mitoxantrone	IIIb	8	3.1	2.6	1	+
17S38143	9.0	75	Asian, M	Mitoxantrone	IV	7	3.2	3.0	1	+++
17S39449	3.8	73	Asian, M	Satraplatin	IIc	16	3.3	2.5	1	+++

17S40140	4.3	63	Asian, M	Mitoxantrone	IIIc	13	3.3	3.8	1	+++
17S41234	2.5	66	Asian, M	Mitoxantrone	IIIa	7	3.5	3.2	1	+
17S42138	4.0	59	Asian, M	Satraplatin	IV	8	3.6	2.8	1	+
17S43518	2.5	48	Asian, M	Mitoxantrone	IIc	7	3.7	3.4	0	-
17S44490	5.5	61	Asian, M	Mitoxantrone	IV	16	3.8	1.9	1	++
17S45103	4.2	69	Asian, M	Satraplatin	IIIb	13	3.9	1.6	1	+
17S45206	3.8	73	Asian, M	Mitoxantrone	IIIa	12	4.0	3.5	1	-
17S46105	3.2	65	Asian, M	Mitoxantrone	IIc	10	2.2	2.6	1	+++
17S46236	4.6	72	Asian, M	Mitoxantrone	IIIa	8	2.3	2.8	1	+
17S47701	4.8	74	Asian, M	Mitoxantrone	IIIb	12	2.8	2.9	1	+++
17S47808	5.6	73	Asian, M	Satraplatin	IV	13	3.0	2.6	1	+++
17S48101	4.4	64	Asian, M	Satraplatin	IIIb	28	3.2	1.8	1	+++
17S481340	2.8	55	Asian, M	Satraplatin	IIc	50	3.8	2.9	0	+
17S491321	6.2	62	Asian, M	Mitoxantrone	IV	13	4.0	3.9	1	+++

Table S3. Univariate and multivariate Cox proportional hazards model analysis of prognostic factors for PFS of BCa patients.

Variable	Univariate			Multivariate		
	HR	95% CI	*P*	HR	95% CI	*P*
Age: < 60 vs. ≥ 60	0.39	0.15–1.04	0.096	0.42	0.19–0.82	**0.048**
Tumor stage: IV vs. Ia-III	1.39	0.32–4.02	0.508	1.30	0.38–3.65	0.498
Tumor size: ≥ 3 vs. < 3 cm	1.78	0.45–3.21	**0.046**	1.96	1.02–3.17	0.065
AREG: low vs. high	0.65	0.31–1.72	**0.009**	0.44	0.24-0.76	**0.004**
Caspase 3 (cleaved): low vs. high	0.67	0.25–1.89	0.228	1.56	0.39–3.22	0.106
Her2 high vs. low	2.45	1.76-3.12	**0.015**	1.85	1.23-2.79	**0.032**
ER high vs. low	1.20	0.52-1.67	0.102	1.34	0.58-1.85	0.098

PFS, progression-free survival. BCa, breast cancer. HR, hazard ratio. CI, confidence interval. Her2, human epidermal growth factor receptor 2. ER, estrogen receptor. $P < 0.05$ is statistically significant, with significant *P*-values highlighted in bold.

Table S4. Clinical characteristics of of BCa patients.

Pathological ID	Tumor size (cm)	Age	Race and gender	Genotoxic agent involved	Tumor stage	PFS (months)	AREG score (ave)	PD-L1 score (ave)	Survival (0/1)	ER	PR	HER2	Ki67
153402-17	1.5	36	Asian, F	Cisplatin	IIa	40	0.5	0.8	0	-	-	+	+
152935-13	2.6	44	Asian, F	Carboplatin	IIb	41	1.2	0.9	0	-	-	+	+
161894-16	2.9	35	Asian, F	Doxorubicin	IV	34	1.3	1.5	1	-	-	+	+
158061-9	2.8	48	Asian, F	Doxorubicin	IIa	28	0.2	0.0	0	+	-	+	+
158060-14	2.1	50	Asian, F	Cisplatin	IIa	14	0.0	0.5	0	+	-	+	+
187928-15	3.5	56	Asian, F	Cyclophosphamide	Ia	22	0.3	0.4	1	++	-	++	+
180393-22	4.6	62	Asian, F	Epirubicin	IIIa	17	0.6	0.8	1	-	-	-	+
190855-11	1.9	55	Asian, F	Epirubicin	IIa	25	0.3	0.4	0	++	+	-	+
166497-34	2.4	37	Asian, F	Cyclophosphamide	IIa	14	0.8	1.0	0	+	-	-	+
178259-14	3.9	71	Asian, F	Doxorubicin	IIa	24	1.3	1.1	1	-	-	+++	+
162598-16	1.6	29	Asian, F	Cisplatin	IIIa	18	1.4	1.8	0	-	-	++	+
183638-14	2.5	42	Asian, F	Doxorubicin	IIa	29	1.5	1.8	0	+	+	+++	+
187862-15	4.5	75	Asian, F	Cisplatin	IIb	17	1.6	1.9	1	+	-	-	+
190220-35	3.9	66	Asian, F	Fluorouracil	IV	7	1.8	2.0	1	+++	-	-	+
182175-13	4.5	39	Asian, F	Doxorubicin	IIa	8	1.9	1.6	1		-	+++	+
184543-11	4.2	45	Asian, F	Doxorubicin	IV	13	1.8	1.2	1	-	+	+++	+
179751-16	5.9	60	Asian, F	Carboplatin	IIa	12	1.8	1.6	1	+	-	-	+
180720-9	5.6	54	Asian, F	Methotrexate	IIa	15	1.7	1.5	1	+	+	+	+
180718-18	5.4	42	Asian, F	Fluorouracil	IV	13	1.0	1.4	1	-	-	+	+
184776-28	3.1	54	Asian, F	Carboplatin	IIIa	26	0.5	0.8	0	+	-	-	+
180714-13	3.5	56	Asian, F	Doxorubicin	IIb	25	1.9	1.6	0	-	-	+++	+
179955-14	3.6	61	Asian, F	Doxorubicin	IIa	25	1.1	1.3	0	-	-	+++	+
183244-14	4.4	59	Asian, F	Cisplatin	IIb	13	1.3	1.0	1	-	-	+++	+
184023-16	5.8	65	Asian, F	Fluorouracil	IV	23	1.2	1.6	1	++	+	-	+
194682-15	6.5	50	Asian, F	Doxorubicin	IIb	10	1.4	1.8	1	+		+++	+
97769	2.5	62	Asian, F	Doxorubicin	IIIa	47	1.8	1.9	0	-	-	+++	+
113871	4.4	66	Asian, F	Cisplatin	IIb	32	1.6	1.2	1	-	-	+++	+
112048	2.6	64	Asian, F	Doxorubicin	IIIb	44	1.7	1.9	0	+++	+++	+++	+
112009-2	6.5	52	Asian, F	Doxorubicin	IV	22	2.0	2.8	1	+++	++	+	+
112009-32	3.3	67	Asian, F	Cisplatin	IIIa	12	3.4	3.0	1	+++	++	+	+
112523	5.5	71	Asian, F	Fluorouracil	IIIb	14	3.2	2.8	1	+++	-	-	+
113137	3.8	79	Asian, F	Methotrexate	IV	14	3.8	2.9	1	-	-	+++	++
113389	2.9	78	Asian, F	Cisplatin	IIIc	20	3.4	3.1	0	+++	-	++	+
113432	2.5	45	Asian, F	Doxorubicin	IIa	33	2.3	2.5	0	+++	+++	-	+

113515	4.8	67	Asian, F	Doxorubicin	IIIc	22	2.5	2.8	1	+++	+++	++	+
113668	5.2	62	Asian, F	Cisplatin	IV	23	2.4	2.7	1	-	-	-	+
114304	4.2	55	Asian, F	Carboplatin	IV	25	2.6	2.8	1	-	-	+++	+
114256	5.8	44	Asian, F	Carboplatin	IIIa	25	2.9	3.4	1	-	-	+++	+++
114083	6.2	54	Asian, F	Fluorouracil	IIa	28	3.8	3.4	1	-	-	-	++
113020-3	7.4	65	Asian, F	Methotrexate	IV	9	3.9	4.0	1	-	-	++	++
113020-27	3.8	66	Asian, F	Cisplatin	IIIb	9	4.0	3.8	1	-	-	++	++
114160	4.5	61	Asian, F	Cyclophosphamide	IIIb	9	2.4	2.0	1	+++	++	+	+
106548	5.8	74	Asian, F	Doxorubicin	IV	8	2.6	2.8	1	+	-	-	+
107125	4.3	77	Asian, F	Doxorubicin	IIIa	13	2.8	2.2	1	+++	+++	-	++
108766	5.2	60	Asian, F	Fluorouracil	IIb	7	3.1	3.4	1	+++	++	++	+
108862	4.9	62	Asian, F	Carboplatin	IIIb	8	3.2	3.6	1	+++	++	-	+
109377-13	2.9	51	Asian, F	Fluorouracil	IIb	7	2.3	2.5	1	-	-	-	+
102122	6.6	55	Asian, F	Cisplatin	IV	16	3.6	4.0	1	+++	+	+	+
103564	5.6	68	Asian, F	Carboplatin	IV	13	3.5	3.1	1	+++	++	++	+
103781	4.8	33	Asian, F	Carboplatin	Ia	7	2.1	2.3	1	++	-	+++	+
103853	5.3	48	Asian, F	Doxorubicin	Ia	8	2.5	2.2	1	-	-	+	++
104419	4.5	34	Asian, F	Cisplatin	IIa	7	2.4	2.8	1	-	-	+++	++
109377-4	6.8	56	Asian, F	Doxorubicin	IV	16	2.6	3.0	1	-	-	-	+
109994	4.9	48	Asian, F	Doxorubicin	IIIc	13	2.8	3.0	1	++	+	++	+++
110138	5.0	49	Asian, F	Doxorubicin	IIb	12	2.9	3.2	1	+++	+	++	++
110292	6.9	53	Asian, F	Cisplatin	IV	10	3.5	3.7	1	+	-	-	+
110780	3.8	56	Asian, F	Cisplatin	IIb	8	3.9	3.6	1	+++	+++	++	++
99559	4.4	58	Asian, F	Methotrexate	IIIa	12	2.8	2.5	1	+++	++	+	++
97922	6.8	64	Asian, F	Fluorouracil	IIb	13	3.1	2.9	1	+	-	-	+++
100430	2.9	46	Asian, F	Doxorubicin	IIa	47	3.5	3.7	0	-	-	++	+++
101372	3.3	76	Asian, F	Doxorubicin	IIa	25	3.8	4.0	1	++	+++	++	+
111628	4.9	32	Asian, F	Cyclophosphamide	IIIa	28	4.0	3.8	1	+++	-	++	+

Table S5. List of the top genes co-upregulated in PC3 and DU145 cells by stromal AREG with a fold change > 4.0 and FDR < 0.01.

Gene name	*PC3*		*DU145*	
	Fold change	***p value***	***Fold change***	***p value***
BCL2A1	10.18586	1.25E-08	12.78987	2.001E-55
KRTAP2-3	9.899441	8.36E-15	10.08427	5.99E-12
**CD274**	7.982082	1.89E-12	9.286984	2.54E-49
BARD1	7.272719	1.15E-8	7.318217	0.00008701
CORO1A	6.334301	1.52E-18	6.38224	6.91E-29
TAGLN3	6.115919	3.98E-05	6.155277	0.0000937
CXCL3	5.514699	2.43E-16	5.649783	2.28E-06
TNFAIP3	5.183835	5.02E-14	5.245726	9.33E-14
STC1	4.950016	2.85E-20	5.222908	1.79E-16
CSF2	4.773509	6.76E-12	5.017646	1.45E-14
NES	4.530654	1.36E-10	4.818856	1.12E-34
ESM1	4.373192	0.0000502	4.567708	3.65E-06

Table S6. List of quantitative RT-PCR primers.

Target name	***Forward (5'-3')***	***Reverse (5'-3')***
AREG	TGGATTGGACCTCAATGACA	AGCCAGGTATTTGTGGTTCG
IL-6	TACCCCCAGGAGAAGATTCC	TTTTCTGCCAGTGCCTCTTT
IL-8	GTGCAGTTTTGCCAAGGAGT	CTCTGCACCCAGTTTTCCTT
WNT16B	GCTCCTGTGCTGTGAAAACA	TGCATTCTCTGCCTTGTGTC
SFRP2	GCCTCGATGACCTAGACGAG	GATGCAAAGGTCGTTGTCCT
MMP1	GGTCTCTGAGGGTCAAGCAG	AGTTCATGAGCTGCAACACG
MMP3	GCAGTTTGCTCAGCCTATCC	GAGTGTCGGAGTCCAGCTTC
MMP12	ACACATTTCGCCTCTCTGCT	CCTTCAGCCAGAAGAACCTG
GM-CSF	CCCCAGTCACCTGCTGTTAT	TGGAATCCTGAACCCACTTC
ANGPTL4	GCCTATAGCCTGCAGCTCAC	AGTACTGGCCGTTGAGGTTG
IL-1a	AATGACGCCCTCAATCAAAG	TGGGTATCTCAGGCATCTCC
IL-1b	GGGCCTCAAGGAAAAGAATC	TTCTGCTTGAGAGGTGCTGA
CXCL1	AGGGAATTCACCCCAAGAAC	TGGATTTGTCACTGTTCAGCA
CXCL3	GCAGGGAATTCACCTCAAGA	GGTGCTCCCCTTGTTCAGTA
MCP-2	TCACCTGCTGCTTTAACGTG	ATCCCTGACCCATCTCTCCT
IL-2	TGCAACTCCTGTCTTGCATT	GCCTTCTTGGGCATGTAAAA
IL-3	CTTTGCCTTTGCTGGACTTC	CCGTCCTTGATATGGATTGG
IL-5	GAGACCTTGGCACTGCTTTC	CAGTACCCCCTTGCACAGTT
IL-12	GATGGCCCTGTGCCTTAGTA	TCAAGGGAGGATTTTTGTGG
IL-17	ACCAATCCCAAAAGGTCCTC	ACCAATCCCAAAAGGTCCTC
E-cadherin	TGCCCAGAAAATGAAAAAGG	GTGTATGTGGCAATGCGTTC
N-cadherin	GACAATGCCCCTCAAGTGTT	CCATTAAGCCGAGTGATGGT
Vimentin	GAGAACTTTGCCGTTGAAGC	TCCAGCAGCTTCCTGTAGGT
ALDH1A1	TGTTAGCTGATGCCGACTTG	TTCTTAGCCCGCTCAACACT
CD44	AGCAACCAAGAGGCAAGAAA	GTGTGGTTGAAATGGTGCTG
CD24	ACCCACGCAGATTTATTCCA	ACCACGAAGAGACTGGCTGT
CD31	GCAAAATGGGAAGAACCTGA	CACTCCTTCCACCAACACCT
CD34	CACCCTGTGTCTCAACATGG	GGCTTCAAGGTTGTCTCTGG
RPL13A	GTACGCTGTGAAGGCATCAA	CGCTTTTTCTTGTCGTAGGG
PD-L1	TATGGTGGTGCCGACTACAA	TGCTTGTCCAGATGACTTCG
PD-L2	GCAGAAGTATCCTGGCCAAA	GATGCAGAAGGGGATGAAAA
PD-1	GTGTCACACAACTGCCCAAC	CTGCCCTTCTCTCTGTCACC

Table S7. List of antibodies used for immunoblot, immunofluorescence and immunohistochemistry staining.

Antigen name	*Commercial source*	*Catalog number (clone number）*	*Application*
**AREG**	Proteintech	16036-1-AP	WB, IP, IHC
**IL-8**	Proteintech	60141-2-Ig	WB
**γH2AX**	Cell signaling	9718	IF
**BrdU**	Cell signaling	5292	IF
**EGFR**	Proteintech	18986-1-AP	WB, IP
**pEGFR**	Cell signaling	2231	WB
**mTOR**	Proteintech	20657-1-AP	WB
**pmTOR**	Abcam	ab1093	WB
**AKT1**	Proteintech	10176-2-AP	WB
**pAKT1**	Abcam	ab8932	WB
**Mek1/2**	Cell signaling	9122	WB
**p-Mek1/2**	Cell signaling	9121	WB
**Erk1/2**	Proteintech	16443-1-AP	WB
**pErk1/2**	Cell signaling	4377	WB
**STAT3**	Proteintech	10253-2-AP	WB
**pSTAT3**	Abcam	ab32143	WB
**E-cadherin**	Proteintech	20874-1-AP	WB, IF
**N-cadherin**	Proteintech	22018-1-AP	WB
**Vimentin**	Proteintech	60330-1-Ig	WB, IF
**GAPDH**	Vazyme	Ab103	WB
**Caspase 3 (cleaved)**	Cell signaling	9661	WB, IHC
**Caspase 3 (intact)**	Cell signaling	9662	WB
**PD-L1**	GenomeMe	IHC411	WB, IHC
**PD-L2**	Proteintech	18251-1-AP	WB, IHC
**PD-1**	GenomeMe	IHC001	WB, IHC

**CD68**	Abcam	Ab213363	IHC

SUPPLEMENTAL EXPERIMENTAL PROCEDURES

Vectors, Viruses and Infection

Full length human AREG sequence was cloned into pLenti-CMV/To-Puro-DEST2 as described (1). Small hairpin RNAs (shRNAs) targeting AREG (1#, sense strand 5’-CCGGTCCTGGCTATATTGTCGATGATCTCGAGATCATCGACAATATAGCCAG GTTTTTG-3’; 2#, sense strand 5’-CCGGTCACTGCCAAGTCATAGCCATACTCGAGTATGGCTATGACTTGGCA GTGTTTTTG-3’; scramble, sense strand 5’-CCGGTT-AGCGACTAAACACATCAATTCAAGAGATTGATGTGTTTAGTCGCTATTTTTT G’), and PD-L1 shRNAs (#1, sense strand 5’-CCGGTCGAATTACTGTGAAAGTCAATCTCGAGATTGACTTTCACAGTAAT TCGTTTTTG; #2, sense strand 5’-CCGGTCTGACATTCATCTTCCGTTTACTCGAGTAAACGGAAGATGAATGT CAGTTTTTG) were individually cloned in pLKO.1-Puro vector (Addgene). Upon production by 293T cells, lentivirual titers were adjusted to infect ~90% of cells. Stromal cells were infected overnight in the presence of polybrene (8 μg/ml), allowed to recover for 48 h and selected for 72 h before analysis. For expression of target genes in either stromal or epithelial cells, total RNA was prepared and subject to qRT-PCR assays (primers listed in Table S6).

Immunoblot and Immunofluorescence Analysis

Whole cell lysates were prepared using RIPA lysis buffer supplemented with protease/phosphatase inhibitor cocktail (Biomake). Nitrocellulose membranes were incubated overnight at 4°C with primary antibodies (listed in Table S7), with HRP-conjugated goat anti-mouse or -rabbit serving as secondary antibodies (Vazyme). For immunofluorescence analysis, cells were fixed with 4% formaldehyde and permeabilized before incubation with primary and secondary antibodies, each for 1 hour. Upon counterstaining with DAPI (0.5 μg/mL), samples were examined with an Imager.A1 (Zeiss) upright microscope to analyze specific gene expression.

In Vitro Cell Phenotypic Characterization

For proliferation assays of cancer cells, 2×10^4 cells were dispensed into 6 well-plates and co-cultured with conditioned medium (CM) from the stromal cells. Three days later, cells were digested and counted with hemacytometer. For migration assays, cells were added to the top chambers of transwells (8 μm pore), while stromal cell CM were given to the bottom. Migrating cells in the bottom chambers were stained by DAPI 12~24 hours later, with samples examined with Axio Observer A1 (Zeiss). Invasion assays were performed similarly with migration experiments, except that transwells were coated with basement membrane matrix (phenol red free, Corning).

Alternatively, cancer cells were subject to wound healing assays conduced with 6-well plates, with healing patterns graphed with bright field microscope. For EGFR pathway blockage, AG-1478 (MCE, HY-13524), a potent antagonist of EGFR, was added to a final concentration of 1μM in stromal CM. For chemoresistance assays, cancer cells were incubated with stromal cell CM, with the chemotherapeutic agent MIT provided in wells for 3 days at each cell line's IC50, a value experimentally predetermined.

Tube formation (*in vitro* angiogenesis) assay was based on the ability of reprogrammed cancer cells to form 3D capillary-like tubular structures on a basement membrane matrix. Briefly, PC3 and DU145 cells (3.5×10^4 cells/200 μl/cm^2) were suspended with LSGS-supplemented media 200PRF, loaded onto 12-well plates pre-coated with 50μl of Geltrex™ (Thermo Fisher). Plates were incubated at 37 °C for 8h and cells were stained with calcein-AM to measure cell viability and analyze tube formation. Cultures were photographed by phase contrast or fluorescence microscopy.

RNA-Seq and Bioinformatics Analysis

Total RNA samples were obtained from PC3 and DU145 cells cultured with CM of either $PSC27^{Vector}$ or $PSC27^{AREG}$. Sample quality was validated by Bioanalyzer 2100 (Agilent), and RNA was subjected to sequencing by Illumina HiSeq X 10 with gene expression levels quantified by the software package RSEM (https://deweylab.github.io/RSEM/). Briefly, rRNAs in the RNA samples were eliminated using the RiboMinus Eukaryote kit (Qiagen, Valencia, CA, USA), and strand-specific RNA-seq libraries were constructed using the TruSeq Stranded Total RNA preparation kits (Illumina, San Diego, CA, USA) according to the manufacturer's instructions before deep sequencing.

Paired-end transcriptomic reads were mapped to the reference genome (GRCh38/hg38) with reference annotation from Gencode v27 using the Bowtie tool. Duplicate reads were identified using the picard tools (1.98) script mark duplicates (https://github.com/broadinstitute/picard) and only non-duplicate reads were retained. Reference splice junctions are provided by a reference transcriptome (Ensembl build 73) (2). FPKM values were calculated using Cufflinks, with differential gene expression called by the Cuffdiff maximum-likelihood estimate function (3). Genes of significantly changed expression were defined by a false discovery rate (FDR)-corrected P value < 0.05. Only ensembl genes 73 of status "known" and biotype "coding" were used for downstream analysis.

Reads were trimmed using Trim Galore (v0.3.0) (http://www.bioinformatics.babraham.ac.uk/projects/trim_galore/) and quality assessed using FastQC (v0.10.0) (http://www.bioinformatics.bbsrc.ac.uk/projects/fastqc/). Differentially expressed

genes were subsequently analyzed for enrichment of biological themes using the DAVID bioinformatics platform (https://david.ncifcrf.gov/), the Ingenuity Pathways Analysis program (http://www.ingenuity.com/index.html). Raw data were preliminarily analyzed on the free online platform of Majorbio I-Sanger Cloud Platform (www.i-sanger.com), and subsequently deposited in the NCBI Gene Expression Omnibus (GEO) database under the accession code GSE116864.

Venn diagrams

Venn diagrams and associated empirical *P*-values were generated using the USeq (v7.1.2) tool IntersectLists (4). The t-value used was 22,008, as the total number of genes of status “known” and biotype “coding” in ensembl genes 73. The number of iterations used was 1,000.

RNA-seq heatmaps

For each gene, the FPKM value was calculated based on aligned reads, using Cufflinks (3). Z-scores were generated from FPKMs. Hierarchical clustering was performed using the R package heatmap.2 and the distfun = “pearson” and hclustfun = “average”.

Principal component analysis

Principal component analysis (PCA) was performed using the FPKM values of all ensembl genes 73 of status “known” and biotype “coding”.

Site of expression

Site of expression of differently expressed genes was assessed with FunRich, a stand-alone software designed for functional enrichment and interaction network analysis of genes and proteins in normal tissues, cancer tissues, cell types and cell lines (5).

Co-immunoprecipitation

Cells were rinsed twice with cold PBS then lysed on ice for 20 min in 1 ml of lysis buffer (40 mM HEPES at pH 7.5, 120 mM NaCl, 1 mM EDTA, 10 mM pyrophosphate, 10 mM glycerophosphate, 50 mM NaF, 0.5 mM orthovanadate, EDTA-free protease inhibitors) containing 0.3% CHAPS. Four micrograms of antibody specific to AREG (Proteintech) were added to the cleared cellular lysates and incubated with rotation for overnight. Then, 50 μl of protein A/G-agarose beads (Pierce) were added and the incubation continued for 12 h at 4 °C. Immunoprecipitates captured with the beads were washed thrice with the CHAPS lysis buffer and twice by wash buffer A (50 mM Hepes at pH 7.5, 150 mM NaCl,

protease phosphatase inhibitors included), and boiled in 4 × SDS sample buffer prior to electrophoresis and immunoblotting of protein samples.

Production and Purification of Recombinant Human AREG Protein

The EGF-like domain (aa 141–181) of human AREG ORF was subcloned downstream of the Met-Arg-Gly-Ser-His$_6$ (MRGSH$_6$) coding sequence between the BamHI/HindIII sites of the pQE9 expression vector (Qiagen). The new construct pQE9-AREG was sequenced to verify integrity, before production of a recombinant protein by M15 E. coli cells induced with 0.4 mM isopropyl-1-thio-D-galactopyranoside (IPTG) in culture. Bacterial lysates were centrifuged, with the supernatants purified by affinity chromatography using Co^{2+}-agarose resin (Qiagen). Bacterially expressed AREG EGF-like domain recombinant protein (~10 KD) was separated using Sephadex-G50 (Sigma) by size exclusion chromatography, and the product was then concentrated by ultrafiltration using Amicon Ultra NMWL 3KDa centrifugal filter units (Merck Millipore).

REFERENCES

1. Sun Y, Campisi J, Higano C, Beer TM, Porter P, Coleman I, *et al.* Treatment-induced damage to the tumor microenvironment promotes prostate cancer therapy resistance through WNT16B. Nat Med **2012**;18(9):1359-68.
2. Zerbino DR, Wilder SP, Johnson N, Juettemann T, Flicek PR. The ensembl regulatory build. Genome biology **2015**;16:56.
3. Trapnell C, Roberts A, Goff L, Pertea G, Kim D, Kelley DR, *et al.* Differential gene and transcript expression analysis of RNA-seq experiments with TopHat and Cufflinks. Nat Protoc **2012**;7(3):562-78.
4. Nix DA, Courdy SJ, Boucher KM. Empirical methods for controlling false positives and estimating confidence in ChIP-Seq peaks. BMC Bioinformatics **2008**;9:523.
5. Pathan M, Keerthikumar S, Ang CS, Gangoda L, Quek CY, Williamson NA, *et al.* FunRich: An open access standalone functional enrichment and interaction network analysis tool. Proteomics **2015**;15(15):2597-601.
